# Quorum Sensing in *Chromobacterium subtsugae* ATCC 31532 (Formerly *Chromobacterium violaceum* ATCC 31532): Transcriptomic and Genomic Analyses

**DOI:** 10.3390/microorganisms13051021

**Published:** 2025-04-29

**Authors:** Dmitry G. Deryabin, Ksenia S. Inchagova, Eugenia R. Nikonorova, Ilshat F. Karimov, Galimzhan K. Duskaev

**Affiliations:** 1 Federal Research Centre of Biological Systems and Agro-Technologies of the Russian Academy of Sciences, Orenburg 460000, Russia; ksenia.inchagova@mail.ru (K.S.I.); gatiatulinaer@gmail.com (E.R.N.); ifkarimov@yandex.ru (I.F.K.); 2 Institute for Cellular and Intracellular Symbiosis of the Ural Branch of Russian Academy of Sciences, Orenburg Federal Research Center of the Ural Branch of Russian Academy of Sciences, Orenburg 460000, Russia; 3 All-Russian Scientific Research Institute of Medicinal and Aromatic Plants (VILAR), Moscow 117216, Russia; 4 State Research Center of Dermatovenerology and Cosmetology of Russian Ministry of Health, Moscow 107076, Russia; 5 Department of Epidemiology and Infectious Diseases, Orenburg State Medical University of Russian Ministry of Health, Orenburg 460000, Russia

**Keywords:** *Chromobacterium subtsugae*, quorum sensing, transcriptome, QS-controlled genes

## Abstract

*Chromobacterium* spp. use a density-dependent cell-to-cell communication mechanism (quorum sensing, QS) to control various traits, including the pigment violacein biosynthesis. Recently, one of the type strains of this genus, previously deposited in the American Type Culture Collection under accession number *C. violaceum* 31532, was reclassified as *C. subtsugae*, making the QS data obtained for the first species irrelevant to the second. The goal of this study is to conduct transcriptomic and genomic analyses of the *C. subtsugae* ATCC 31532 (formerly *C. violaceum* ATCC 31532) strain to identify density-dependent regulated genes and the mechanisms of their QS control. Whole transcriptome dataset analysis comparing QS-negative mid-log phase and QS-positive early stationary phase samples revealed 35 down-regulated and 261 up-regulated genes, including 44 genes that increased transcription activity the most (log2 (fold change) > 4.0). In addition to the violacein biosynthesis, QS-controlled traits in *C. subtsugae* ATCC 31532 included the following: (i) *cdeAB-oprM* efflux pump; (ii) RND efflux transporter; (iii) *chuPRSTUV* iron acquisition system; (iv) polyamine transport system; (v) carbohydrate (semialdehydes) metabolic pathways; (vi) SAM/SPASM maturase system XYE (predicted); (vii) prophage proteins; and (viii) fucose-binding lectin II. Subsequent screening of the promoter regions of the up-regulated genes and operons in most cases showed the presence of CsuR AHL-receptor/transcriptional regulator binding sites with 56.25–68.75% similarity to the ideal 16-base-pair palindrome 5′-CTGTCCGATAGGACAG-3′ sequence, supporting the concept of QS control in *C. subtsugae* ATCC 31532 by the *csuI-csuR* gene pair. Notably, several transcriptional regulators (MarR, TetR/AcrR, HU family DNA-binding protein, helix-turn-helix domain-containing protein) were found to be under QS control. Based on these data, a hierarchical QS regulatory network in *C. subtsugae* ATCC 31532 was hypothesized that provides direct control of the target genes via a canonical autoinduction mechanism and further dissemination of the effect via the activity of QS-controlled transcriptional regulators.

## 1. Introduction

*Chromobacterium* is a genus of gram-negative, rod-shaped, facultative anaerobic bacterium, belonging to the family *Neisseriaceae*, order *Neisseriales*, class *Betaproteobacteria*, and phylum *Pseudomonadota* [1]. Currently, 11 species are known within this genus; among them, the type species is *Chromobacterium violaceum*, first described as free-living, purple-pigmented bacteria in the late 19th century [2]. There were two type strains of this genus, both deposited in the American Type Culture Collection (ATCC) under accession numbers 12472 and 31532 [3].

Increased attention to *Chromobacterium* spp. appeared in the late 20th century, when they were shown to use a density-dependent cell-to-cell communication mechanism (called “quorum sensing” (QS)) regulated by small diffusible signaling molecules termed autoinducers (AIs) [4]. In McClean et al.’s classic study [5], it was experimentally demonstrated that in the ATCC 31532 strain, the AI is N-hexanoyl-L-homoserine lactone, which is important for the purple pigment violacein biosynthesis. Surprisingly, in the ATCC 12472 strain, another N-(3-hydroxydecanoyl)-L-homoserine lactone was dominant, which also controlled violacein production via the QS phenomenon [6]. By consensus, both strains have similar autoinduction systems consisting of CviI (AHL synthase) and CviR (AHL receptor), where the dimeric CviR protein binds two AI molecules, changes from a closed to an open conformation, and recognizes specific DNA sequences in the promoter regions of QS-regulated genes [7].

This autoinduction mechanism activates the expression of previously silenced *vio*-operon consisting of five enzyme-coding genes (*vioA*, *vioB*, *vioC*, *vioD*, and *vioE*) [8]. In this enzymatic cascade, the flavoenzyme VioA and the heme protein VioB oxidize and dimerize L-tryptophan to an intermediate product that, in the presence of VioE, undergoes an indole rearrangement to prodeoxyviolacein. The last two enzymes in the pathway are the flavin-dependent oxygenase VioD, which hydroxylates one indole ring at the five position to yield proviolacein, and VioC, which then acts on the other indole ring at the two position to create the oxindole and complete the purple pigment violacein formation [9]. Notably, the level of violacein production in the ATCC 12472 strain was found to be much higher than that of the ATCC 31532 strain [5].

Since CviR directly controls the *vio*-operon, several *Chromobacterium* spp. strains have emerged as biosensors for the detection of pro-QS and anti-QS compounds, which is assessed by the presence or absence of bacterial pigmentation [10]. Among them, the most popular is an ATCC 31532-derived mutant (originally designated as CV026 biosensor strain [5]), which carries a mini-Tn5 insertion in the *cviI* gene and therefore produces violacein only in response to exogenous AHLs or AHL-mimic compounds [11].

An in-depth QS analysis was performed for the ATCC 12472 strain by Stauff and Bassler [12]. The ideal promoter DNA sequence required for recognition by CviR was found to be a CTGNCCNNNNGGNCAG 16-base-pair palindrome, and against this background, the direct QS activation of *vio*-operon, chitinase, and the type VI secretion-related gene, transcriptional regulator gene, and guanine deaminase gene, as well as the positive regulation of the *cviI* gene expression via positive feedback, were shown. Although the whole genome of the ATCC 31532 strain was subsequently sequenced [13], a similar search for the autoinduction mechanism was not repeated, and the main ideas about QS in *C. violaceum* were considered together.

In 2020, using the molecular phylogeny approach and multiple metabolic and phenotypic characters, ATCC 12472 and ATCC 31532 were found to belong to different bacterial species, and *C. violaceum* ATCC 31532 and its biosensor mutant CV 026 were reclassified as *Chromobacterium subtsugae* [14]. In accordance with this division, *C. violaceum* began to be considered as an environmental opportunistic pathogen of animals and humans [15], while *C. subtsugae* was seen as a pathogen of Colorado potato beetle and other insect pests [16].

Thus, the *C. violaceum* ATCC 12472 and *C. subtsugae* ATCC 31532 demonstrate multiple differences in the autoinducer type, the violacein production level, and pathogenic and other traits, so the QS data obtained for the first strain are irrelevant to the second.

The goal of this study is to conduct transcriptomic and genomic analyses of the *C. subtsugae* ATCC 31532 (formerly *C. violaceum* ATCC 31532) strain to identify density-dependent regulated genes and mechanisms of their QS control. The fundamental significance of this study is to clarify the lifestyle of the promising bioinsecticidal strain ATCC 31532, and the applied significance is to determine additional informative features for the evaluation of QS-modulating compounds using its derived biosensor strain.

## 2. Materials and Methods

### 2.1. Bacterial Strain and Growth Conditions

The wild-type *Chromobacterium subtsugae* Martin et al. 31532 strain (formerly *Chromobacterium violaceum* Bergonzini 31532 strain) was obtained from the American Type Culture Collection (ATCC Ltd., Glasgow, UK). This strain was grown in Luria–Bertani (LB) (Merck, NJ, USA) medium at 27 °C for 24 h. The optical density of the biomass was monitored at 450 ± 5 nm (OD_450_) using a multifunctional microplate reader Infinite 200 PRO (Tecan Group Ltd., Männedorf, Switzerland) in 200 µL aliquots at 1 h intervals. The pigment violacein biosynthesis was dynamically measured at 600 ± 5 nm (OD_600_) in ethanol extracts of biomass using the same equipment at identical time intervals.

**Figure 1:**
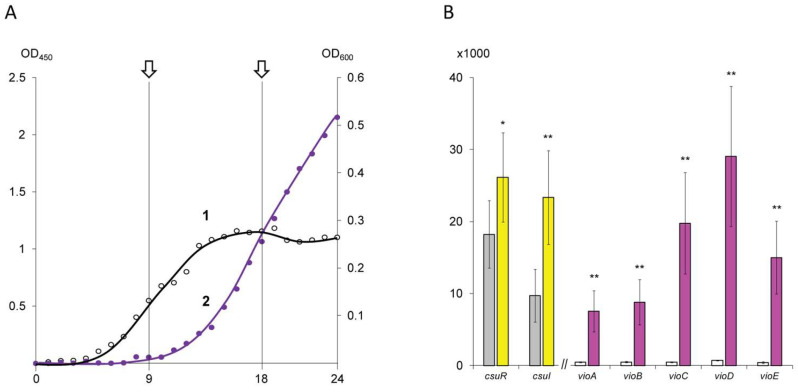
Phenotypic (**A**) and transcriptomic (**B**) characterization of *C. subtsugae* ATCC 31532 culture sampling points. In part (**A**): 1—bacterial biomass plot controlled by left scale (OD_450_); 2—pigment violacein plot controlled by right scale (OD_600_). The arrows indicate the sampling points at 9 h (QS−) and 18 h (QS+). In part (**B**): bar plot representing transcriptomic activity of QS-controlling (*csuR*, *csuI*) and QS-regulated (*vio*) genes in 9 h (left bars) and 18 h (right bar) samples. The ordinate axis shows normalized transcript counts. Values marked (*) differ significantly at *p* < 0.01; (**) at *p* < 0.001.

Based on monitoring data (Figure 1A), two points were selected for biomass collection. The first point (QS−) was chosen at 9 h, when the bacterial culture was in the middle logarithmic growth phase and the violacein biosynthesis was characterized by trace values. The second point (QS+) was established after 18 h, when the bacterial culture was in the early stationary phase and violacein production was occurring intensively. The collected biomass was separated from the culture medium at 4000 rpm for 5 min using a MiniSpin centrifuge (Eppendorf GmbH, Vienna, Austria), fixed in an IntactRNA reagent (Eurogen, Moscow, Russia), carefully pipetted and stored at –80 °C until RNA extraction.

### 2.2. RNA Extraction and Quality Control

Total RNA was isolated from bacterial cells using the Rizol reagent (diaGene, Moscow, Russia) according to the manufacturer’s protocol. RNA quality was controlled using the 2100 Bioanalyzer automated electrophoresis platform (Agilent Technologies, Santa Clara, CA, USA), which showed an RNA integrity number (RIN) greater than 9.0 for all samples. Further RNA quantification was performed using a Qubit 3.0 fluorometer (Thermo Fisher Scientific, Waltham, MA, USA) equipped with the manufacturer’s recommended Qubit RNA BR Assay kit. The QS− and QS+ points were analyzed in triplicate, and 1000 ng of total RNA was used for each sample.

### 2.3. cDNA Library Preparation

To enrich the nonribosomal RNA, rRNA was eliminated using the NEBNext rRNA Depletion kit (New England Biolabs, Ipswich, MC, USA). The method is based on specific ssDNA probes to rRNA, and the subsequent activity of the RNAse-H enzyme, which recognizes the DNA/RNA hybrids and destroys the rRNA. Further specific probe degradation by the DNAse I enzyme and purification of the target RNA with NEBNext RNA Sample Purification Bead were performed.

On the next step, the NEBNext Ultra II Directional RNA Library Prep Kit for Illumina (New England Biolabs, Ipswich, MC, USA) was used to prepare six cDNA libraries. To obtain cDNA from the RNA, reverse transcription was carried out using random primers, and the second chain was synthesized using dUTP. The purification of double-stranded cDNA using SPRIselect Beads was followed by end preparation and Illumina 3′-adapter ligation. Finally, PCR enrichment of the adaptor-ligated DNA was performed after subsequent purification with SPRIselect Beads.

The quality of the prepared cDNA libraries was assessed using a High Sensitivity DNA Chip on a 2100 Bioanalyzer (Agilent Technologies, Santa Clara, CA, USA).

### 2.4. cDNA Library Sequencing

Sequencing was performed on the NovaSeq6000 platform (Illumina Inc., San Diego, CA, USA) in the single-end read mode. Sample pooling and denaturation were carried out according to the “Denature and Dilute Libraries Guide” for the NovaSeq6000. The final loading concentration was 400 pM. The total sequencing data yield was 34 Gbp in FASTQ format. For each of the six samples, the yield was over 4.4 Gbp. The proportion of nucleotides with a read quality of at least 30 (Phred ≥ 30) for each sample were found to be more than 92%.

### 2.5. Bioinformatic Analysis of RNA-Seq Dataset

The Illumina 3′ adapter and poly-G sequences were trimmed using Cutadapt v. 4.2 [17], and the reads were filtered to a minimum length of 100 bp. The remaining sequences were aligned using the BWA MEM tool [18] with the complete genome data of *C. subtsugae* ATCC 31532 available in the NCBI database under the accession number NZ_CP142381.1 [19]. Prokka-processed gene functional annotations were obtained from the related NCBI resource [20]. The number of reads for each gene was evaluated using the featureCounts v. 2.0.1 program [21].

The DESeq2 v. 1.34.0 package [22] was used to analyze the differential gene expression between the QS− and QS+ samples. Normalized counts, *p*-value, and log2FoldChange (log2FC) were calculated for each of the 4550 genes. A heat map was constructed to view the gene expression patterns that significantly changed their transcription levels (log2FC > 2.0) and the Z-score distribution was determined for this RNA-seq dataset.

### 2.6. Bioinformatic Analysis of C. subtsugae ATCC 31532 Genome

Python-based scripts were used to determine the position of genes of interest, the direction of gene transcription, and the promoter region sizes. Selected genomic regions were extracted in the Browser Extensible Data (BED) format, and the promoter region sequences of the genes of interest were obtained using the BEDTools flank and BEDTools getfasta instruments [23]. The pairwise alignment of the promoter DNA sequences required for CviR recognition was performed using EMBOSS Needle software [24].

## 3. Results

### 3.1. Differential Expression of Genes Predicted to Be Under QS Regulation in C. subtsugae ATCC 31532 Strain

The whole transcriptome dataset analysis showed zero values for eight *rRNA* operons (ORF numbers U6115_02010–U6115_02030; U6115_02275–U6115_02295; etc.) due to the depletion procedure, while several *rrf* (5S ribosomal RNA) genes were found to be weakly expressed. Additionally, the *tRNA-Val* and *tRNA-Asp* gene cluster (ORFs numbers U6115_08410–U6115_08455) was silent, and no transcripts were detected for biopolymer transporter ExbD and MotA/TolQ/ExbB proton channel family protein genes (ORFs U6115_15075–U6115_15080 and U6115_15135–U6115_15140, respectively). The transcriptome repertoire of other 4,431 open reading frames was established as normalized counts for the QS− and QS+ samples, and log2FC and *p* values were calculated to characterize differential gene expression. The obtained data are presented in the Supplement S1.

**Figure 2:**
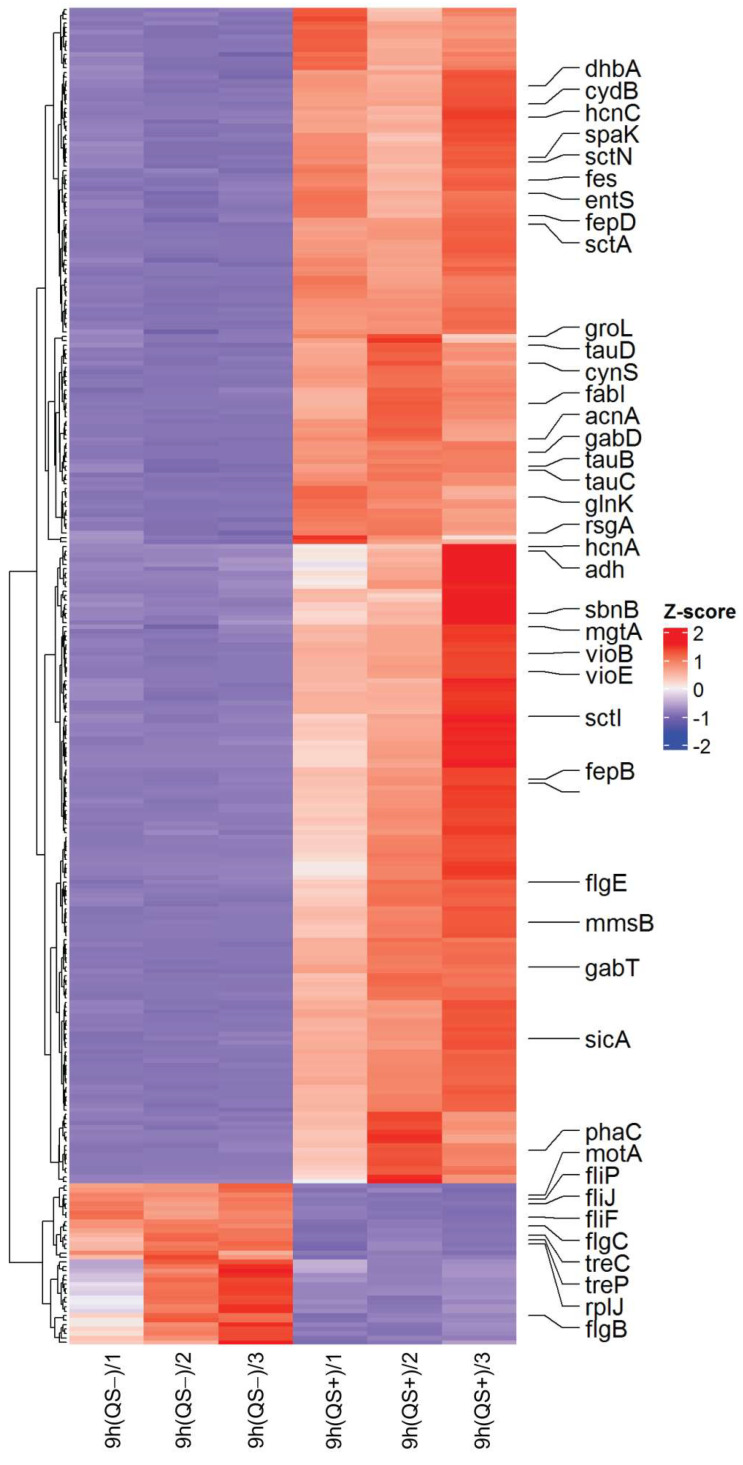
Heat map of RNA-Seq transcriptome analysis for 296 genes in *C. subtsugae* ATCC 31532 strain differently expressed (log2 (fold change) > 2.0) between 9 h (QS−) and 18 h (QS+) samples. Gene expression is visualized as the Z-score. The red and green colors represent the up-regulated and down-regulated genes, respectively. Specific gene names are shown.

In the first stage of searching for genes under QS regulation in the *C. subtsugae* ATCC 31532 strain, a heat map was constructed to visualize the expression values for the genes that showed a significant change in transcription level (log2FC > 2.0). As shown in Figure 2, the comparison of QS− and QS+ samples revealed 35 down-regulated and 261 up-regulated genes.

Among the down-regulated genes, genes encoding *tRNAs* were predominant (17 ORFs in total). Eight down-regulated genes related to bacterial motility were also detected, including *fliP* (ORF U6115_09380; flagellar type III secretion system pore protein), *fliF* (ORF U6115_09400; flagellar basal-body MS-ring/collar protein), *fliJ* (ORF U6115_09420; flagellar export protein), *motA* (ORF U6115_09455; flagellar motor stator protein), *flgC* (ORF U6115_16355; flagellar basal body rod protein), and *flgB* (ORF U6115_16360; flagellar basal body rod protein).

In turn, 261 up-regulated ORFs were represented by single genes or gene clusters associated with the functional bacterial cells differentiation.

Surprisingly, key genes involved in quorum sensing control by N-hexanoyl-homoserine-lactone biosynthesis (ORF U6115_21040; designated as *csuI*) and AHL reception/transcriptional regulation (U6115_21035; designated as *csuR*) were absent from the heat map chart. According to the transcriptomic data, these genes were already expressed at 9 h, and therefore their up-regulation by 18 h was characterized by log2 (fold change) values 1.2641 (*p* = 1.1 × 10^−15^) and 0.5189 (*p* = 2.8 × 10^−3^) only (Figure 1B).

Further analysis of the up-regulated gene list revealed a *vio*-operon, associated with pigment violacein biosynthesis (ORFs U6115_08050–U6115_08070) and previously characterized as directly QS-regulated [5]. This operon has canonical organization and consists of five genes: *vioA* (FAD-dependent oxidoreductase), *vioB* (iminophenyl-pyruvate dimer synthase), *vioC* (NAD(P)/FAD-dependent oxidoreductase), *vioD* (tryptophan hydroxylase), and *vioE* (violacein biosynthesis enzyme) [25]. Comparison of the 9 h and 18 h samples showed a dramatic increase in the *vio*-operon transcription level (Figure 1B), which was in good agreement with the phenotypic traits used to select the sampling points. Herewith, the calculated log2FC values for the violacein biosynthetic pathway genes were higher than 4.0, which allows us to consider this threshold as a cutoff for the QS-regulated genes. In some cases, co-expressed genes that showed a log2FC greater than 2.0 but less than 4.0 (which are present in the heat map but absent from the table) were also included in the further analysis.

According to the threshold of log2FC > 4.0, the *C. subtsugae* ATCC 31532 strain is additionally predicted to contain 39 genes under QS regulation. Their position in the bacterial genome, encoded product description, and differential expression data are presented in Table 1.

The most significant up-regulation was found for the ORF U6115_04630–ORF U6115_04665 cluster, containing eight genes, each of which showed extremely high (>7.0) Log2FC values. This operon includes a regulator protein (ORF U6115_04650), which is a member of the Multiple Antibiotic Resistance Regulator (MarR) family, as well as structural genes that encoded the efflux transporter outer membrane subunit = OprM (ORF U6115_04655), HlyD family efflux transporter periplasmic adaptor subunit = CdeB (ORF U6115_04660), and DHA2 family efflux MFS transporter permease subunit = CdeA (ORF U6115_04665). The obtained data confirm the QS-controlled regulation of the cdeAB-oprM genes in *C. subtsugae*, previously described by Koirala et al. [26].

In this study, we firstly reported that these genes are co-expressed with four other genes located upstream of the *marR* regulator. Among them, a gene encoding a AfsA-related hotdog domain-containing protein (ORF U6115_04635), a gene encoding a HAD-IB family hydrolase (ORF U6115_04640), and two genes with incompletely understood or unknown functions (ORF U6115_04630 and U6115_04645) were found. Interestingly, the AfsA family are key enzymes in A-factor (2-isocapryloyl-3R-hydroxymethyl-γ-butyrolactone) biosynthesis via catalysis acyl transfer between DHAP and a fatty acid derivative, which was originally considered as a microbial hormone controlling cellular differentiation and secondary metabolism in *Streptomyces* [27]. Another gene encoding a dehydrogenase superfamily (HAD superfamily) enzyme [28] may be also involved in the A factor biosynthesis via phosphatase activity. In turn, the upstream gene encodes a hypothetical protein with an unknown function, while in some other bacteria this position is occupied by the *bprA* gene involved in the reduction step for A factor biosynthesis [29]. The fourth over-expressed gene (ORF U6115_04645) encodes the domain of unknown function (DUF) 2165 family protein, which is predicted to be a small integral membrane protein [30].

The second operon previously characterized as QS regulated in *C. violaceum* ATCC 12472 [31] was the *chuPRSTUV* gene cluster. In *C. subtsugae* ATCC 31532, it is transcribed from the reverse strand and encodes the ABC transporter ATP-binding protein (ORF U6115_04815; = *chuV*), iron ABC transporter permease (ORF U6115_04820; = *chuU*), ABC transporter substrate-binding protein (ORF U6115_04825; = *chuT*), hemin-degrading factor (ORF U6115_04830; = *chuS*), TonB-dependent hemoglobin/transferrin/lactoferrin family receptor (ORF U6115_04835; = *chuR*), and hemin uptake protein HemP = ChuP (ORF U6115_04840). Two of these six genes (ORFs U6115_04820 and U6115_04840) showed 2.0 < log2FC < 4.0 values and therefore are present on the heat map (Figure 2) but absent from the Table 1 list. Overall, the activity of these genes determines the iron acquisition system required for heme/hemoglobin utilization and siderophore-mediated iron homeostasis.

The function of the other six QS-regulated gene clusters is less clear but can be proposed based on the encoded product description.

In the ORF range U6115_00115–U6115_00165, six genes met the log2FC cutoff criterion, and five genes showed 2.0 < log2FC < 4.0 values. This cluster contains a combination of genes for energy production/conversion (U6115_00135 encoded flavin reductase family protein; U6115_00140 encoded SDR family oxidoreductase; U6115_00160 encoded FAD-binding oxidoreductase) and fatty acid metabolism (ORF U6115_00130 encoded acyltransferase; U6115_00150 encoded cupin domain-containing protein; U6115_00155 encoded acyl-CoA dehydrogenase family protein; U6115_00165 encoded GNAT family N-acetyltransferase), as well as transport-related genes belonging to the RND family (U6115_00115 encoded efflux transporter outer membrane subunit; U6115_00120 encoded multidrug efflux RND transporter permease subunit; ORF U6115_00125 encoded efflux RND transporter periplasmic adaptor subunit; U6115_00145 encoded MFS transporter).

Downstream of this operon, ORF U6115_00170 was found, which is transcribed in the opposite direction and encodes a TetR/AcrR family transcriptional regulator showing the differential expression between QS− and QS+ samples log2FC = 3,6087. According to Ahn et al. [32], the observed gene arrangement corresponds to classification type I, where the *tetR* gene regulates a divergently expressed and described-above target operon.

The ORF U6115_13245–U6115_13255 gene cluster encoded two DUF1842 and one DUF1843 domain-containing protein which have not been functionally characterized, while the co-expressed ORF U6115_13260 is shown to encode a GDL motif peptide-associated radical SAM/SPASM maturase. Thus, it can be assumed that this operon belongs to the radical SAM/SPASM maturase system XYE, which is involved in peptide post-transcriptional and post-translational modifications [33] with a sibling property: violacein biosynthesis. This property can be explained by the fact that SAM (S-adenosyl-L-methionine) is essential for the AHL autoinducer biosynthesis, which involves an acylated acyl carrier protein from the fatty acid biosynthesis pathway [34]. In this regard, we consider the SAM/SPASM up-regulation as a rational act promoting the AHL biosynthesis during QS development.

In the ORF U6115_14430–U6115_14455 cluster, only one gene was expressed above the cutoff level, while the six flanking genes showed 2.0 < log2FC < 4.0 values. The most significantly up-regulated gene (ORF U6115_14435) encoded CoA-acylating methylmalonate-semialdehyde dehydrogenase, which belongs to the CoA-dependent aldehyde dehydrogenase subfamily and catalyzes the NAD-dependent oxidation of methylmalonate semialdehyde to propionyl-CoA [35]. In turn, other co-expressed genes are also involved in carbohydrate metabolism, i.e., the *mmsB* gene (ORF U6115_14455), encoding 3-hydroxyisobutyrate dehydrogenase, which catalyzes the NAD+- or NADP+-dependent oxidation of various β-hydroxyacid substrates into their cognate semialdehydes for diverse metabolic pathways [36].

The ORF U6115_14725–U6115_14770 up-regulated cluster consists of ten genes, including four characterized by log2FC > 4.0. This operon function remains unclear, since most of its genes (6 out of 10) encode hypothetical or DUF proteins. Among the genes expressed above the cutoff level, ORF 6115_14725 was found, encoding a HU family DNA-binding protein, which is a transcription regulator responsible for many important cellular processes [37]. Three other characterized products are the helix-turn-helix domain-containing protein (ORF U6115_14765), which are also involved in the regulation of gene expression [38]; the DDE-type integrase/transposase/recombinase (ORF U6115_14755), important for DNA rearrangements [39], and AAA family ATPase (ORF U6115_14750), which exert their activity through the energy-dependent remodeling or translocation of macromolecules [40].

The ORF U6115_14865 U6115_14905 cluster consists of nine genes, five of which are characterized by log2FC > 4.0. Among this operon encoded products, the capsid cement protein (ORF U6115_14880), Gp37 family protein (ORF U6115_14890), phage tail sheath subtilisin-like domain-containing protein (ORF U6115_14900), and phage major tail tube protein (ORF U6115_14905) were found, that showed this operon as one of the prophages previously discovered in *Chromobacteria* genomes [41].

In the ORF U6115_15180–U6115_15195 cluster, only one gene encoding a protein of unknown function (DUF3138) was expressed above log2FC > 4.0 cutoff level. In turn, the products of other three genes were described as a polyamine ABC transporter substrate-binding protein (ORF U6115_15195) and two ABC transporter permease subunits (ORFs U6115_15185 and U6115_15190), which indicates that they are components of the polyamine transport system [42]. Accordingly, since these systems in bacteria typically consist of a periplasmic substrate-binding protein, two transmembrane proteins, and a membrane-associated ATPase, the DUF3138 protein function can be predicted as a coupling between the ABC transporter and the ATPase. In the current context, it also appears important that the polyamine transport (via c-di-GMP biosynthesis) controls the biofilm morphogenesis and increases the biofilm biomass [43].

In addition to the gene sets, seven highly differentially expressed single genes were found.

ORF U6115_03985 encodes a nuclear transport factor 2 family protein that plays a potential non-catalytic role in ligand binding. According to current knowledge [44], this may regulate the activities of domains with which they are combined in the same polypeptide or via operonic linkage (e.g., serine/threonine protein kinases) or nucleic acid-binding domains (e.g., Zn-ribbons).

ORF U6115_04155 encodes a DUF1842 domain-containing protein, which is similar to this product of the ORF U6115_13245–U6115_13255 gene cluster (see above) and is related to the predicted SAM/SPASM maturase system XYE.

The ORF U6115_05110 product is annotated as a porin [45] that mediates the outer membrane permeability for compounds transported by the QS-regulated influx and efflux systems described above.

The ORF U6115_16120 product is a fucose-binding lectin II, which has previously been described as an adhesin of plant and animal pathogens that use protein–carbohydrate interactions for host recognition, attachment, and invasion [46].

The encoded products of three more ORFs (U6115_06250, U6115_09710, and U6115_10635) have been described as hypothetical proteins whose function in *C. subtsugae* ATCC 31532 remains unclear.

### 3.2. Putative CsuR Receptor Binding Sites in C. subtsugae ATCC 31532 Genome

*Chromobacterium* spp. are generally considered to have similar QS systems consisting of CviI/CsuI proteins, which synthesize the AHL autoinducer, and CviR/CsuR proteins, which are a cytoplasmic DNA-binding transcription factor that activates gene expression upon AHL binding [47]. In Stauff and Bassler’s study [12], the CviR binding site in *C. violaceum* ATCC 12472 strain was evaluated as a 16-base-pair palindrome with the ideal sequence CTGNCCNNNNGGNCAG, and it was suggested that this QS regulatory mechanism has a conserved feature in the *Chromobacterium* genus. In this study, we test this hypothesis in a reclassified *C. subtsugae* ATCC 31532 strain, using differentially expressed genes predicted to be under QS regulation as an example.

**Figure 3:**
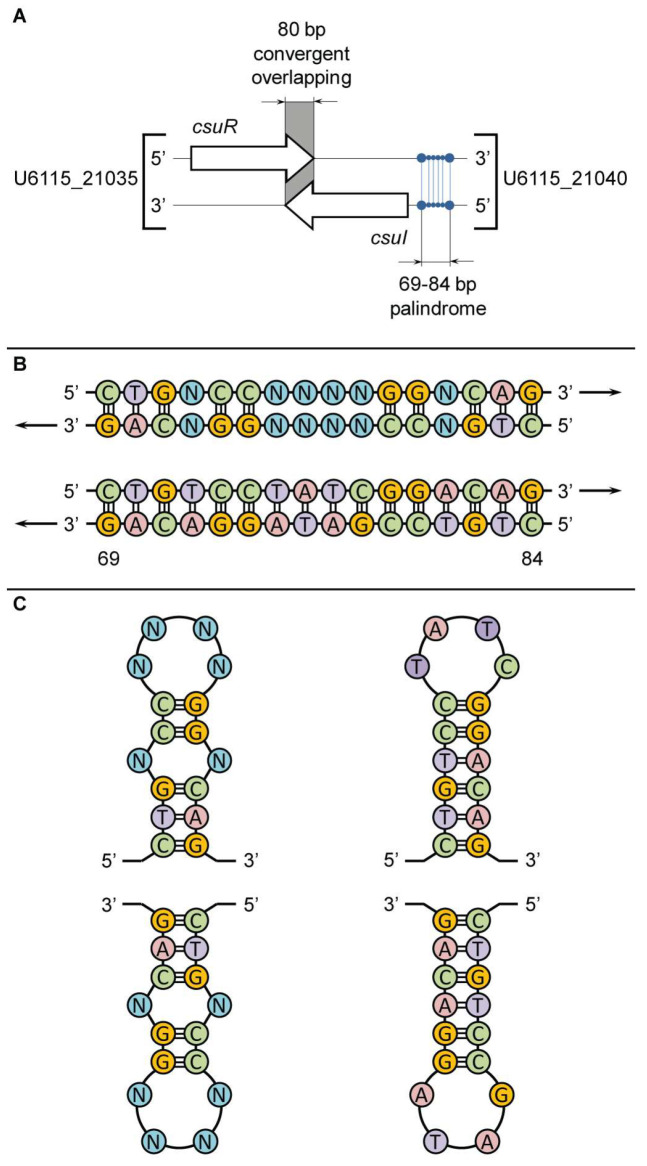
Characteristics of a 16 bp palindrome in promotor region of the *csuI* gene recognized by the CsuR transcription factor in *C. subtsugae* ATCC 31532. Panel (**A**) shows the location of the 16 bp palindrome relative to *csuR−csuI* genes; panel (**B**) shows comparison of the ideal linear sequences for the CvR binding site in *C. violaceum* ATCC 12472 (top) and the CsuR binding site in *C. subtsugae* ATCC 31532 (bottom); panel (**C**) shows the secondary stem-loop-like structural elements of the 16 bp palindromes in *C. violaceum* ATCC 12472 (left) and C. *subtsugae* ATCC 31532 (right).

In the first round of genomic analysis, we clarified the nucleotide sequence of the 16-base-pair palindrome in the promoter region of CsuI AHL-synthase (ORF U6115_21040). As in *C. violaceum* ATCC 12472, in *C. subtsugae* ATCC 31532, this gene is located on the reverse DNA strand and is transcribed in the opposite direction to the CsuR receptor gene (ORF U6115_21035), and the terminal regions of the *csuR* and *csuI* genes have an 80 bp overlap zone (Figure 3A). In the promoter zone of the *csuI* gene (positions -69 and -84 to the transcription start site), a palindrome 5′-CTGTCCGATAGGACAG-3′ was found, exactly corresponding to the previously described ideal sequence. Due to the location on the reverse strand, the GAC triplet is proximal to the *csuI* gene and the GTC triplet is distal (Figure 3B). Interestingly, the nucleotide sequences of the two paired antiparallel DNA strands indicate the possibility of developing symmetrical stem-loop-like secondary structural elements via intramolecular base pairing in the left and right single-stranded halves of the 16-base-pair palindrome (Figure 3C). This is proposed to provide a double recognition site for the dimeric CsuR protein and generates a canonical QS positive feedback regulatory loop. In turn, the overlapping topology of the convergent *csuR* and *csuI* genes may result in negative regulation of their co-expression [48], which protects the autoinduction system from “overheating”.

Accordingly, in a second round of genomic analysis, we scanned the *C. subtsugae* ATCC 31532 genome for potential CsuR binding sites that up-regulate the transcription of the genes and gene clusters described above. For this purpose, the position of the genes of interest, the direction of their transcription, and the location and size of the putative promoter regions were determined.

**Figure 4:**
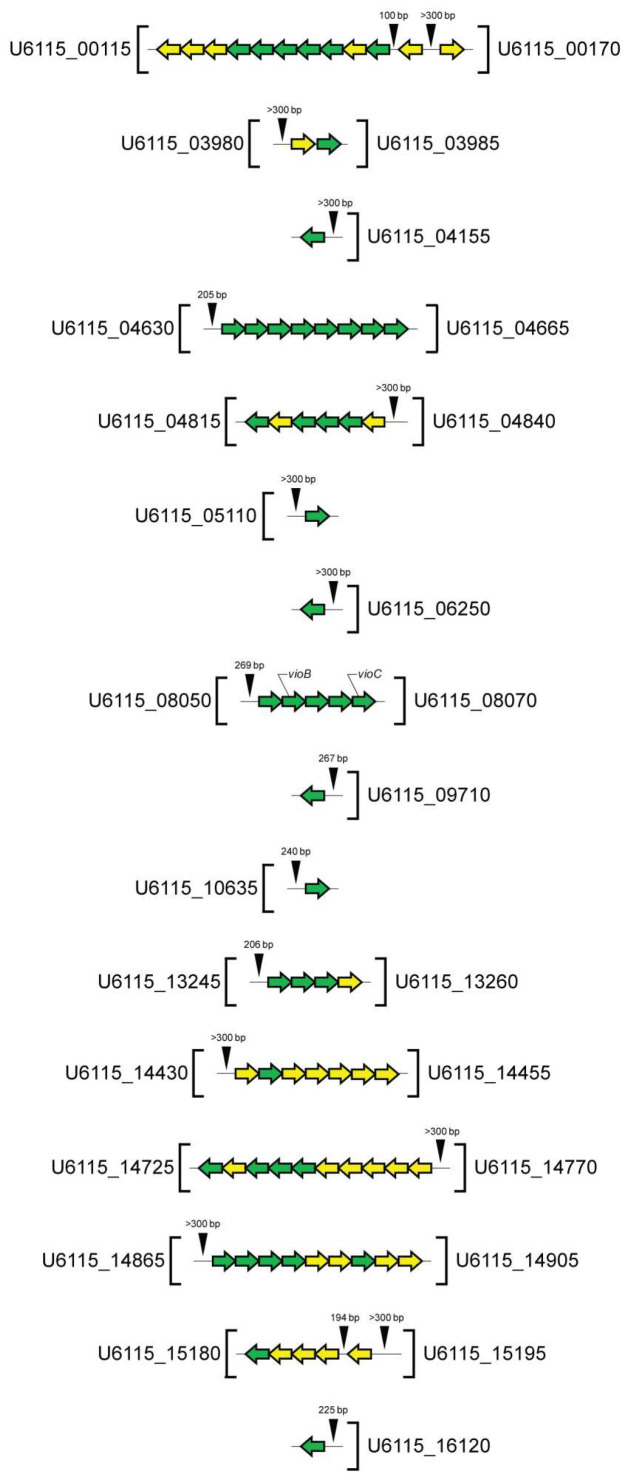
Putative promoter regions selected for screening of 16-base-pair palindromes (CsuR binding sites) involved in QS regulation in *C. subtsugae* ATCC 31532. The promotor regions’ location and size (bp) are indicated by black triangles. The transcription direction of QS-regulated genes is shown by arrows (on the forward strand, from left to right; on the reverse strand, from right to left). Genes with Log2FC > 4.0 are marked in green; those with Log2FC > 2.0 are marked in yellow.

In selecting promoter regions, we took into account the known data on prokaryotic promoters, which are typically short sequences located upstream of a target gene or operon and are commonly found within ~200 bp upstream of coding sequences [49]. Other data taken into account were the binding of bacterial RNA polymerases to DNA within a 100 bp region stretching from about 70 bp before the transcription start site to about 30 bp after it [50]. According to these criteria, DNA regions located upstream of the transcription initiation site, a lack of overlap with coding sequences, and having a size of at least 100 bp and up to 200 bp were selected. If the intergenic region was longer, the first 200 bp were taken into account in the analysis. Using this approach, we identified 18 genomic regions residing in the intergenic or promoter zones, as shown in Figure 4.

The sequences of the selected genomic regions were extracted (see Supplement S2) and screened for the presence of CsuR binding sites. Since the genes of interest are transcribed from both forward and reverse DNA strands and the LuxR family transcriptional activators function as a homodimer in an ambidextrous manner [51], the 16-base-pair palindrome scanning was performed for four variants: (i) CTGTCCTATCGGACAG—forward motif; (ii) GACAGGATAGCCTGTC—reverse motif (found in *csuI* gene promoter zone); (iii) GACAGGCTATCCTGTC—inverted forward motif; and (iv) CTGTCCGATAGGACAG—inverted reverse motif. Given that the bacterial RNA polymerase binding site requires approximately 70 bp upstream of the transcription start site (see above), potential CsuR binding sites found closer than 70 bp upstream of the transcription start sites were rejected. The identified DNA sequences, their similarity to the ideal sequence required for CsuR recognition, and their position in the analyzed promoter regions are presented in Table 2.

In total, we identified 28 potential CsuR binding sites that showed >50% similarity to various ideal sequence motifs. In several promotor regions, more than one 16-base-pair sequence were observed, while upstream of U6115_RS14430 and U6115_RS14770 these sequences were not found.

On the forward DNA strand in the *vio*-operon promoter zone (positions −75 and −88 with respect to the *vioA* gene transcription start site), the 5′-AAGAGCTGAGCCATTC-3′ sequence was found. Importantly, this 16-base-pair region showed only 56.25% similarity with the ideal sequence, indicating a low affinity for CsuR and explaining why the level of violacein production in *C. subtsugae* ATCC 31532 strain is decreased compared to the *C. violaceum* ATCC 12472 strain [5], which has a more correct palindromic site required for the *vio*-operon promoter activation [12]. Another 16-base-pair sequence within the 200 bp analyzed region also had 56.25% similarity to the ideal sequence, but its position (-135 and -150 with respect to the *vioA* gene transcription start site) made it less likely to be a site for transcriptional activation of the *vio* operon.

When compared with Koirala et al.’s preprint analyzing the promoter regions of the QS-controlled *cdeAB*-*oprM* genes [26] based on the available whole genome data [22], we found that these genes are transcribed from the forward DNA strand in the same direction as the MarR regulator, and based on the transcriptomic data, they were shown to be co-expressed with four other genes located upstream of the *marR* gene. Accordingly, a 209 pb region upstream of ORF U6115_04630 was established as a putative promoter region for this operon, where the 5′-GAGAAAATATCCTATG-3′ sequence (positions −101 and −116 to the transcription start site; 62.5% ideal sequence similarity) was found. Another 16-base-pair sequence was located somewhat further from the transcription start site (positions −114 and −129) and showed less similarity to the ideal sequence (56.25%).

Other putative CsuR binding sites were described for the first time and require confirmation of their involvement in QS regulation using experimental genetics methods.

## 4. Discussion

The study results show that the QS system in the *C. subtsugae* ATCC 31532 strain is significantly different from the canonical signaling circuits [4] and is also not identical to the closely related *C. violaceum* ATCC 12472 strain [12].

In contrast to the canonical LuxI-LuxR system in *Vibrio fischeri* [52], also using acylated homoserine lactones for cell-to-cell communication, the homologous system in *C. subtsugae* ATCC 31532 exhibits the following features: (i) the genes encoding the AHL signal (*csuI*) and the AHL-receptor (*csuR*) are located separately from the QS-controlled genes (in *V. fischeri*, the *luxI/luxR* genes are spatially combined and co-expressed with the directly regulated *luxCDABEG* operon); (ii) the *csuI* and *csuR* genes are transcribed convergently with each other and have an 80 bp partial overlap zone (in *V. fischeri*, the luxI/luxR genes are transcribed divergently). Apparently, such QS genetic control, as well as similar 16-base-pair palindrome in the promoter region of the AHL-synthase genes, is typical of the *Chromobacteria* genus [12], which provides negative and positive feedback regulatory loops in the autoinduction process. As a result, in the current transcriptomic analysis, we observed only a moderate increase in the transcription level of the *csuI-csuR* genes during the QS development.

**Figure 5:**
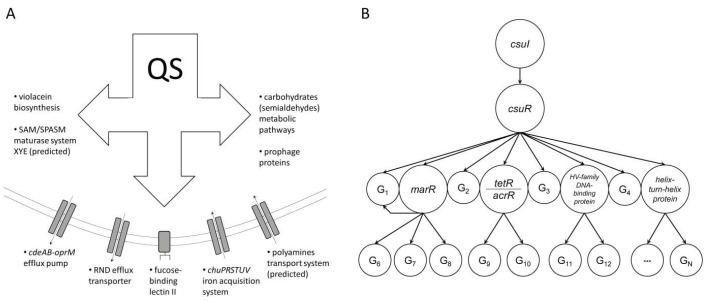
QS-controlled traits (**A**) and hypothetical hierarchical QS regulatory network (**B**) in *C. subtsugae* ATCC 31532 (description in the text).

Comparison of the QS-controlled genes in *C. subtsugae* ATCC 31532 and *C. violaceum* ATCC 12472 revealed significantly different transcriptome profiles, with only the production of violacein, which is toxic to eukaryotic cells by triggering mitochondrial membrane hyperpolarization [53], being common. Against the previously described up-regulation of chitinase, type VI secretion-related genes, transcriptional regulator gene, and guanine deaminase gene in *C. violaceum* ATCC 12472 [12], the QS-controlled traits in *C. subtsugae* ATCC 31532 included the following: (i) *cdeAB-oprM* efflux pump; (ii) RND efflux transporter; (iii) *chuPRSTUV* iron acquisition system; (iv) polyamine transport system; (v) carbohydrate (semialdehydes) metabolic pathways; (vi) SAM/SPASM maturase system XYE (predicted); (vii) prophage proteins; and (viii) fucose-binding lectin II (Figure 5A). In essence, we found evolutionary divergence in the ecological strategies of the compared species, which allows them to avoid competition with each other and gives an advantage in specific ecological niches.

It seems important that several up-regulated genes and operons in *C. subtsugae* ATCC 31532 appear to be involved in biofilm development (polyamine transport system, RND efflux transporter), competition (iron acquisition system), or virulence (fucose-binding lectin II). However, further studies are needed to fully assess the significance of QS in these traits, which implies gene ontology enrichment or/and pathway analysis, where additional tools such as the Database for Annotation, Visualization, and Integrated Discovery (DAVID) [54] may be useful for understanding the biological meaning behind the comprehensive lists of QS-regulated genes.

Overall, the obtained transcriptomic data revealed distinct QS-controlled lifestyles for *C. violaceum* and *C. subtsugae*, the first being considered as free-living soil bacterium and animal/human emergent pathogen [34], while the second is considered as an environmental organism, pathogenic for insects [16].

Notably, when comparing the list of QS-controlled genes, chitinase is found among the up-regulated traits in *C. violaceum* ATCC 12472, while it is absent in *C. subtsugae* ATCC 31532. This observation makes questionable the chitinase assay in QS modulation experiments using the ATCC 31532-derived CV026 biosensor strain [55] and requires the search for new validation tests. In turn, this study shows that fucose-binding lectin II is the most attractive candidate for such a test, which is directly controlled by QS and can be quantified [56].

The obtained data also allow us to formulate a general concept about the mechanisms of QS control in the *C. subtsugae* ATCC 31532 strain. In contrast to the various regulatory systems described during thirty years of QS studies in bacteria (the hierarchical cascade in *Vibrio harveyi* [57], the multilayered network in *Pseudomonas aeruginosa* [58], etc.) most of which use more than one autoinducer and AI-driven transcriptional factor, *C. subtsugae* exhibits the simplest QS system driven by a single AHL signal and involving one CsuR regulatory protein.

The current results generally support this concept, as the screening of the promoter regions of the up-regulated genes and operons in most cases revealed potential CsuR binding sites with 56.25–68.75% similarity to the ideal 16-base-pair palindrome found upstream of the *csuI* gene. However, this mechanism explains the transcription of a minority of 261 up-regulated genes, which requires the involvement of additional intracellular regulators for QS signal dissemination.

In this context, it is important that a number of genes encoding several transcriptional regulators are under direct QS control. Among them, MarR is described as a member of the winged-helix-turn-helix family of transcription factors, which are critical for the response of bacterial cells to chemical signals and for the conversion of such signals into changes in gene activity [59]; a transcriptional regulator of the TetR/AcrR family also involved in efflux regulation and biofilm formation [60]; a HU family DNA-binding protein responsible for bacteria survival, SOS response, and virulence gene expression, [37]; and a helix-turn-helix domain-containing protein [38]. These data suggest an original QS regulatory network design in *C. subtsugae* ATCC 31532, whose hierarchical architecture includes the direct control of target genes via canonical autoinduction mechanism and further dissemination of the effect through QS-controlled transcriptional regulators activity (Figure 5B).

Among these regulators, the most interesting is MarR, which is highly co-expressed with several upstream and downstream genes and is predicted to participate in a positive feedback regulatory loop that enhances the direct action of QS. This hypothesis requires further experimental testing, where the preferred approach is to delete the *marR* gene, which will clearly show the degreeof its involvement.

Several intriguing facts remain unexplained after the current analysis. First, the products of numerous genes shown to be QS-regulated were characterized as hypothetical or DUF proteins. Some of them have been predicted to be involved in factor A biosynthesis or the SAM/SPASM maturase system, or to be putative prophage proteins, but most of them require further in-depth study. A second unexpected finding was the up-regulation of a gene encoding an AfsA-related hotdog domain-containing protein, which is a key enzyme in A-factor (2-isocapryloyl-3R-hydroxymethyl-γ-butyrolactone) biosynthesis [27], as well as three flanking genes that are also presumed to be involved in this microbial hormone biosynthetic pathway. Currently, the same γ-butyrolactone gene cluster homologues have been revealed across 12 bacterial phyla [61], while such biosynesthetic activity in *C. subtsugae* has not been reported either. An explanation of this phenomenon will allow a better understanding of interspecies cross-talk in the *C. subtsugae* environmental community, since their implication for bacterial communication is still unknown.

## 5. Conclusions

Transcriptomic and genomic analyses have provided new insights into the lifestyle of the *C. subtsugae* ATCC 31532 strain, which coordinates gene expression according to population density via a hierarchical QS regulatory mechanism. Its comparison with the well-studied *C. violaceum* ATCC 12472 strain revealed significantly different QS-controlled transcriptomic profiles shared only in the pigment violacein production, indicating evolutionary divergence in the ecological strategies of the represented species. Thus, the obtained data support the concept of *C. subtsugae* ATCC 31532 as a wild-type strain utilizing QS for pathogenicity against insects and also propose new features for assessing quorum quenching using the derived biosensor strain CV026.

## Figures and Tables

**Table 1:** List of genes predicted to be under QS regulation in *C. subtsugae* ATCC 31532 strain through RNA-seq dataset analyses.

ORF No.	Product Description	9 h (QS−) Sample Expression	18 h (QS+) Sample Expression	Log2 (Fold Change)
…	…			
U6115_00130	acyltransferase	265.58	6104.15	4.5223
U6115_00135	flavin reductase family protein	222.29	3654.83	4.038
U6115_00140	SDR family oxidoreductase	308.96	14,269.74	5.5261
U6115_00145	MFS transporter	352.10	12,816.07	5.1849
U6115_00150	cupin domain-containing protein	168.27	5722.99	5.0879
…	…			
U6115_00160	FAD-binding oxidoreductase	239.46	9032.96	5.2337
…	…			
…	…			
U6115_03985	nuclear transport factor 2 family protein	426.80	10,029.22	4.5549
U6115_04155	DUF1842 domain-containing protein	3578.32	76,174.05	4.4121
U6115_04630	hypothetical protein	463.24	90,275.91	7.6056
U6115_04635	AfsA-related hotdog domain-containing protein	227.75	111,013.08	8.9269
U6115_04640	HAD-IB family hydrolase	80.45	46,715.15	9.1777
U6115_04645	DUF2165 family protein	81.60	29,104.70	8.4833
U6115_04650	MarR family transcriptional regulator	82.29	27,891.04	8.3998
U6115_04655	efflux transporter outer membrane subunit	169.53	51,325.66	8.2401
U6115_04660	HlyD family efflux transporter periplasmic adaptor subunit	241.02	78,278.93	8.344
U6115_04665	DHA2 family efflux MFS transporter permease subunit	505.29	75,629.69	7.2257
U6115_04815	ABC transporter ATP-binding protein	739.58	17,230.47	4.5431
…	…			
U6115_04825	ABC transporter substrate-binding protein	732.78	12,679.79	4.1139
U6115_04830	hemin-degrading factor	2186.39	40,476.44	4.211
U6115_04835	TonB-dependent hemoglobin/transferrin/lactoferrin family receptor	4640.67	75,458.91	4.0235
…	…			
U6115_05110	porin	5085.22	86,187.67	4.0832
U6115_06250	hypothetical protein	4504.26	84,702.20	4.2331
U6115_08050	FAD-dependent oxidoreductase	463.89	7559.13	4.0287
U6115_08055	iminophenyl-pyruvate dimer synthase VioB	522.94	8812.35	4.0771
U6115_08060	NAD(P)/FAD-dependent oxidoreductase	505.23	19,786.24	5.293
U6115_08065	tryptophan hydroxylase	739.89	29,065.83	5.2973
U6115_08070	violacein biosynthesis enzyme VioE	424.25	15,023.60	5.1455
U6115_09710	hypothetical protein	1044.89	17,971.71	4.1047
U6115_10635	hypothetical protein	1021.42	19,171.38	4.2317
U6115_13245	DUF1843 domain-containing protein	78.56	2425.74	4.9472
U6115_13250	DUF1842 domain-containing protein	318.76	11,303.45	5.148
U6115_13255	DUF1842 domain-containing protein	247.45	9744.09	5.2992
…	…			
…	…			
U6115_14435	CoA-acylating methylmalonate-semialdehyde dehydrogenase	3465.35	63,003.03	4.1846
…	…			
U6115_14725	HU family DNA-binding protein	199.18	3597.91	4.1771
…	…			
U6115_14735	hypothetical protein	77.15	1750.40	4.5112
U6115_14740	hypothetical protein	63.99	1474.44	4.5138
U6115_14745	hypothetical protein	35.72	1204.11	5.0607
…	…			
U6115_14865	peptidase	264.97	4716.73	4.1546
U6115_14870	hypothetical protein	193.55	6526.27	5.0729
U6115_14875	hypothetical protein	23.64	717.12	4.9079
U6115_14880	capsid cement protein	21.49	503.90	4.5344
…	…			
U6115_14895	hypothetical protein	62.70	1760.16	4.8166
…	…			
U6115_15180	DUF3138 family protein	638.68	26,856.19	5.3953
…	…			
U6115_16120	fucose-binding lectin II	1206.95	64,464.05	5.7393

(…) shows the presence of co-expressed genes with 2.0 < log2FC < 4.0 values.

**Table 2:** List of 16-base-pair sequences in promoter regions of *C. subtsugae* ATCC 31532 genes predicted as QS regulated according to transcriptome analysis data.

ORF Promoter	DNA Strand	Sequence Variant for Alignment	16-Base-Pair Sequence Found (Matching Nucleotides Are Underlined)	Position in Relation to the Transcription Start Site (−bp)	Ideal Sequence Similarity (%)
U6115_RS00160	reverse	inverted reverse motif	TGA**TCCG**C**TA**C**G**G**C**G**G**	79–94	56.25
U6115_RS00165	reverse	reverse motif	**GA**G**AGG**GC**AG**AGG**GT**G	135–150	56.25
U6115_RS00170	forward	inverted forward motif	**G**CT**AG**A**C**A**A**G**C**A**TG**G**C**	107–122	56.25
		reverse motif	CGG**A**C**GA**A**AGCC**G**G**C**C**	126–141	56.25
U6115_RS03980	forward	forward motif	**C**A**GTCC**G**ATC**AT**A**A**A**T	88–103	62.50
		reverse motif	**GACAGG**C**T**GC**C**C**T**GT**C**	146–161	62.50
U6115_RS04155	reverse	inverted forward motif	**G**G**C**G**G**TAA**ATCC**G**GTC**	127–142	62.50
U6115_RS04630	forward	reverse motif	**GA**G**A**AA**ATA**T**CCT**A**T**G	101–116	62.50
		inverted reverse motif	**CT**AAAT**GATAG**CCG**AG**	114–129	56.25
U6115_RS04840	reverse	inverted forward motif	**G**C**CAGG**G**T**CCA**C**C**GT**T	81–96	56.25
U6115_RS05110	forward	reverse motif	**G**G**C**G**G**A**ATA**TA**C**C**GTC**	77–92	62.50
U6115_RS06250	reverse	reverse motif	**G**CT**A**TTT**TAGC**T**T**T**TC**	120–135	56.25
U6115_RS08050	forward	reverse motif	A**A**G**AG**CTG**AGCC**AT**TC**	73–88	56.25
		inverted reverse motif	**C**C**G**C**CCG**CCG**GGA**A**A**C	135–150	56.25
U6115_RS09710	reverse	inverted reverse motif	**CTG**A**CCG**T**TA**A**GA**AC**G**	91–106	68.75
U6115_RS10635	forward	inverted reverse motif	**C**AT**TCC**AT**T**TC**G**T**CAG**	135–150	56.25
		inverted forward motif	**G**T**CA**TT**C**C**AT**TTC**GTC**	137–152	56.25
		forward motif	**CTG**GG**CT**GCG**GG**CG**AG**	169–184	56.25
		reverse motif	**G**TTT**GG**T**T**T**GCCTG**GG	180–195	56.25
U6115_RS13245	forward	reverse motif	**GA**A**AG**C**A**CT**GCC**C**G**G**C**	73–88	62.50
		forward motif	**CTGT**A**CT**TAA**GG**G**CAG**	116–131	68.75
		inverted forward motif	CC**CAG**C**C**C**A**AG**CTG**A**C**	177–192	56.25
U6115_RS14430	forward	not found			
U6115_RS14770	reverse	not found			
U6115_RS14865	forward	forward motif	**C**AC**TC**A**T**C**TC**TT**ACA**T	121–136	56.25
U6115_RS15190	reverse	reverse motif	**G**C**C**G**GG**CCC**GCCTGT**T	80–95	62.50
		inverted reverse motif	**C**A**G**G**CCG**GC**AGG**G**C**C**G**	108–123	62.50
		forward motif	**CTGT**T**CT**GG**C**CTG**C**C**G**	158–173	56.25
U6115_RS15195	reverse	reverse motif	CG**C**GC**G**T**TAGCC**C**GTC**	177–192	62.50
U6115_RS16120	reverse	inverted reverse motif	**C**C**GTCCG**T**T**T**GGA**GCC	170–185	62.50

## Data Availability

The original contributions presented in this study are included in the article/Supplementary Materials. Further inquiries can be directed to the corresponding authors.

## Supplementary Materials

The following supporting information can be downloaded at: https://www.mdpi.com/article/10.3390/microorganisms13051021/s1, Supplement S1: Whole transcriptome dataset of *C. subtsugae* ATCC 31532 strain; Supplement S2: DNA sequences of promoter/intergenic regions located upstream of QS-controlled genes in *C. subtsugae* ATCC 31532 strain.
