# Supplementary material for: Quorum Sensing in Chromobacterium subtsugae ATCC 31532 (Formerly Chromobacterium violaceum ATCC 31532): Transcriptomic and Genomic Analyses
Source: Microorganisms. 2025 Apr 29;13(5):1021. doi: 10.3390/microorganisms13051021 (PMC12114271; doi:10.3390/microorganisms13051021)
Supplement: Supplementary file 1 [file microorganisms-13-01021-s001.zip › Supplement_S1.pdf]

Quorum Sensing in *Chromobacterium subtsugae* ATCC 31532 (formerly - *Chromobacterium violaceum* ATCC 31532): Transcriptomic and Genomic Analyses

Supplement S1. Whole transcriptome dataset of *C. subtsugae* ATCC 31532 strain

ORF.no.	Gene name	Product description	Exponential sample expression	"Quorum Sensing" sample expression	Log2 (fold change)	P values
U6115_00005	dnaA	chromosomal replication initiator protein DnaA	24671.99	12526.64	-0.9779	4.3E-21
U6115_00010	dnaN	DNA polymerase III subunit beta	29758.68	31154.59	0.0661	5.8E-01
U6115_00015	gyrB	DNA topoisomerase (ATP-hydrolyzing) subunit B	67716.58	46530.14	-0.5414	1.6E-05
U6115_00020		restriction endonuclease	19263.32	9733.78	-0.9848	3.0E-04
U6115_00025		hypothetical protein	49478.54	24612.85	-1.0074	1.1E-05
U6115_00030		hypothetical protein	18828.71	9135.98	-1.0433	1.7E-04
U6115_00035	dndE	DNA sulfur modification protein DndE	2565.52	1185.52	-1.1138	2.9E-04
U6115_00040	dndD	DNA sulfur modification protein DndD	13468.38	5565.64	-1.275	1.6E-16
U6115_00045	dndC	DNA phosphorothioation system sulfurtransferase DndC	10862.90	3767.32	-1.5279	6.2E-30
U6115_00050	dndB	DNA sulfur modification protein DndB	9627.07	3090.27	-1.6394	5.9E-32
U6115_00055		hypothetical protein	8938.76	3231.26	-1.468	1.5E-13
U6115_00060		DUF1484 family protein	1553.94	3242.13	1.0622	1.3E-10
U6115_00065		DUF1484 family protein	126.40	325.66	1.3561	5.1E-14
U6115_00070		DUF1484 family protein	157.00	381.62	1.2797	8.1E-09
U6115_00075		hypothetical protein	1954.85	982.56	-0.9921	4.6E-10
U6115_00080		hypothetical protein	1626.17	661.61	-1.2973	4.2E-14
U6115_00085		hypothetical protein	2632.89	1621.26	-0.6995	4.0E-04
U6115_00090		hypothetical protein	2280.72	1588.72	-0.5213	1.0E-02
U6115_00095		hypothetical protein	3595.75	2065.07	-0.7999	1.2E-04
U6115_00100		type VI secretion system Vgr family protein	13120.90	13395.76	0.03	8.2E-01
U6115_00105		serine hydrolase domain-containing protein	2324.49	10337.09	2.1525	1.1E-30
U6115_00110		LysR family transcriptional regulator	1611.70	3118.97	0.9523	1.1E-09
U6115_00115		efflux transporter outer membrane subunit	645.59	4224.37	2.7085	8.4E-57
U6115_00120		multidrug efflux RND transporter permease subunit	1343.62	9769.10	2.8617	5.7E-45
U6115_00125		efflux RND transporter periplasmic adaptor subunit	349.14	4248.20	3.6033	6.2E-65
U6115_00130		acyltransferase	265.58	6104.15	4.5223	1.6E-81
U6115_00135		flavin reductase family protein	222.29	3654.83	4.038	3.9E-52
U6115_00140		SDR family oxidoreductase	308.96	14269.74	5.5261	7.8E-117

U6115_00145		MFS transporter	352.10	12816.07	5.1849	1.5E-153
U6115_00150		cupin domain-containing protein	168.27	5722.99	5.0879	6.5E-117
U6115_00155		acyl-CoA dehydrogenase family protein	1725.89	15198.27	3.1384	4.9E-67
U6115_00160		FAD-binding oxidoreductase	239.46	9032.96	5.2337	9.5E-124
U6115_00165		GNAT family N-acetyltransferase	463.50	7136.79	3.9441	1.4E-73
U6115_00170		TetR/AcrR family transcriptional regulator	1965.06	23975.73	3.6087	5.3E-148
U6115_00175		MBL fold metallo-hydrolase	2200.07	11639.56	2.4034	2.2E-113
U6115_00180		LysR family transcriptional regulator	1884.86	3708.65	0.9757	2.9E-16
U6115_00185		methyl-accepting chemotaxis protein	5934.93	6212.52	0.0659	5.8E-01
U6115_00190		hypothetical protein	101.37	133.07	0.3874	8.6E-02
U6115_00195		ATP-grasp domain-containing protein	568.47	967.31	0.7655	2.4E-05
U6115_00200		MFS transporter	868.61	1265.50	0.5413	3.9E-05
U6115_00205		hypothetical protein	479.53	833.59	0.797	1.2E-08
U6115_00210		ATP-grasp domain-containing protein	408.59	685.62	0.7453	1.2E-06
U6115_00215		hypothetical protein	149.39	230.14	0.6225	2.4E-03
U6115_00220		1-deoxy-D-xylulose-5-phosphate synthase N-terminal domain-containing protein	139.51	194.80	0.475	1.2E-02
U6115_00225		M20/M25/M40 family metallo-hydrolase	331.09	458.82	0.4734	3.2E-03
U6115_00230	argC	N-acetyl-gamma-glutamyl-phosphate reductase	196.85	244.68	0.3123	1.3E-01
U6115_00235		DegT/DnrJ/EryC1/StrS family aminotransferase	426.02	497.85	0.2216	2.1E-01
U6115_00240		hypothetical protein	154.20	178.25	0.2095	3.1E-01
U6115_00245		RimK family alpha-L-glutamate ligase	325.24	273.20	-0.2495	1.4E-01
U6115_00250		lysine biosynthesis protein LysW	261.59	120.25	-1.1187	1.1E-05
U6115_00255		NAD-dependent epimerase/dehydratase family protein	407.63	670.23	0.7168	1.7E-07
U6115_00260		SDR family oxidoreductase	532.28	828.87	0.64	1.5E-04
U6115_00265		antibiotic biosynthesis monooxygenase	291.87	363.24	0.3162	1.0E-01
U6115_00270		LysR substrate-binding domain-containing protein	1668.24	2403.41	0.5271	7.7E-03
U6115_00275		peptidase domain-containing ABC transporter	1629.84	2638.35	0.6957	2.3E-06
U6115_00280		HlyD family efflux transporter periplasmic adaptor subunit	1325.98	1978.48	0.578	1.9E-04
U6115_00285		hypothetical protein	3203.19	50931.53	3.9914	5.0E-143
U6115_00290		hypothetical protein	2536.36	9145.98	1.8505	4.1E-21
U6115_00295		asparagine synthase-related protein	1833.32	6727.15	1.8757	3.0E-20

U6115_00300		transketolase	648.15	2877.21	2.1502	1.1E-24
U6115_00305		transketolase C-terminal domain-containing protein	950.01	4475.86	2.2362	3.9E-27
U6115_00310		glycosyltransferase family 2 protein	788.75	4473.06	2.5038	2.4E-33
U6115_00315		glycosyltransferase	757.34	3124.22	2.0447	1.6E-25
U6115_00320		acyltransferase	1064.47	3476.85	1.708	1.6E-24
U6115_00325		hypothetical protein	223.12	3001.44	3.7501	1.6E-43
U6115_00330		hypothetical protein	1515.99	2236.70	0.5612	6.8E-03
U6115_00335		sigma-54 dependent transcriptional regulator	1426.36	1632.11	0.1949	2.7E-01
U6115_00340		HAMP domain-containing sensor histidine kinase	2188.71	2431.98	0.1524	2.7E-01
U6115_00345	parC	DNA topoisomerase IV subunit A	8299.40	6770.91	-0.2937	9.7E-03
U6115_00350		GNAT family N-acetyltransferase	1564.69	2431.68	0.6367	1.0E-07
U6115_00355		TonB-dependent receptor	13640.27	16638.10	0.2867	2.6E-01
U6115_00360	hemA	glutamyl-tRNA reductase	16416.85	9762.47	-0.7499	1.6E-08
U6115_00365	prfA	peptide chain release factor 1	7679.77	5343.16	-0.5237	5.3E-06
U6115_00370		c-type cytochrome	1197.38	1335.98	0.1592	2.9E-01
U6115_00375		DUF6691 family protein	218.72	221.22	0.0182	9.5E-01
U6115_00380		YeeE/YedE family protein	198.48	209.31	0.0815	7.4E-01
U6115_00385		metalloregulator ArsR/SmtB family transcription factor	365.85	259.99	-0.4929	1.6E-02
U6115_00390		carboxymuconolactone decarboxylase family protein	594.75	704.42	0.2409	9.0E-02
U6115_00395		B12-binding domain-containing radical SAM protein	7362.81	4568.68	-0.6884	7.5E-06
U6115_00400		MBL fold metallo-hydrolase	5461.88	5149.07	-0.0848	5.5E-01
U6115_00405		MGMT family protein	1173.33	707.79	-0.7268	9.8E-06
U6115_00410		outer membrane protein OmpK	46830.84	21300.52	-1.1366	8.6E-13
U6115_00415		diguanylate cyclase	1263.11	1152.64	-0.1323	4.2E-01
U6115_00420		ribonuclease catalytic domain-containing protein	17018.28	10425.93	-0.7069	9.1E-12
U6115_00425		hypothetical protein	137.79	95.63	-0.529	3.0E-02
U6115_00430		long-chain-fatty-acid--CoA ligase	584.48	387.05	-0.5977	1.5E-03
U6115_00435		HDOD domain-containing protein	920.35	647.33	-0.5091	6.5E-03
U6115_00440		methyl-accepting chemotaxis protein	2149.08	1646.00	-0.3849	6.7E-02
U6115_00445		glycoside hydrolase family 16 protein	2287.33	1710.05	-0.4201	1.4E-02
U6115_00450		SAM-dependent methyltransferase	10505.82	9042.96	-0.2164	6.3E-02
U6115_00455		META and DUF4377 domain-containing protein	18309.47	21017.91	0.199	1.3E-01
U6115_00460		META domain-containing protein	2870.59	3255.33	0.1815	1.4E-01

U6115_00465		NAD(P)(+) transhydrogenase (Re/Si-specific) subunit beta	2179.79	2311.07	0.0837	4.9E-01
U6115_00470		proton-translocating transhydrogenase family protein	287.11	323.19	0.1689	6.0E-01
U6115_00475		Re/Si-specific NAD(P)(+) transhydrogenase subunit alpha	3492.21	3867.62	0.1475	1.8E-01
U6115_00480		transporter substrate-binding domain-containing protein	649.70	589.63	-0.1405	4.0E-01
U6115_00485		histidine kinase	552.85	563.08	0.0286	8.9E-01
U6115_00490		TMEM175 family protein	307.74	357.71	0.2189	1.7E-01
U6115_00495		DUF3460 family protein	22062.84	19143.39	-0.2048	2.2E-01
U6115_00500		class I SAM-dependent methyltransferase	6704.36	3797.50	-0.8203	3.4E-12
U6115_00505		ExbD/TolR family protein	7812.99	6906.97	-0.1781	1.6E-01
U6115_00510		energy transducer TonB	9486.58	7431.99	-0.3522	3.8E-02
U6115_00515	tolB	Tol-Pal system beta propeller repeat protein TolB	11946.59	15423.08	0.3684	1.9E-02
U6115_00520		OmpA family protein	49125.95	132745.01	1.4341	2.1E-19
U6115_00525	ybgF	tol-pal system protein YbgF	14031.98	21886.32	0.6412	8.5E-06
U6115_00530	rng	ribonuclease G	12944.64	12226.91	-0.0823	4.6E-01
U6115_00535		Maf family protein	9594.97	6162.98	-0.6386	5.5E-07
U6115_00540		Bcr/CflA family efflux MFS transporter	2976.61	1820.20	-0.7091	5.0E-07
U6115_00545	rsmA	16S rRNA (adenine(1518)-N(6)/adenine(1519)-N(6))-dimethyltransferase RsmA	9700.82	4834.03	-1.0049	3.0E-13
U6115_00550		glutamate/aspartate ABC transporter substrate-binding protein	20387.42	33953.48	0.7359	3.4E-08
U6115_00555		amino acid ABC transporter permease	2052.30	2630.79	0.359	1.2E-02
U6115_00560		ABC transporter permease subunit	1968.30	2289.43	0.2178	9.5E-02
U6115_00565		amino acid ABC transporter ATP-binding protein	5190.80	4860.54	-0.0945	4.4E-01
U6115_00570		transporter substrate-binding domain-containing protein	2086.51	2698.63	0.3722	2.2E-02
U6115_00575	argH	argininosuccinate lyase	9525.26	11189.28	0.2325	4.6E-02
U6115_00580		cyclic diguanylate phosphodiesterase	2001.98	1593.88	-0.3286	2.1E-03
U6115_00585		amino acid racemase	1032.58	855.31	-0.2719	2.4E-02
U6115_00590		AraC family transcriptional regulator	690.59	790.89	0.197	1.8E-01
U6115_00595		NAD(P)-dependent alcohol dehydrogenase	751.97	751.35	-0.003	9.8E-01
U6115_00600		DeoR family transcriptional regulator	35798.40	21544.32	-0.7326	9.1E-05
U6115_00605		BPSS1780 family membrane protein	7871.83	8606.65	0.1288	2.6E-01

U6115_00610	tkt	transketolase	48173.16	39548.98	-0.2846	3.3E-02
U6115_00615	gap	type I glyceraldehyde-3-phosphate dehydrogenase	2410.13	1249.26	-0.9492	5.9E-10
U6115_00620		hypothetical protein	19023.39	13403.94	-0.505	1.3E-04
U6115_00625		phosphoglycerate kinase	20287.17	19926.59	-0.0259	8.4E-01
U6115_00630		LysE family translocator	10647.29	9866.92	-0.1098	4.6E-01
U6115_00635	fba	class II fructose-bisphosphate aldolase	75217.62	73137.01	-0.0405	8.1E-01
U6115_00640		amino acid ABC transporter substrate-binding protein	666.42	863.53	0.3739	1.3E-02
U6115_00645	moaD	molybdopterin converting factor subunit 1	542.72	422.17	-0.3641	7.9E-03
U6115_00650	moaE	molybdopterin synthase catalytic subunit MoaE	1599.51	1935.12	0.2756	5.8E-02
U6115_00655	thpR	RNA 2',3'-cyclic phosphodiesterase	874.00	1022.61	0.2281	2.3E-01
U6115_00660		response regulator	2362.78	3501.95	0.5675	1.9E-05
U6115_00665		response regulator	3254.20	3522.99	0.1147	3.0E-01
U6115_00670		PilT/PilU family type 4a pilus ATPase	6936.79	6472.77	-0.0999	4.7E-01
U6115_00675		type IV pilus twitching motility protein PilT	7603.86	5403.45	-0.4928	4.1E-04
U6115_00680		YggS family pyridoxal phosphate-dependent enzyme	6806.40	7037.91	0.0485	7.2E-01
U6115_00685	proC	pyrroline-5-carboxylate reductase	4707.69	6108.48	0.3759	1.5E-03
U6115_00690		YggT family protein	4924.83	5307.77	0.1079	3.8E-01
U6115_00695		c-type cytochrome	10514.68	12750.73	0.2783	1.1E-01
U6115_00700	dksA	RNA polymerase-binding protein DksA	49111.45	43517.94	-0.1744	2.8E-01
U6115_00705	aroG	3-deoxy-7-phosphoheptulonate synthase AroG	18189.15	14842.44	-0.2934	6.8E-03
U6115_00710		PHB depolymerase family esterase	7794.18	28166.61	1.8537	4.2E-35
U6115_00715		carboxymuconolactone decarboxylase family protein	267.96	234.16	-0.1994	3.0E-01
U6115_00720		LysR family transcriptional regulator	622.16	594.19	-0.0671	6.7E-01
U6115_00725		deoxyribonuclease II family protein	648.34	753.62	0.2153	2.3E-01
U6115_00730		class I SAM-dependent methyltransferase	2338.43	3271.68	0.4843	2.1E-05
U6115_00735		NADPH-dependent 2,4-dienoyl-CoA reductase	3707.98	4239.05	0.1931	1.4E-01
U6115_00740		carbonic anhydrase	1640.15	4824.09	1.5563	1.5E-24
U6115_00745		glutathione S-transferase	2670.67	5285.25	0.9849	2.9E-16
U6115_00750		alpha/beta hydrolase	1360.37	2517.55	0.8879	3.8E-10
U6115_00755		GNAT family N-acetyltransferase	3213.86	4264.93	0.4089	2.7E-04
U6115_00760		phosphoribosylaminoimidazolesuccinocarboxamide synthase	8478.16	13621.93	0.6844	4.0E-08
U6115_00765		DNA alkylation repair protein	2163.64	2369.04	0.1312	4.1E-01

U6115_00770		SEL1-like repeat protein	4253.90	6012.06	0.4993	5.7E-07
U6115_00775		5-(carboxyamino)imidazole ribonucleotide synthase	10440.68	13581.94	0.3797	3.4E-03
U6115_00780	purE	5-(carboxyamino)imidazole ribonucleotide mutase	6860.89	8761.66	0.353	4.4E-03
U6115_00785		CPBP family intramembrane glutamic endopeptidase	1987.94	1829.36	-0.1194	4.9E-01
U6115_00790		hypothetical protein	323.88	555.80	0.7755	2.2E-05
U6115_00795		molybdopterin-dependent oxidoreductase	2188.63	3786.99	0.7906	5.7E-07
U6115_00800		hypothetical protein	2396.07	3347.79	0.4824	3.8E-03
U6115_00805		hypothetical protein	1093.81	813.90	-0.4278	2.3E-02
U6115_00810		hypothetical protein	4003.37	2309.33	-0.7938	2.6E-11
U6115_00815		histidinol-phosphate transaminase	9325.87	13579.37	0.5422	2.4E-08
U6115_00820		lipoprotein	1943.49	1294.01	-0.5858	1.9E-05
U6115_00825	cyaY	iron donor protein CyaY	1628.48	1025.87	-0.6662	3.0E-05
U6115_00830		ammonium transporter	3073.63	1858.72	-0.7259	1.5E-08
U6115_00835		sulfite exporter TauE/SafE family protein	1076.50	936.04	-0.2024	1.3E-01
U6115_00840		MBL fold metallo-hydrolase	3149.17	3122.23	-0.0125	9.3E-01
U6115_00845		tetratricopeptide repeat protein	795.57	841.97	0.0828	5.9E-01
U6115_00850	cysE	serine O-acetyltransferase	5912.50	4386.72	-0.4308	2.2E-05
U6115_00855		2OG-Fe(II) oxygenase family protein	4810.07	2711.14	-0.8272	4.1E-11
U6115_00860		MASE1 domain-containing protein	1899.78	2829.99	0.5748	4.2E-04
U6115_00865		MarR family transcriptional regulator	691.95	1436.35	1.0531	3.6E-14
U6115_00870		MDR family MFS transporter	6923.78	8894.93	0.3613	1.4E-02
U6115_00875	gcvA	transcriptional regulator GcvA	7476.80	7232.68	-0.0478	6.8E-01
U6115_00880		heme biosynthesis protein HemY	13873.71	14675.24	0.0812	5.2E-01
U6115_00885		uroporphyrinogen-III C-methyltransferase	12364.24	12075.51	-0.034	8.1E-01
U6115_00890		uroporphyrinogen-III synthase	761.31	1029.91	0.4369	5.0E-03
U6115_00895		DUF1697 domain-containing protein	1973.33	2351.48	0.2529	1.6E-01
U6115_00900	hemC	hydroxymethylbilane synthase	5728.02	5672.34	-0.0141	9.4E-01
U6115_00905	ppc	phosphoenolpyruvate carboxylase	8015.11	19459.36	1.2796	3.4E-39
U6115_00910		M28 family metallopeptidase	2301.69	5885.31	1.3543	3.3E-23
U6115_00915		M4 family metallopeptidase	1280.61	3374.37	1.3976	1.5E-09
U6115_00920	pdxH	pyridoxamine 5'-phosphate oxidase	4854.13	4034.49	-0.2664	1.5E-02
U6115_00925		S1/P1 nuclease	10578.97	11229.18	0.0861	4.1E-01
U6115_00930		LysR family transcriptional regulator	17385.88	20986.24	0.2715	3.0E-02

U6115_00935		DMT family transporter	667.11	939.38	0.4931	1.2E-04
U6115_00940		SNF2-related protein	2374.67	2061.14	-0.2042	3.2E-01
U6115_00945		hypothetical protein	4331.45	2381.33	-0.8637	1.7E-15
U6115_00950		hypothetical protein	6987.88	4042.31	-0.7898	3.4E-15
U6115_00955		hypothetical protein	2401.65	3289.94	0.4543	1.2E-02
U6115_00960	hemL	glutamate-1-semialdehyde 2,1-aminomutase	9617.81	8567.67	-0.1669	9.8E-02
U6115_00965		hypothetical protein	1464.33	823.40	-0.8304	4.0E-09
U6115_00970		CYTH domain-containing protein	2007.48	1231.05	-0.705	1.2E-10
U6115_00975		hypothetical protein	611.25	470.82	-0.3766	8.2E-02
U6115_00980		CopD family protein	826.84	653.15	-0.3409	9.1E-02
U6115_00985		MmcQ/YjbR family DNA-binding protein	932.49	712.25	-0.3899	3.2E-02
U6115_00990		M48 family metallopeptidase	3812.12	3397.71	-0.1662	3.4E-01
U6115_00995		rubredoxin	222.27	178.45	-0.3134	1.3E-01
U6115_01000		hydroxymethylpyrimidine/phosphomethylpyrimidine kinase	4023.23	4014.51	-0.0032	9.8E-01
U6115_01005	thiE	thiamine phosphate synthase	1327.93	1635.79	0.3012	3.1E-02
U6115_01010		hypothetical protein	4206.80	2257.96	-0.8976	6.8E-13
U6115_01015		hypothetical protein	1561.25	1361.40	-0.1969	1.3E-01
U6115_01020	pgi	glucose-6-phosphate isomerase	25488.16	22998.02	-0.1483	2.2E-01
U6115_01025	hexR	transcriptional regulator HexR	10116.26	6594.35	-0.6172	1.1E-07
U6115_01030		glucokinase	8844.81	9022.35	0.0288	8.4E-01
U6115_01035	pgl	6-phosphogluconolactonase	6625.78	8062.33	0.2829	7.9E-03
U6115_01040	zwf	glucose-6-phosphate dehydrogenase	30515.68	28309.78	-0.1082	3.3E-01
U6115_01045	edd	phosphogluconate dehydratase	34394.64	35733.89	0.0551	6.9E-01
U6115_01050		bifunctional 4-hydroxy-2-oxoglutarate aldolase/2-dehydro-3-deoxy-phosphogluconate aldolase	17216.03	19458.69	0.1768	2.0E-01
U6115_01055		DUF2867 domain-containing protein	860.17	1006.52	0.2259	1.5E-01
U6115_01060		helix-turn-helix domain-containing protein	1429.02	1651.41	0.2094	1.1E-01
U6115_01065		aminopeptidase P family protein	9988.62	10110.26	0.0174	9.1E-01
U6115_01070		benzoate/H(+) symporter BenE family transporter	391.71	424.28	0.1146	5.6E-01
U6115_01075		DHCW motif cupin fold protein	883.02	664.29	-0.4116	1.0E-02
U6115_01080		PLP-dependent aminotransferase family protein	1350.43	1356.28	0.0057	9.7E-01
U6115_01085		GNAT family N-acetyltransferase	258.71	293.18	0.1805	4.8E-01

U6115_01090		NAD(P)/FAD-dependent oxidoreductase	631.40	775.85	0.2964	3.5E-02
U6115_01095		DeoR family transcriptional regulator	3594.79	2827.29	-0.3462	6.2E-03
U6115_01100		EAL domain-containing protein	1996.88	2007.00	0.0081	9.5E-01
U6115_01105		LysR substrate-binding domain-containing protein	250.30	393.32	0.6508	7.5E-04
U6115_01110		O-methyltransferase	246.17	551.45	1.1627	1.1E-08
U6115_01115		glutathione S-transferase	879.83	2014.24	1.1951	9.2E-10
U6115_01120		hypothetical protein	2352.23	866.60	-1.4403	2.9E-34
U6115_01125		hypothetical protein	630.03	648.89	0.0445	8.1E-01
U6115_01130		hypothetical protein	1079.90	902.60	-0.2585	1.7E-01
U6115_01135		hypothetical protein	623.98	519.68	-0.2644	6.1E-02
U6115_01140		hypothetical protein	3716.44	1257.19	-1.5627	1.1E-32
U6115_01145		hypothetical protein	1525.28	713.12	-1.0966	2.9E-10
U6115_01150		hypothetical protein	749.45	440.22	-0.7668	2.9E-09
U6115_01155		hypothetical protein	1008.25	563.91	-0.8365	3.4E-11
U6115_01160	metH	methionine synthase	18056.41	10905.51	-0.7275	1.4E-11
U6115_01165		transporter substrate-binding domain-containing protein	944.03	727.59	-0.376	5.5E-03
U6115_01170		UvrD-helicase domain-containing protein	12942.82	8000.95	-0.6939	1.3E-13
U6115_01175		response regulator	4654.31	3177.81	-0.5509	2.0E-07
U6115_01180		LytTR family DNA-binding domain-containing protein	1925.90	1680.11	-0.1963	1.2E-01
U6115_01185		sensor domain-containing diguanylate cyclase	6201.81	5030.26	-0.3017	9.8E-03
U6115_01190		organic hydroperoxide resistance protein	2160.57	3337.96	0.6275	4.5E-10
U6115_01195		MarR family transcriptional regulator	3992.16	4025.75	0.0122	9.4E-01
U6115_01200		hypothetical protein	3320.32	4664.44	0.4913	1.5E-03
U6115_01205		YqiA/YcfP family alpha/beta fold hydrolase	3392.79	5624.52	0.7296	3.1E-08
U6115_01210		quinone oxidoreductase	4217.00	8784.03	1.0588	5.2E-28
U6115_01215		hypothetical protein	2650.86	3367.56	0.3454	3.1E-03
U6115_01220		hypothetical protein	2701.71	1711.17	-0.6581	8.7E-07
U6115_01225	ompR	two-component system response regulator OmpR	6597.25	6514.33	-0.0186	9.0E-01
U6115_01230		ATP-binding protein	4908.06	4623.12	-0.0863	4.6E-01
U6115_01235		DMT family transporter	336.60	318.60	-0.0807	7.4E-01
U6115_01240		methylated-DNA-[protein]-cysteine S-methyltransferase	322.31	357.17	0.1478	3.8E-01
U6115_01245		DUF1203 domain-containing protein	565.53	596.96	0.0755	6.5E-01

U6115_01250		CBS domain-containing protein	854.50	933.84	0.1265	3.3E-01
U6115_01255		hypothetical protein	5613.56	7772.54	0.4694	4.1E-04
U6115_01260	waaA	lipid IV(A) 3-deoxy-D-manno-octulosonic acid transferase	5171.98	4266.13	-0.2777	1.1E-01
U6115_01265	waaC	lipopolysaccharide heptosyltransferase I	8591.95	5053.68	-0.7655	2.3E-12
U6115_01270		GNAT family N-acetyltransferase	506.92	430.49	-0.236	1.7E-01
U6115_01275		S66 peptidase family protein	991.06	1092.27	0.1395	3.2E-01
U6115_01280		aminoglycoside 6'-N-acetyltransferase	2414.91	3671.86	0.6044	4.9E-07
U6115_01285		hemolysin family protein	3276.31	2176.77	-0.5899	9.2E-05
U6115_01290	ung	uracil-DNA glycosylase	3354.57	4648.94	0.4706	3.4E-03
U6115_01295		rhodanese-like domain-containing protein	5948.36	3239.04	-0.877	9.0E-14
U6115_01300		protein-L-isoaspartate O-methyltransferase	16357.48	9200.28	-0.8301	6.3E-14
U6115_01305	thiC	phosphomethylpyrimidine synthase ThiC	5606.39	2499.30	-1.1655	7.6E-24
U6115_01310		DUF2789 domain-containing protein	497.78	634.35	0.3481	1.1E-02
U6115_01315		GGDEF domain-containing protein	3730.99	4093.47	0.1336	2.7E-01
U6115_01320		GGDEF domain-containing protein	3561.18	3909.58	0.1347	3.7E-01
U6115_01325		PTS sugar transporter subunit IIA	8457.29	13409.08	0.6649	3.2E-08
U6115_01330		hypothetical protein	6887.89	10534.38	0.6127	5.2E-08
U6115_01335		EthD family reductase	543.29	1077.25	0.9889	8.7E-10
U6115_01340		winged helix-turn-helix domain-containing protein	1864.21	2275.79	0.288	3.5E-02
U6115_01345		VOC family protein	441.56	737.83	0.7401	1.4E-04
U6115_01350		transporter substrate-binding domain-containing protein	764.51	1001.88	0.3903	1.9E-03
U6115_01355	pyk	pyruvate kinase	31845.57	25070.61	-0.3451	6.7E-04
U6115_01360		hypothetical protein	117.23	105.29	-0.1588	5.7E-01
U6115_01365	glpK	glycerol kinase GlpK	6603.27	6085.56	-0.1177	4.9E-01
U6115_01370		MIP/aquaporin family protein	2745.63	2407.52	-0.1893	1.9E-01
U6115_01375	glpD	glycerol-3-phosphate dehydrogenase	8963.71	10495.91	0.2277	2.2E-01
U6115_01380		phenylalanine--tRNA ligase beta subunit-related protein	2796.55	3530.32	0.3363	5.0E-02
U6115_01385		methyl-accepting chemotaxis protein	2909.12	1777.54	-0.7104	1.3E-11
U6115_01390		Lrp/AsnC family transcriptional regulator	2499.11	1098.00	-1.186	8.7E-29
U6115_01395		DMT family transporter	797.64	810.35	0.0233	8.9E-01
U6115_01400	ugpC	sn-glycerol-3-phosphate ABC transporter ATP-binding protein UgpC	616.11	1052.94	0.7746	8.3E-09

U6115_01405	nagZ	beta-N-acetylhexosaminidase	389.52	815.02	1.0648	2.2E-08
U6115_01410		carbohydrate ABC transporter permease	219.82	294.97	0.4289	4.9E-02
U6115_01415		sugar ABC transporter permease	322.54	333.32	0.0492	8.0E-01
U6115_01420		extracellular solute-binding protein	497.71	1067.25	1.1032	4.9E-11
U6115_01425		OprD family outer membrane porin	537.67	457.77	-0.2345	1.1E-01
U6115_01430		methyl-accepting chemotaxis protein	734.40	580.43	-0.3378	1.0E-02
U6115_01435		U32 family peptidase	5740.73	3940.75	-0.5426	8.5E-05
U6115_01440		hypothetical protein	331.58	252.66	-0.3933	9.6E-02
U6115_01445		hypothetical protein	348.28	403.27	0.2126	1.8E-01
U6115_01450		MAPEG family protein	927.15	1402.92	0.5978	3.2E-05
U6115_01455		alkaline phosphatase family protein	1368.39	1421.07	0.0543	7.2E-01
U6115_01460		hypothetical protein	1669.88	1620.78	-0.0436	7.7E-01
U6115_01465		antibiotic biosynthesis monooxygenase family protein	1583.04	1807.02	0.1903	1.7E-01
U6115_01470		YkgJ family cysteine cluster protein	3663.38	4050.65	0.1448	2.5E-01
U6115_01475		hypothetical protein	3951.59	7572.41	0.9383	4.3E-12
U6115_01480		glycine zipper family protein	658.83	587.33	-0.1657	2.3E-01
U6115_01485		hypothetical protein	602.39	461.97	-0.3825	8.4E-03
U6115_01490		Spy/CpxP family protein refolding chaperone	623.02	472.73	-0.4016	1.6E-02
U6115_01495		TerB family tellurite resistance protein	2443.62	1781.94	-0.4558	4.4E-03
U6115_01500		VF530 family protein	697.72	415.88	-0.7478	6.5E-07
U6115_01505		DUF4337 domain-containing protein	844.45	725.98	-0.2175	1.4E-01
U6115_01510	gstA	glutathione transferase GstA	4392.60	12905.58	1.5551	1.5E-14
U6115_01515		nucleotide pyrophosphohydrolase	597.71	331.89	-0.8494	3.4E-08
U6115_01520		NAD-dependent protein deacylase	4720.25	2039.91	-1.21	5.0E-23
U6115_01525		mechanosensitive ion channel family protein	348.41	232.50	-0.5906	2.9E-03
U6115_01530		hypothetical protein	410.26	1257.65	1.6121	1.1E-25
U6115_01535		short chain dehydrogenase	390.34	637.05	0.7084	3.0E-07
U6115_01540		LysR family transcriptional regulator	1628.64	1475.86	-0.1422	2.9E-01
U6115_01545		hypothetical protein	840.69	1437.25	0.7716	1.1E-09
U6115_01550		indolepyruvate ferredoxin oxidoreductase family protein	8293.87	62484.50	2.9135	3.1E-70
U6115_01555	rarD	EamA family transporter RarD	2966.51	2683.68	-0.1444	3.3E-01
U6115_01560		hypothetical protein	245.53	281.59	0.2013	3.9E-01
U6115_01565		hypothetical protein	1296.22	1091.82	-0.247	3.1E-01

U6115_01570		GNAT family N-acetyltransferase	466.45	576.33	0.3046	7.2E-02
U6115_01575		M3 family metallopeptidase	5413.41	9150.00	0.7571	6.2E-08
U6115_01580		hypothetical protein	3052.12	4193.95	0.4577	1.8E-04
U6115_01585		hypothetical protein	1340.17	1204.02	-0.1557	2.5E-01
U6115_01590		glutathione binding-like protein	1434.17	1379.82	-0.0568	6.8E-01
U6115_01595		hypothetical protein	8540.88	7295.74	-0.2272	3.6E-02
U6115_01600		D-hexose-6-phosphate mutarotase	1481.71	1840.50	0.314	1.9E-02
U6115_01605		Mpo1-like protein	379.16	703.57	0.8927	7.5E-12
U6115_01610	hutC	histidine utilization repressor	8579.69	4049.54	-1.0831	3.9E-08
U6115_01615	hutI	imidazolonepropionase	1955.60	1305.97	-0.5827	6.4E-05
U6115_01620	hutG	formimidoylglutamase	1683.92	1630.69	-0.0465	8.4E-01
U6115_01625	hutU	urocanate hydratase	5641.08	11300.83	1.0024	2.0E-08
U6115_01630	hutH	histidine ammonia-lyase	3488.73	5212.66	0.5793	5.9E-04
U6115_01635		SUMF1/EgtB/PvdO family nonheme iron enzyme	1229.90	775.36	-0.6647	8.9E-08
U6115_01640	bla	class A beta-lactamase	3210.86	6320.57	0.9771	4.7E-07
U6115_01645		hypothetical protein	674.63	1373.92	1.0257	2.5E-07
U6115_01650		BPSS1780 family membrane protein	1778.72	2008.15	0.1744	2.3E-01
U6115_01655		amino acid aminotransferase	11177.67	15759.44	0.4956	2.7E-04
U6115_01660		superinfection immunity protein	1897.56	1605.71	-0.2406	3.6E-02
U6115_01665		GNAT family N-acetyltransferase	461.70	934.91	1.0185	1.1E-08
U6115_01670		alanyl-tRNA editing protein	400.56	953.84	1.252	4.8E-11
U6115_01675		DMT family transporter	428.83	731.88	0.7741	2.2E-05
U6115_01680		phage tail protein	3877.83	2225.44	-0.8012	1.2E-07
U6115_01685		hypothetical protein	2664.11	1556.03	-0.7762	1.0E-08
U6115_01690		tail assembly chaperone	2054.48	905.36	-1.182	1.0E-08
U6115_01695		tail fiber protein	3018.32	1296.19	-1.2195	1.7E-09
U6115_01700		putative phage tail protein	719.61	406.74	-0.8233	1.6E-05
U6115_01705		baseplate J/gp47 family protein	1886.75	1106.46	-0.7701	2.0E-06
U6115_01710		phage GP46 family protein	683.01	358.75	-0.9297	8.5E-07
U6115_01715		phage baseplate assembly protein V	789.70	415.30	-0.927	2.7E-10
U6115_01720		phage tail protein	652.02	323.08	-1.0121	2.0E-10
U6115_01725		DNA circularization N-terminal domain-containing protein	1766.07	778.42	-1.1818	4.7E-10

U6115_01730		hypothetical protein	3140.47	1198.87	-1.3897	1.7E-09
U6115_01735		phage tail protein	3026.05	1105.08	-1.4534	9.7E-20
U6115_01740		phage tail sheath C-terminal domain-containing protein	13522.16	5013.83	-1.4314	1.6E-13
U6115_01745		DUF1834 family protein	2582.07	1104.49	-1.2253	6.6E-13
U6115_01750		hypothetical protein	637.33	279.00	-1.1938	7.7E-11
U6115_01755		TraR/DksA family transcriptional regulator	271.06	116.81	-1.22	4.9E-10
U6115_01760		hypothetical protein	1829.43	955.26	-0.9382	2.3E-08
U6115_01765		hypothetical protein	395.49	221.95	-0.8354	1.6E-05
U6115_01770		M15 family metallopeptidase	2210.25	1145.84	-0.9481	2.5E-05
U6115_01775		Mor transcription activator family protein	4487.67	2170.19	-1.0485	3.3E-11
U6115_01780		helix-turn-helix transcriptional regulator	8592.11	4911.91	-0.807	1.0E-08
U6115_01785	gmhA	D-sedoheptulose 7-phosphate isomerase	5044.83	3696.84	-0.4488	4.7E-03
U6115_01790		alpha/beta hydrolase	3972.34	3567.54	-0.1553	3.2E-01
U6115_01795		HD domain-containing phosphohydrolase	1650.14	1300.59	-0.3444	4.5E-02
U6115_01800		SGNH/GDSL hydrolase family protein	2247.87	2988.53	0.4106	3.2E-02
U6115_01805		class I SAM-dependent methyltransferase	6585.16	3851.61	-0.774	2.2E-15
U6115_01810		SGNH/GDSL hydrolase family protein	3261.83	2421.74	-0.4296	3.2E-04
U6115_01815		anion transporter	1323.21	779.27	-0.7641	2.6E-08
U6115_01820		inositol monophosphatase family protein	3966.16	2556.32	-0.6329	2.2E-06
U6115_01825		16S rRNA (uracil(1498)-N(3))-methyltransferase	1631.68	1497.56	-0.1235	3.2E-01
U6115_01830		HD-GYP domain-containing protein	2577.59	2165.26	-0.251	2.4E-02
U6115_01835	pyrB	aspartate carbamoyltransferase	8142.54	6290.76	-0.3722	1.3E-02
U6115_01840	pyrI	aspartate carbamoyltransferase regulatory subunit	5033.95	3527.87	-0.5129	1.5E-02
U6115_01845		chloride channel protein	2808.39	2260.96	-0.3127	4.8E-02
U6115_01850		GNAT family N-acetyltransferase	3796.26	3317.40	-0.1949	1.9E-01
U6115_01855		NYN domain-containing protein	7367.40	4875.60	-0.5957	8.8E-07
U6115_01860		transporter substrate-binding domain-containing protein	535.00	576.07	0.1027	6.4E-01
U6115_01865		hypothetical protein	1125.53	747.01	-0.5903	1.6E-03
U6115_01870		DUF2059 domain-containing protein	26166.70	23192.71	-0.174	2.9E-01
U6115_01875		substrate-binding domain-containing protein	718.03	1461.78	1.0247	9.8E-11
U6115_01880		alpha/beta hydrolase	818.54	1173.23	0.5186	3.7E-05
U6115_01885		YgjP-like metallopeptidase domain-containing protein	2448.03	1045.17	-1.2275	6.1E-19

U6115_01890		YaeQ family protein	6203.59	3114.59	-0.9937	6.5E-16
U6115_01895		MliC family protein	7908.01	5423.18	-0.5443	1.1E-06
U6115_01900		DEAD/DEAH box helicase	32012.68	10804.75	-1.567	2.7E-30
U6115_01905		transporter substrate-binding domain-containing protein	6804.79	3301.62	-1.0434	7.0E-17
U6115_01910		TetR/AcrR family transcriptional regulator	2035.96	2029.53	-0.004	9.7E-01
U6115_01915		sensor domain-containing diguanylate cyclase	940.19	1016.60	0.1134	5.1E-01
U6115_01920		FUSC family protein	571.94	869.17	0.6039	4.5E-05
U6115_01925		pseudouridine synthase	8026.49	4178.72	-0.9416	2.3E-13
U6115_01930		NAD(P)H-dependent oxidoreductase	1245.75	883.23	-0.4966	2.2E-03
U6115_01935		LysR family transcriptional regulator	1104.18	1028.48	-0.1017	5.1E-01
U6115_01940		sigma-54-dependent Fis family transcriptional regulator	792.70	1169.14	0.5605	1.8E-05
U6115_01945	adh	aldehyde dehydrogenase	626.99	6733.58	3.4251	3.5E-35
U6115_01950		methyltransferase domain-containing protein	140.72	340.96	1.2754	1.3E-12
U6115_01955		methyl-accepting chemotaxis protein	7143.96	7692.76	0.1065	4.1E-01
U6115_01960		PAS domain-containing protein	3556.35	2824.54	-0.333	2.4E-03
U6115_01965		DUF3857 domain-containing protein	12371.07	20208.30	0.7079	1.9E-09
U6115_01970		biopolymer transporter ExbD	1455.85	4583.21	1.6553	1.0E-20
U6115_01975		MotA/TolQ/ExbB proton channel family protein	3074.39	9331.15	1.6025	4.9E-24
U6115_01980		energy transducer TonB	1113.60	2693.23	1.2759	1.2E-06
U6115_01985		TonB family protein	3139.52	9201.08	1.5519	6.2E-11
U6115_01990	hslV	ATP-dependent protease subunit HslV	4975.32	4756.25	-0.0648	7.0E-01
U6115_01995	hslU	ATP-dependent protease ATPase subunit HslU	11909.05	11356.33	-0.0686	6.8E-01
U6115_02000		MFS transporter	2128.42	2976.22	0.4838	1.9E-03
U6115_02005		hypothetical protein	246.29	127.59	-0.9511	2.2E-05
U6115_02010		16S ribosomal RNA	0.00	0.00		
U6115_02015		tRNA-Ile	0.00	0.00		
U6115_02020		tRNA-Ala	0.00	0.00		
U6115_02025		23S ribosomal RNA	0.00	0.00		
U6115_02030	rrf	5S ribosomal RNA	1.21	0.00	-2.868	3.6E-01
U6115_02035		branched-chain amino acid ABC transporter substrate-binding protein	11387.08	11170.54	-0.0278	8.6E-01
U6115_02040		efflux transporter outer membrane subunit	11220.00	23118.11	1.0428	2.6E-11

U6115_02045		efflux RND transporter permease subunit	26200.48	51028.50	0.9617	2.4E-11
U6115_02050		efflux RND transporter periplasmic adaptor subunit	12683.95	23735.48	0.9039	3.5E-10
U6115_02055		TetR family transcriptional regulator	6519.97	7026.95	0.1077	4.4E-01
U6115_02060		ABC transporter ATP-binding protein	11381.94	7198.64	-0.6609	1.1E-04
U6115_02065		ABC transporter permease	7316.73	3529.90	-1.0518	3.2E-16
U6115_02070		BolA/IbaG family iron-sulfur metabolism protein	3090.24	1355.04	-1.1905	3.1E-15
U6115_02075	murA	UDP-N-acetylglucosamine 1-carboxyvinyltransferase	18419.65	10960.83	-0.749	2.8E-06
U6115_02080		zinc ribbon domain-containing protein YjdM	3987.65	2077.43	-0.941	7.7E-09
U6115_02085		VacJ family lipoprotein	8566.33	8245.48	-0.0552	6.7E-01
U6115_02090		STAS domain-containing protein	2216.08	2337.75	0.0771	6.9E-01
U6115_02095		ABC transporter substrate-binding protein	17206.00	18390.45	0.096	4.8E-01
U6115_02100	mlaD	outer membrane lipid asymmetry maintenance protein MlaD	9105.51	7835.80	-0.2168	8.6E-02
U6115_02105	mlaE	lipid asymmetry maintenance ABC transporter permease subunit MlaE	14715.09	13582.32	-0.1155	5.1E-01
U6115_02110		ABC transporter ATP-binding protein	15076.42	12316.42	-0.2918	2.2E-02
U6115_02115	dapD	2,3,4,5-tetrahydropyridine-2,6-dicarboxylate N-succinyltransferase	14398.97	14373.56	-0.0026	9.9E-01
U6115_02120	dapC	succinyldiaminopimelate transaminase	4220.84	3478.24	-0.2789	8.6E-03
U6115_02125		DMT family transporter	1830.63	1444.04	-0.3415	6.7E-03
U6115_02130		GNAT family N-acetyltransferase	435.28	395.16	-0.137	4.1E-01
U6115_02135		hypothetical protein	1624.36	957.67	-0.7622	3.3E-08
U6115_02140		hypothetical protein	1586.89	3240.55	1.029	8.1E-12
U6115_02145		hypothetical protein	2102.65	1180.00	-0.8325	1.4E-11
U6115_02150		SlyX family protein	529.82	325.97	-0.6979	1.3E-06
U6115_02155		Na+/H+ antiporter NhaC family protein	2601.59	974.41	-1.4175	2.3E-22
U6115_02160		DUF2325 domain-containing protein	174.90	426.53	1.2824	1.9E-07
U6115_02165		hypothetical protein	338.29	679.21	1.0051	2.8E-12
U6115_02170		acyl-CoA thioesterase	723.79	536.88	-0.4311	1.8E-02
U6115_02175		helix-turn-helix transcriptional regulator	1230.46	983.99	-0.3202	2.1E-02
U6115_02180		DUF6058 family natural product biosynthesis protein	143.82	97.84	-0.5581	1.7E-02
U6115_02185		hypothetical protein	211.07	142.02	-0.568	3.9E-03
U6115_02190		antibiotic biosynthesis monooxygenase	247.95	139.55	-0.8202	8.4E-05
U6115_02195		class I SAM-dependent methyltransferase	939.57	678.31	-0.4691	6.7E-03

U6115_02200		Ig-like domain-containing protein	3459.74	4084.74	0.2393	3.8E-02
U6115_02205		TolC family protein	698.51	1067.16	0.6096	1.8E-06
U6115_02210		efflux RND transporter periplasmic adaptor subunit	253.74	398.04	0.654	3.3E-05
U6115_02215		HlyD family efflux transporter periplasmic adaptor subunit	244.81	391.38	0.6763	1.5E-03
U6115_02220		HlyD family efflux transporter periplasmic adaptor subunit	291.92	289.41	-0.0115	9.6E-01
U6115_02225		GNAT family N-acetyltransferase	69.13	123.29	0.8297	2.1E-03
U6115_02230		hypothetical protein	126.02	305.84	1.2894	5.1E-12
U6115_02235		tail fiber protein	793.94	2062.53	1.3772	4.4E-12
U6115_02240		DNA internalization-related competence protein ComEC/Rec2	831.87	1486.67	0.8373	1.1E-07
U6115_02245		response regulator	5015.68	6409.70	0.3536	7.2E-03
U6115_02250		ATP-binding protein	4338.27	4493.42	0.0508	6.8E-01
U6115_02255		ParA family protein	3293.05	3511.22	0.0927	4.2E-01
U6115_02260		surface-adhesin E family protein	3511.86	2630.33	-0.4173	6.2E-04
U6115_02265	recQ	DNA helicase RecQ	13337.97	6521.78	-1.0322	4.7E-20
U6115_02270	argJ	bifunctional glutamate N-acetyltransferase/amino-acid acetyltransferase ArgJ	8293.39	11656.30	0.4911	3.3E-04
U6115_02275		16S ribosomal RNA	0.00	0.00		
U6115_02280		tRNA-Ile	0.00	0.00		
U6115_02285		tRNA-Ala	0.00	0.00		
U6115_02290		23S ribosomal RNA	0.00	0.00		
U6115_02295	rrf	5S ribosomal RNA	0.00	0.00		
U6115_02300	pilV	type IV pilus modification protein PilV	1513.49	469.22	-1.6912	1.2E-30
U6115_02305		PilW family protein	3429.59	1447.47	-1.2452	1.1E-14
U6115_02310		PilX N-terminal domain-containing pilus assembly protein	1456.86	536.96	-1.4427	3.6E-18
U6115_02315		PilC/PilY family type IV pilus protein	8003.55	3526.88	-1.1826	6.1E-20
U6115_02320		type IV pilin protein	436.68	282.62	-0.6315	2.7E-05
U6115_02325		GspH/FimT family pseudopilin	548.81	292.54	-0.9088	1.6E-07
U6115_02330	rfaE2	D-glycero-beta-D-manno-heptose 1-phosphate adenylyltransferase	4722.23	3009.45	-0.6503	5.4E-06
U6115_02335		biotin-[acetyl-CoA-carboxylase] ligase	3471.79	2226.59	-0.6408	7.2E-05

U6115_02340		type III pantothenate kinase	1322.99	895.65	-0.5617	2.5E-03
U6115_02345		SPOR domain-containing protein	6983.62	4676.02	-0.5785	2.3E-06
U6115_02350		YoaK family protein	5820.03	4875.35	-0.2558	5.6E-02
U6115_02355		hypothetical protein	2951.48	3619.27	0.2942	1.0E-01
U6115_02360	cobS	adenosylcobinamide-GDP ribazoletransferase	1803.91	2001.75	0.1495	2.3E-01
U6115_02365		histidine phosphatase family protein	1051.87	1208.42	0.2006	2.5E-01
U6115_02370	cobT	nicotinate-nucleotide-dimethylbenzimidazole phosphoribosyltransferase	3367.64	3579.39	0.0878	4.6E-01
U6115_02375		TonB-dependent receptor	12657.23	6332.20	-0.9993	4.2E-15
U6115_02380	cobU	bifunctional adenosylcobinamide kinase/adenosylcobinamide-phosphate guanylyltransferase	1129.52	644.00	-0.8102	1.2E-04
U6115_02385		cobalamin-binding protein	1772.62	994.36	-0.834	8.2E-10
U6115_02390		hypothetical protein	2020.57	1356.75	-0.574	4.3E-06
U6115_02395		cell division protein ZapA	3375.73	2060.47	-0.712	2.6E-03
U6115_02400	ssrS	6S RNA	18976.54	26156.96	0.463	6.8E-01
U6115_02405		5-formyltetrahydrofolate cyclo-ligase	2790.98	1923.25	-0.5374	5.2E-04
U6115_02410		DUF1294 domain-containing protein	750.70	610.71	-0.2979	1.2E-01
U6115_02415		vir-repressed protein	245.52	177.89	-0.4686	1.1E-02
U6115_02420		EVE domain-containing protein	3638.39	3092.65	-0.2344	2.4E-02
U6115_02425		sulfite exporter TauE/SafE family protein	1375.44	1528.95	0.1527	5.0E-01
U6115_02430	leuS	leucine--tRNA ligase	30346.73	27930.37	-0.1197	3.0E-01
U6115_02435	lptE	LPS assembly lipoprotein LptE	2873.10	3623.21	0.334	2.3E-03
U6115_02440	holA	DNA polymerase III subunit delta	5187.76	6346.05	0.2908	1.0E-01
U6115_02445		MFS transporter	560.69	529.85	-0.083	5.9E-01
U6115_02450		LysR family transcriptional regulator	567.58	877.69	0.6267	2.0E-03
U6115_02455		GNAT family N-acetyltransferase	250.05	331.74	0.4069	5.5E-02
U6115_02460		MFS transporter	1176.34	1629.62	0.4711	6.9E-03
U6115_02465		hypothetical protein	1561.12	1281.32	-0.2855	8.3E-02
U6115_02470	rlmH	23S rRNA (pseudouridine(1915)-N(3))-methyltransferase RlmH	1915.54	1142.96	-0.7454	5.4E-06
U6115_02475	rsfS	ribosome silencing factor	8088.20	3449.18	-1.2297	5.7E-16
U6115_02480	nadD	nicotinate-nucleotide adenylyltransferase	8756.08	7035.89	-0.3154	3.4E-02
U6115_02485		carbonic anhydrase	12946.13	14918.51	0.2046	1.7E-01

U6115_02490		acetate uptake transporter	3175.97	1188.49	-1.418	2.1E-29
U6115_02495		hypothetical protein	838.39	762.77	-0.1356	4.3E-01
U6115_02500		DUF2804 domain-containing protein	1264.41	1615.63	0.3544	8.9E-03
U6115_02505		M48 family metalloprotease	6360.06	8850.68	0.4769	1.4E-06
U6115_02510	aceE	pyruvate dehydrogenase (acetyl-transferring). homodimeric type	120343.70	144574.38	0.2646	3.8E-02
U6115_02515	aceF	dihydrolipoyllysine-residue acetyltransferase	36241.85	48916.46	0.4326	4.2E-04
U6115_02520	lpdA	dihydrolipoyl dehydrogenase	33033.99	41287.51	0.3217	7.1E-03
U6115_02525		hypothetical protein	181.07	195.88	0.1164	6.8E-01
U6115_02530		transposase	437.57	494.32	0.1772	2.7E-01
U6115_02535		hypothetical protein	32.76	27.50	-0.2786	5.3E-01
U6115_02540		hypothetical protein	1554.99	948.82	-0.7127	5.0E-03
U6115_02545		hypothetical protein	1023.22	4089.50	2	6.8E-31
U6115_02550		hypothetical protein	38.10	175.57	2.2255	4.1E-18
U6115_02555		HAD family hydrolase	305.16	1030.81	1.7604	3.7E-28
U6115_02560		GGDEF domain-containing protein	825.33	1540.51	0.8997	2.5E-14
U6115_02565		DinB family protein	1717.50	2433.35	0.5032	7.3E-06
U6115_02570		TetR/AcrR family transcriptional regulator	1066.42	1207.84	0.1803	1.8E-01
U6115_02575		hypothetical protein	641.49	692.38	0.112	5.3E-01
U6115_02580		hypothetical protein	531.36	541.35	0.0308	8.8E-01
U6115_02585		autotransporter assembly complex family protein	10819.10	9068.37	-0.2547	6.8E-02
U6115_02590		translocation/assembly module TamB domain-containing protein	8435.68	7026.67	-0.2636	1.8E-01
U6115_02595		efflux transporter outer membrane subunit	2385.36	2320.26	-0.0397	7.8E-01
U6115_02600		FUSC family protein	1687.92	1508.56	-0.1624	2.9E-01
U6115_02605		DUF1656 domain-containing protein	88.13	66.72	-0.3901	1.4E-01
U6115_02610		efflux RND transporter periplasmic adaptor subunit	1568.86	1474.63	-0.0885	4.8E-01
U6115_02615		hypothetical protein	2905.05	2386.25	-0.2834	7.0E-02
U6115_02620		HAMP domain-containing sensor histidine kinase	3895.24	3350.66	-0.2167	9.9E-02
U6115_02625		helix-turn-helix domain-containing protein	5304.98	3456.18	-0.6175	2.4E-06
U6115_02630		hypothetical protein	1509.02	1784.08	0.2427	1.2E-01
U6115_02635		MarR family transcriptional regulator	964.71	848.57	-0.1847	2.3E-01
U6115_02640		isochorismatase family cysteine hydrolase	1059.82	1206.88	0.189	1.6E-01

U6115_02645		EAL domain-containing protein	6883.65	7069.70	0.0387	7.9E-01
U6115_02650	rnpB	RNase P RNA component class A	139853.70	185784.70	0.4097	9.9E-02
U6115_02655	dusB	tRNA dihydrouridine synthase DusB	11215.92	9375.25	-0.2585	2.1E-02
U6115_02660		helix-turn-helix domain-containing protein	1250.96	896.67	-0.4808	4.7E-03
U6115_02665	purH	bifunctional phosphoribosylaminoimidazolecarboxamide formyltransferase/IMP cyclohydrolase	11303.97	13823.94	0.2903	7.9E-03
U6115_02670	purD	phosphoribosylamine-glycine ligase	6682.25	6950.41	0.0567	6.4E-01
U6115_02675		hypothetical protein	338.35	455.21	0.4261	2.1E-02
U6115_02680		L-threonylcarbamoyladenylate synthase	4965.94	2551.62	-0.9603	5.5E-17
U6115_02685	tolQ	protein TolQ	9844.92	10083.57	0.0344	8.2E-01
U6115_02690	yedA	drug/metabolite exporter YedA	1259.32	1227.38	-0.0372	8.6E-01
U6115_02695		lytic polysaccharide monooxygenase	12993.46	11756.67	-0.1445	3.1E-01
U6115_02700		GntR family transcriptional regulator	2077.12	2061.83	-0.0113	9.5E-01
U6115_02705	nagA	N-acetylglucosamine-6-phosphate deacetylase	1379.64	1544.46	0.1612	2.1E-01
U6115_02710		SIS domain-containing protein	1035.90	1676.17	0.6939	5.7E-07
U6115_02715	ptsP	phosphoenolpyruvate--protein phosphotransferase	1213.26	1632.66	0.4287	5.5E-03
U6115_02720	nagE	N-acetylglucosamine-specific PTS transporter subunit IIBC	2361.81	4273.97	0.8553	4.8E-14
U6115_02725	gap	type I glyceraldehyde-3-phosphate dehydrogenase	255843.59	242156.55	-0.0793	6.5E-01
U6115_02730		Lrp/AsnC family transcriptional regulator	3548.25	2643.85	-0.4245	8.0E-04
U6115_02735	phoB	phosphate regulon transcriptional regulator PhoB	12369.38	7531.67	-0.7156	1.1E-06
U6115_02740	phoR	phosphate regulon sensor histidine kinase PhoR	12865.61	7759.38	-0.7294	4.2E-05
U6115_02745	tal	transaldolase	27204.73	17049.80	-0.6741	1.5E-09
U6115_02750		hemerythrin domain-containing protein	3136.04	4610.55	0.5562	1.2E-07
U6115_02755	ampD	1,6-anhydro-N-acetylmuramyl-L-alanine amidase AmpD	2683.20	3140.83	0.2267	9.4E-02
U6115_02760		hypothetical protein	523.03	531.87	0.023	8.9E-01
U6115_02765		chorismate-binding protein	1024.94	1023.95	-0.0017	9.9E-01
U6115_02770		queuosine precursor transporter	713.87	695.69	-0.0391	8.1E-01
U6115_02775		helix-turn-helix transcriptional regulator	747.44	632.81	-0.2409	1.0E-01
U6115_02780		phosphohydrolase	1419.05	1052.66	-0.4313	7.1E-04
U6115_02785	kup	low affinity potassium transporter Kup	16162.46	13464.10	-0.2635	9.2E-03
U6115_02790		proline-tRNA ligase	18661.77	16806.90	-0.151	1.7E-01
U6115_02795		transglycosylase SLT domain-containing protein	4049.77	3307.63	-0.2915	8.5E-03

U6115_02800		Nramp family divalent metal transporter	4421.78	4549.94	0.0415	7.3E-01
U6115_02805		MarR family transcriptional regulator	8123.00	7205.35	-0.1728	2.0E-01
U6115_02810	guaD	guanine deaminase	13551.18	7535.73	-0.8468	2.4E-11
U6115_02815		hypothetical protein	1130.90	909.31	-0.3154	1.8E-02
U6115_02820		carboxymuconolactone decarboxylase family protein	1113.73	904.62	-0.3006	1.2E-01
U6115_02825		LysR family transcriptional regulator	1371.49	835.28	-0.7155	5.3E-06
U6115_02830		RDD family protein	1923.15	1249.96	-0.6212	6.8E-05
U6115_02835		DUF3106 domain-containing protein	1737.71	985.14	-0.8192	4.8E-08
U6115_02840		DUF3619 family protein	1470.97	1086.78	-0.4358	8.3E-04
U6115_02845		RNA polymerase sigma factor	7820.85	4166.24	-0.9083	2.4E-16
U6115_02850	ilvB	biosynthetic-type acetolactate synthase large subunit	38577.67	39227.27	0.0241	9.0E-01
U6115_02855	ilvN	acetolactate synthase small subunit	12202.93	10627.58	-0.1994	4.1E-01
U6115_02860	ilvC	ketol-acid reductoisomerase	43550.12	36120.63	-0.2698	3.6E-02
U6115_02865		ferritin-like domain-containing protein	704.14	623.93	-0.1752	2.3E-01
U6115_02870	asd	archaetidylserine decarboxylase	6096.50	3655.65	-0.7375	9.2E-12
U6115_02875		DUF2860 family protein	9239.24	16313.33	0.8203	1.3E-05
U6115_02880		2-isopropylmalate synthase	4650.45	5390.10	0.2129	7.9E-02
U6115_02885		hypothetical protein	351.76	411.33	0.2272	1.4E-01
U6115_02890		S66 peptidase family protein	968.08	1006.81	0.0581	6.6E-01
U6115_02895	bioD	dethiobiotin synthase	1274.09	903.63	-0.4941	1.7E-04
U6115_02900	blaOXA	class D beta-lactamase	4507.86	6947.40	0.6239	1.1E-03
U6115_02905	coxB	cytochrome c oxidase subunit II	1374.90	1622.05	0.2375	7.2E-02
U6115_02910	ctaD	cytochrome c oxidase subunit I	1151.75	1612.54	0.4845	5.7E-05
U6115_02915		cytochrome c oxidase assembly protein	216.87	367.01	0.7569	2.2E-05
U6115_02920		DUF2970 domain-containing protein	43.91	74.25	0.7563	1.4E-02
U6115_02925		cytochrome c oxidase subunit 3	3472.45	3137.78	-0.1464	4.1E-01
U6115_02930		DUF2909 family protein	1152.95	1114.10	-0.0494	7.1E-01
U6115_02935		SURF1 family protein	207.27	467.43	1.1697	3.5E-11
U6115_02940		hypothetical protein	224.60	309.82	0.4581	1.1E-02
U6115_02945		COX15/CtaA family protein	222.01	496.53	1.1597	2.0E-09
U6115_02950		heme o synthase	384.08	606.34	0.6593	2.2E-05
U6115_02955		SCO family protein	6191.21	2548.53	-1.2806	1.8E-28
U6115_02960		flagellar brake protein	13706.76	7110.39	-0.9469	5.9E-15

U6115_02965	hisG	ATP phosphoribosyltransferase	6032.69	2912.54	-1.0506	4.3E-19
U6115_02970	hisD	histidinol dehydrogenase	6528.80	4600.52	-0.5051	2.6E-06
U6115_02975	hisC	histidinol-phosphate transaminase	7077.67	6594.34	-0.1019	3.7E-01
U6115_02980	hisB	imidazoleglycerol-phosphate dehydratase HisB	5391.21	4412.90	-0.2888	2.1E-02
U6115_02985	hisH	imidazole glycerol phosphate synthase subunit HisH	3444.58	2376.46	-0.5354	1.3E-06
U6115_02990	hisA	1-(5-phosphoribosyl)-5-[(5-phosphoribosylamino)methylideneamino]imidazole-4-carboxamide isomerase	10766.64	7127.84	-0.595	1.8E-05
U6115_02995	hisF	imidazole glycerol phosphate synthase subunit HisF	7050.84	3954.84	-0.8344	2.7E-15
U6115_03000	hisI	phosphoribosyl-AMP cyclohydrolase	2130.03	1329.30	-0.6811	9.0E-09
U6115_03005		phosphoribosyl-ATP diphosphatase	9656.00	8400.78	-0.201	1.2E-01
U6115_03010		histidine triad nucleotide-binding protein	7413.95	7404.90	-0.002	9.9E-01
U6115_03015	tatA	Sec-independent protein translocase subunit TatA	5541.01	6962.47	0.3292	2.4E-02
U6115_03020	tatB	Sec-independent protein translocase protein TatB	4837.65	5070.68	0.068	7.2E-01
U6115_03025	tatC	twin-arginine translocase subunit TatC	6635.95	4706.74	-0.4956	4.7E-05
U6115_03030		DUF2069 domain-containing protein	958.34	608.47	-0.6545	3.4E-04
U6115_03035		methyl-accepting chemotaxis protein	2402.35	1873.43	-0.3594	1.7E-03
U6115_03040		TatD family hydrolase	758.05	800.22	0.0794	6.7E-01
U6115_03045		hypothetical protein	1175.33	1022.05	-0.2009	1.5E-01
U6115_03050		NUDIX hydrolase	438.78	521.56	0.2514	1.1E-01
U6115_03055		ABC transporter transmembrane domain-containing protein	1061.00	1529.33	0.5279	1.9E-04
U6115_03060		ATP-binding cassette domain-containing protein	1110.74	1272.93	0.1971	2.1E-01
U6115_03065		HPP family protein	1234.60	1041.34	-0.2467	1.1E-01
U6115_03070		MarR family transcriptional regulator	1212.56	1035.60	-0.2284	1.9E-01
U6115_03075		chloride channel protein	2319.84	2379.76	0.0369	8.6E-01
U6115_03080		bestrophin family ion channel	3784.42	2193.19	-0.7866	4.1E-08
U6115_03085		hypothetical protein	2000.22	1847.63	-0.1141	4.0E-01
U6115_03090		hypothetical protein	14325.44	43817.26	1.613	9.4E-04
U6115_03095	mgtE	magnesium transporter	14921.08	36957.92	1.3086	2.4E-06
U6115_03100	ndhC	NADH-quinone oxidoreductase subunit A	673.57	1307.49	0.9591	1.3E-08
U6115_03105		hypothetical protein	1643.19	3683.72	1.1651	9.6E-12
U6115_03110		hypothetical protein	504.44	473.00	-0.0919	5.4E-01

U6115_03115		DUF1484 family protein	2175.56	1774.72	-0.2932	4.3E-02
U6115_03120		TIGR04141 family sporadically distributed protein	961.28	1235.79	0.3622	1.7E-01
U6115_03125		IS110 family transposase	386.74	424.44	0.1341	5.0E-01
U6115_03130		hypothetical protein	3055.48	1368.64	-1.1588	2.5E-11
U6115_03135		hypothetical protein	1207.27	568.61	-1.0864	1.8E-12
U6115_03140		hypothetical protein	1457.05	848.88	-0.7799	3.9E-03
U6115_03145		IS5 family transposase	949.72	1198.69	0.3346	3.8E-02
U6115_03150		tRNA-Leu	244.20	148.15	-0.716	2.1E-02
U6115_03155	rsmI	16S rRNA (cytidine(1402)-2'-O)-methyltransferase	4740.82	2317.75	-1.0324	1.4E-12
U6115_03160		penicillin-binding protein activator	2305.60	2433.81	0.078	6.4E-01
U6115_03165		YraN family protein	1178.68	1209.84	0.0391	8.2E-01
U6115_03170		phosphoheptose isomerase	5911.88	7082.28	0.2604	6.4E-02
U6115_03175		BON domain-containing protein	8271.43	11739.73	0.5052	4.7E-06
U6115_03180		hypothetical protein	686.56	1080.49	0.6546	2.0E-06
U6115_03185		EAL domain-containing protein	4361.12	6121.42	0.4888	1.0E-05
U6115_03190		Tim44-like domain-containing protein	59991.10	152205.89	1.3432	3.5E-18
U6115_03195	tsaA	tRNA (N6-threonylcarbamoyladenosine(37)-N6)-methyltransferase TrmO	2928.20	2096.45	-0.4816	1.5E-05
U6115_03200		GNAT family N-acyltransferase	397.94	1589.24	2.0014	5.2E-27
U6115_03205	mnmG	tRNA uridine-5-carboxymethylaminomethyl(34) synthesis enzyme MnmG	17252.70	10649.67	-0.696	2.0E-09
U6115_03210	rsmG	16S rRNA (guanine(527)-N(7))-methyltransferase RsmG	6735.57	4327.94	-0.6381	5.8E-05
U6115_03215		ParA family protein	6849.36	4945.71	-0.4697	3.3E-03
U6115_03220		ParB/RepB/Spo0J family partition protein	5164.29	3733.03	-0.4685	2.2E-05
U6115_03225		ATP synthase subunit I	6875.27	5142.12	-0.4186	1.1E-03
U6115_03230	atpB	F0F1 ATP synthase subunit A	39476.58	26516.82	-0.5741	2.2E-05
U6115_03235	atpE	F0F1 ATP synthase subunit C	51761.37	28630.68	-0.8543	5.4E-07
U6115_03240		F0F1 ATP synthase subunit B	43838.89	25108.26	-0.804	1.4E-08
U6115_03245		F0F1 ATP synthase subunit delta	60652.37	30510.62	-0.9912	3.3E-14
U6115_03250	atpA	F0F1 ATP synthase subunit alpha	193268.17	102124.99	-0.9203	1.9E-14
U6115_03255	atpG	F0F1 ATP synthase subunit gamma	91723.20	47588.32	-0.9467	1.5E-11
U6115_03260	atpD	F0F1 ATP synthase subunit beta	201815.22	107017.81	-0.9152	2.9E-14
U6115_03265		F0F1 ATP synthase subunit epsilon	46275.76	25390.83	-0.866	3.5E-12

U6115_03270	glmU	bifunctional UDP-N-acetylglucosamine diphosphorylase/glucosamine-1-phosphate N-acetyltransferase GlmU	8604.09	4681.94	-0.8781	4.4E-16
U6115_03275		DUF6404 family protein	193.75	139.88	-0.4798	2.1E-02
U6115_03280		DeoR family transcriptional regulator	4901.85	1721.73	-1.5097	4.7E-22
U6115_03285	glmS	glutamine-fructose-6-phosphate transaminase (isomerizing)	21070.93	9943.36	-1.0836	1.7E-16
U6115_03290		hypothetical protein	1227.32	1733.53	0.4986	2.9E-03
U6115_03295		transporter substrate-binding domain-containing protein	2906.98	7198.66	1.3084	3.1E-31
U6115_03300		patatin-like phospholipase family protein	13280.53	53531.01	2.011	1.2E-59
U6115_03305		3-hydroxybutyrate dehydrogenase	19885.91	73360.82	1.8832	9.3E-35
U6115_03310		hypothetical protein	5590.77	2857.82	-0.9677	3.5E-10
U6115_03315		transporter substrate-binding domain-containing protein	3424.09	2237.73	-0.6138	1.6E-05
U6115_03320		LysR family transcriptional regulator	3383.46	2973.36	-0.1867	7.8E-02
U6115_03325		MFS transporter	461.52	342.46	-0.4338	3.3E-02
U6115_03330	dkgB	2,5-didehydrogluconate reductase DkgB	1986.87	3379.62	0.7665	4.3E-10
U6115_03335		FKBP-type peptidyl-prolyl cis-trans isomerase	1885.21	1126.63	-0.7419	2.0E-09
U6115_03340		hypothetical protein	3220.52	2017.08	-0.6748	8.6E-05
U6115_03345		hypothetical protein	662.90	901.25	0.4415	7.4E-03
U6115_03350		DHA2 family efflux MFS transporter permease subunit	2589.40	8552.51	1.7236	3.7E-34
U6115_03355		HlyD family secretion protein	1680.59	7163.88	2.0911	3.4E-35
U6115_03360		efflux transporter outer membrane subunit	3912.95	8525.22	1.1228	2.7E-09
U6115_03365		TetR/AcrR family transcriptional regulator	2599.04	2462.81	-0.0778	5.5E-01
U6115_03370		DinB family protein	3246.11	3277.23	0.0136	9.5E-01
U6115_03375		methyl-accepting chemotaxis protein	3590.16	3479.75	-0.0452	7.6E-01
U6115_03380		LysR family transcriptional regulator	1068.88	750.97	-0.5105	4.2E-05
U6115_03385		MFS transporter	802.34	393.30	-1.0285	1.2E-14
U6115_03390		hypothetical protein	308.34	397.18	0.3615	4.6E-02
U6115_03395		NAD(P)-dependent oxidoreductase	1648.10	5085.13	1.6253	9.5E-21
U6115_03400		LysR family transcriptional regulator	794.57	676.28	-0.2327	1.6E-01
U6115_03405		LysR family transcriptional regulator	1361.25	920.51	-0.5642	1.1E-06
U6115_03410		MFS transporter	488.34	551.68	0.1759	2.9E-01

U6115_03415		LysR family transcriptional regulator	486.67	350.97	-0.4709	1.6E-02
U6115_03420		hypothetical protein	429.80	347.83	-0.3007	2.7E-01
U6115_03425		lysozyme	1424.53	1542.46	0.1156	6.0E-01
U6115_03430		hypothetical protein	646.83	585.74	-0.1406	4.5E-01
U6115_03435		PHB depolymerase family esterase	727.73	955.42	0.394	7.5E-02
U6115_03440		hypothetical protein	360.09	283.11	-0.3426	4.5E-02
U6115_03445		transposase	16.20	13.99	-0.2342	6.8E-01
U6115_03450		IS3 family transposase	0.00	0.00		
U6115_03455		IS5 family transposase	1983.42	1076.64	-0.8814	3.3E-09
U6115_03460		DUF4239 domain-containing protein	2861.00	1995.96	-0.5191	8.5E-03
U6115_03465		DUF2589 domain-containing protein	10195.69	7506.23	-0.4417	1.0E-01
U6115_03470		DUF2589 domain-containing protein	1752.59	1207.32	-0.5365	6.1E-03
U6115_03475		hypothetical protein	1552.20	1065.03	-0.542	1.9E-02
U6115_03480		hypothetical protein	4745.02	4932.17	0.0561	8.3E-01
U6115_03485		hypothetical protein	2795.02	3078.83	0.1398	6.6E-01
U6115_03490		hypothetical protein	1003.46	927.20	-0.1136	4.5E-01
U6115_03495		sigma-70 family RNA polymerase sigma factor	950.41	872.97	-0.1212	3.9E-01
U6115_03500		Crp/Fnr family transcriptional regulator	794.76	763.31	-0.0555	7.2E-01
U6115_03505		tRNA-Tyr	48.23	34.55	-0.47	3.5E-01
U6115_03510	fghA	S-formylglutathione hydrolase	1782.56	1783.17	-0.0004	1.0E+00
U6115_03515		S-(hydroxymethyl)glutathione dehydrogenase/class III alcohol dehydrogenase	4137.71	4776.20	0.2068	1.1E-01
U6115_03520		LysR family transcriptional regulator	640.04	575.07	-0.1562	3.1E-01
U6115_03525		nucleoid-associated protein	4282.02	2588.41	-0.7262	1.6E-09
U6115_03530		hypothetical protein	934.42	615.45	-0.6031	9.3E-05
U6115_03535		DUF805 domain-containing protein	8079.69	1415.19	-2.5128	1.8E-57
U6115_03540		4-deoxy-4-formamido-L-arabinose-phosphoundecaprenol deformylase	3636.41	1566.10	-1.215	1.1E-31
U6115_03545		GrpB family protein	1345.64	487.03	-1.4669	3.9E-24
U6115_03550		hypothetical protein	4586.33	2492.38	-0.8802	1.2E-13
U6115_03555		bifunctional UDP-4-keto-pentose/UDP-xylose synthase	21413.13	13259.38	-0.6916	1.5E-06
U6115_03560		formyltransferase	7041.13	5958.52	-0.2408	3.8E-02
U6115_03565		glycosyltransferase	4583.34	3115.45	-0.5574	5.1E-07

U6115_03570		DegT/DnrJ/EryC1/StrS aminotransferase family protein	3702.46	2651.22	-0.4824	1.2E-05
U6115_03575		SMR family transporter	1750.86	951.94	-0.8783	2.7E-11
U6115_03580		glycosyltransferase family 39 protein	6695.97	4273.23	-0.6478	1.9E-09
U6115_03585	hemF	oxygen-dependent coproporphyrinogen oxidase	4449.06	3177.59	-0.485	3.4E-05
U6115_03590		porin	2688.59	1328.14	-1.0185	2.2E-08
U6115_03595		alpha/beta fold hydrolase	6505.37	4372.20	-0.5734	2.1E-04
U6115_03600		ATP-binding protein	7055.00	4091.27	-0.7856	3.6E-10
U6115_03605		winged helix-turn-helix domain-containing protein	6450.97	4957.00	-0.3798	1.1E-03
U6115_03610		carbon starvation CstA family protein	5473.99	10398.20	0.9258	1.3E-16
U6115_03615		YbdD/YjiX family protein	197.48	341.20	0.7937	3.7E-06
U6115_03620		DUF2339 domain-containing protein	7671.75	6957.86	-0.1407	3.3E-01
U6115_03625		YeiH family putative sulfate export transporter	1623.77	1929.98	0.249	1.0E-01
U6115_03630		LysR family transcriptional regulator	476.38	518.30	0.1212	4.6E-01
U6115_03635		DHA2 family efflux MFS transporter permease subunit	8690.74	15278.59	0.8139	8.4E-10
U6115_03640		EmrA/EmrK family multidrug efflux transporter periplasmic adaptor subunit	4403.29	9181.98	1.0599	6.9E-08
U6115_03645		efflux transporter outer membrane subunit	12564.49	18242.06	0.5378	6.4E-03
U6115_03650		MarR family transcriptional regulator	10765.04	7964.75	-0.4346	1.2E-04
U6115_03655	arsC	arsenate reductase (glutaredoxin)	8507.45	10255.11	0.2696	1.8E-02
U6115_03660		multidrug effflux MFS transporter	3717.38	4245.05	0.1917	1.7E-01
U6115_03665		Wzz/FepE/Etk N-terminal domain-containing protein	1352.74	7838.00	2.5339	3.4E-85
U6115_03670		SLBB domain-containing protein	1087.09	10885.03	3.3238	6.0E-71
U6115_03675		YjbH domain-containing protein	1684.88	13852.53	3.0396	9.4E-60
U6115_03680		hypothetical protein	607.55	5618.13	3.2089	6.0E-49
U6115_03685		hypothetical protein	5171.08	50504.71	3.288	3.3E-58
U6115_03690	thrB	homoserine kinase	11260.84	10635.17	-0.0824	5.0E-01
U6115_03695		DUF2782 domain-containing protein	6132.26	5809.92	-0.0779	4.9E-01
U6115_03700		TIGR00730 family Rossman fold protein	14436.33	10112.67	-0.5136	1.2E-03
U6115_03705	polA	DNA polymerase I	15616.98	12061.73	-0.3727	5.9E-04
U6115_03710		GspH/FimT family pseudopilin	113.68	279.11	1.2929	1.5E-09
U6115_03715		hypothetical protein	44.01	112.28	1.3358	1.4E-05
U6115_03720		hypothetical protein	123.41	275.54	1.1552	1.6E-05
U6115_03725		pilus assembly protein	223.98	284.85	0.3472	4.8E-02

U6115_03730		type IV pilin protein	517.89	535.61	0.0511	7.7E-01
U6115_03735		AmpG family muropeptide MFS transporter	3303.34	3858.39	0.2237	4.4E-02
U6115_03740		YqaE/Pmp3 family membrane protein	1874.90	1735.67	-0.111	5.0E-01
U6115_03745	metW	methionine biosynthesis protein MetW	2122.20	2288.28	0.1091	4.4E-01
U6115_03750		homoserine O-acetyltransferase	9039.24	8280.50	-0.1265	3.6E-01
U6115_03755		hypothetical protein	630.69	588.64	-0.0975	5.7E-01
U6115_03760		hypothetical protein	1187.82	1020.41	-0.2185	7.0E-02
U6115_03765		hypothetical protein	1904.27	1112.56	-0.7741	1.1E-08
U6115_03770	msrP	protein-methionine-sulfoxide reductase catalytic subunit MsrP	2959.51	7636.79	1.3674	8.9E-21
U6115_03775		protein-methionine-sulfoxide reductase heme-binding subunit MsrQ	842.17	2413.77	1.5197	1.4E-30
U6115_03780	ybaK	Cys-tRNA(Pro) deacylase	1218.92	1795.74	0.5585	6.5E-04
U6115_03785		transporter substrate-binding domain-containing protein	1592.94	1886.09	0.2441	4.7E-02
U6115_03790		4Fe-4S binding protein	8820.78	9584.73	0.12	3.8E-01
U6115_03795		FTR1 family protein	5667.29	6860.27	0.2758	1.0E-01
U6115_03800		cupredoxin domain-containing protein	5214.83	5265.34	0.0144	9.4E-01
U6115_03805		iron transporter	30321.33	29380.47	-0.0454	8.3E-01
U6115_03810		MFS transporter	4699.26	3144.64	-0.5796	2.7E-05
U6115_03815		helix-turn-helix transcriptional regulator	967.25	860.04	-0.1679	2.0E-01
U6115_03820		hypothetical protein	2058.75	1293.62	-0.6706	2.0E-04
U6115_03825	wrbA	NAD(P)H:quinone oxidoreductase	3830.00	2440.52	-0.65	9.1E-11
U6115_03830		YihY family inner membrane protein	3478.60	1892.19	-0.8776	5.7E-11
U6115_03835		DUF3025 domain-containing protein	1393.24	1110.67	-0.3259	3.8E-02
U6115_03840		diguanylate cyclase	3234.88	2980.73	-0.1176	4.0E-01
U6115_03845	cysG	siroheme synthase CysG	6606.89	5096.38	-0.3745	4.6E-03
U6115_03850		PTS sugar transporter subunit IIA	5087.35	5570.42	0.1309	3.8E-01
U6115_03855		HPr family phosphocarrier protein	4252.90	5099.02	0.2617	6.7E-02
U6115_03860	ptsP	phosphoenolpyruvate-protein phosphotransferase	8827.13	8948.55	0.0197	8.7E-01
U6115_03865	rfaQ	putative lipopolysaccharide heptosyltransferase III	10248.33	6753.71	-0.6015	2.9E-07
U6115_03870	msbA	lipid A export permease/ATP-binding protein MsbA	15807.79	8770.20	-0.8499	8.7E-13
U6115_03875		glycosyltransferase family 2 protein	11314.01	5082.83	-1.1545	2.8E-14
U6115_03880		glycosyltransferase family 2 protein	12479.52	5812.33	-1.1022	6.5E-25

U6115_03885		glycosyltransferase family 4 protein	12663.39	6233.33	-1.0225	1.3E-21
U6115_03890		glycosyltransferase	8569.52	4608.64	-0.8949	2.8E-07
U6115_03895		O-antigen ligase family protein	26565.48	25613.50	-0.0527	8.1E-01
U6115_03900		tetratricopeptide repeat protein	7493.18	9275.33	0.3079	7.0E-02
U6115_03905	aroB	3-dehydroquinate synthase	13866.01	12561.37	-0.1426	2.9E-01
U6115_03910	aroK	shikimate kinase AroK	11592.15	7182.72	-0.6904	8.2E-06
U6115_03915	pilQ	type IV pilus secretin PilQ	6998.76	10252.38	0.5507	7.9E-06
U6115_03920		pilus assembly protein PilP	1109.18	1372.84	0.307	1.7E-02
U6115_03925		type 4a pilus biogenesis protein PilO	1388.99	1621.14	0.2228	1.7E-01
U6115_03930		PilN domain-containing protein	1065.59	1285.22	0.2695	4.7E-02
U6115_03935		pilus assembly protein PilM	6698.93	6461.24	-0.0522	7.6E-01
U6115_03940		penicillin-binding protein 1A	35595.05	23054.22	-0.6266	6.2E-10
U6115_03945		hypothetical protein	6390.49	5343.53	-0.2582	1.8E-01
U6115_03950		helix-turn-helix domain-containing protein	1442.04	1691.87	0.2312	1.9E-01
U6115_03955		VUT family protein	1868.99	2006.72	0.1034	5.4E-01
U6115_03960		metallophosphoesterase	1950.46	2773.96	0.509	6.1E-04
U6115_03965		hypothetical protein	2627.95	3125.36	0.2503	2.6E-01
U6115_03970	lpxO	lipid A hydroxylase LpxO	10953.81	10578.23	-0.0503	8.0E-01
U6115_03975		LysR family transcriptional regulator	1848.37	1994.32	0.1093	3.9E-01
U6115_03980		zinc-binding alcohol dehydrogenase family protein	879.26	11674.85	3.7306	2.5E-80
U6115_03985		nuclear transport factor 2 family protein	426.80	10029.22	4.5549	5.3E-71
U6115_03990	cynR	transcriptional regulator CynR	645.43	2503.60	1.9571	1.1E-24
U6115_03995		carbonic anhydrase	329.86	453.74	0.4549	7.9E-03
U6115_04000	cynS	cyanase	519.43	2231.91	2.1032	4.1E-37
U6115_04005		MFS transporter	637.50	733.90	0.2021	2.1E-01
U6115_04010		LysR family transcriptional regulator	625.69	559.19	-0.1623	3.2E-01
U6115_04015		NAD-dependent epimerase/dehydratase family protein	397.08	666.18	0.7493	5.2E-07
U6115_04020		hypothetical protein	229.24	247.70	0.1133	5.3E-01
U6115_04025		lysozyme inhibitor LprI family protein	402.82	417.86	0.0528	7.5E-01
U6115_04030		LysR family transcriptional regulator	1363.86	1073.08	-0.3452	2.5E-02
U6115_04035		MFS transporter	441.78	766.90	0.7957	1.8E-08
U6115_04040		diguanylate cyclase	1866.12	1876.20	0.0083	9.5E-01
U6115_04045		hypothetical protein	217.79	321.42	0.5574	7.5E-03

U6115_04050		AlpA family transcriptional regulator	149.33	197.74	0.4041	7.7E-02
U6115_04055		hypothetical protein	5318.03	2911.70	-0.8691	1.0E-03
U6115_04060		integrase arm-type DNA-binding domain-containing protein	2763.20	2224.45	-0.3129	5.1E-02
U6115_04065		tRNA-Arg	2726.93	673.31	-2.0169	3.5E-21
U6115_04070	ispB	octaprenyl diphosphate synthase	24232.66	11356.47	-1.0933	5.8E-22
U6115_04075	rplU	50S ribosomal protein L21	110069.07	31765.42	-1.7929	3.6E-28
U6115_04080	rpmA	50S ribosomal protein L27	26664.71	8834.40	-1.5938	7.7E-27
U6115_04085	obgE	GTPase ObgE	24026.17	12479.07	-0.9452	6.8E-10
U6115_04090		ABC transporter substrate-binding protein	18457.15	49746.87	1.4305	8.8E-23
U6115_04095	hisQ	histidine ABC transporter permease HisQ	5120.21	5955.82	0.2181	1.1E-01
U6115_04100		ABC transporter permease	25123.78	20265.67	-0.3099	2.1E-02
U6115_04105		ATP-binding cassette domain-containing protein	11180.29	9390.79	-0.2516	2.0E-02
U6115_04110		DUF333 domain-containing protein	1660.69	2408.83	0.5364	3.3E-04
U6115_04115		inhibitor of vertebrate lysozyme family protein	3123.58	6252.26	1.0011	7.8E-06
U6115_04120		succinylglutamate desuccinylase/aspartoacylase family protein	3410.71	4054.78	0.2491	5.8E-02
U6115_04125		hypothetical protein	5571.25	3673.84	-0.6007	6.9E-06
U6115_04130		hypothetical protein	2083.52	1383.60	-0.5904	7.2E-04
U6115_04135		AAA family ATPase	8041.17	5688.23	-0.4994	5.7E-04
U6115_04140		alpha/beta hydrolase	863.49	861.79	-0.004	9.8E-01
U6115_04145		MarR family transcriptional regulator	511.63	932.70	0.8633	1.5E-10
U6115_04150		superoxide dismutase	1676.24	4719.83	1.4933	1.8E-26
U6115_04155		DUF1842 domain-containing protein	3578.32	76174.05	4.4121	1.5E-93
U6115_04160	tpx	thiol peroxidase	24008.82	26930.29	0.1658	1.6E-01
U6115_04165		APC family permease	17416.42	12513.29	-0.477	5.6E-04
U6115_04170		methyl-accepting chemotaxis protein	3714.53	2673.17	-0.4741	2.5E-03
U6115_04175		RDD family protein	1552.86	1858.38	0.259	1.1E-01
U6115_04180		gamma carbonic anhydrase family protein	1605.83	1718.84	0.0977	4.9E-01
U6115_04185		M3 family metallopeptidase	15335.85	29676.79	0.9524	9.5E-07
U6115_04190	xth	exodeoxyribonuclease III	7992.34	6157.52	-0.3763	2.2E-04
U6115_04195	rquA	rhodoquinone biosynthesis methyltransferase RquA	8395.75	5245.66	-0.6786	5.0E-10
U6115_04200		hypothetical protein	86750.12	90544.83	0.0617	6.9E-01

U6115_04205		ferredoxin-NADP reductase	48366.01	22851.26	-1.0817	4.9E-11
U6115_04210		glycosyltransferase family 1 protein	614.18	2278.93	1.8905	2.4E-33
U6115_04215		UDP-2,3-diacylglucosamine diphosphatase	816.52	832.79	0.0284	8.8E-01
U6115_04220		5-carboxymethyl-2-hydroxymuconate Delta-isomerase	2980.49	1907.14	-0.6448	1.2E-06
U6115_04225		hypothetical protein	1573.31	894.30	-0.8145	1.1E-08
U6115_04230		MbnH family di-heme enzyme	6328.62	5545.65	-0.1904	1.7E-01
U6115_04235		MbnP family copper-binding protein	7381.94	7719.89	0.0646	6.9E-01
U6115_04240		TonB-dependent copper receptor	15018.70	13513.29	-0.1523	3.5E-01
U6115_04245		ATP-binding protein	2210.00	2401.09	0.1203	5.7E-01
U6115_04250		response regulator	795.87	1252.27	0.6544	4.1E-05
U6115_04255		3-(methylthio)propionyl-CoA ligase	2657.00	7350.65	1.4682	6.8E-13
U6115_04260		methyl-accepting chemotaxis protein	18509.61	19404.70	0.0681	6.5E-01
U6115_04265		tetratricopeptide repeat protein	3731.89	4852.39	0.3787	8.1E-03
U6115_04270		FAD-binding oxidoreductase	2441.74	3167.61	0.3753	2.1E-02
U6115_04275		M15 family metallopeptidase	834.77	1050.99	0.3322	4.4E-02
U6115_04280		cupin domain-containing protein	719.19	1010.70	0.4891	1.8E-03
U6115_04285		glutathione S-transferase family protein	1674.34	1814.16	0.1154	5.0E-01
U6115_04290		cation/acetate symporter ActP	1376.41	1266.03	-0.1206	6.0E-01
U6115_04295		hypothetical protein	148.60	151.03	0.018	9.4E-01
U6115_04300		GNAT family N-acetyltransferase	431.60	545.77	0.3378	1.0E-01
U6115_04305		hypothetical protein	1557.22	1828.17	0.2313	5.7E-02
U6115_04310		phospholipase C. phosphocholine-specific	3318.33	6061.70	0.8691	8.6E-10
U6115_04315		cold-shock protein	61731.17	26785.74	-1.2045	1.4E-05
U6115_04320	dnaE	DNA polymerase III subunit alpha	30986.45	24639.93	-0.3307	4.9E-04
U6115_04325		flavin reductase family protein	2185.07	3204.14	0.5522	6.8E-04
U6115_04330		VirK/YbjX family protein	9175.12	8466.59	-0.1158	3.2E-01
U6115_04335		PhaM family polyhydroxyalkanoate granule multifunctional regulatory protein	2223.15	1848.20	-0.267	4.8E-02
U6115_04340		NADP-dependent malic enzyme	71089.59	86521.58	0.2834	2.6E-02
U6115_04345		TRAP transporter large permease subunit	1835.88	2330.99	0.3456	6.5E-03
U6115_04350		TRAP transporter small permease	846.10	1148.70	0.4418	5.3E-03
U6115_04355		TRAP transporter substrate-binding protein	4361.55	5971.02	0.4534	5.8E-02
U6115_04360		PAS domain-containing sensor histidine kinase	1732.93	1830.32	0.0794	6.3E-01

U6115_04365		tRNA-Ala	94.96	59.65	-0.6643	3.0E-02
U6115_04370		hypothetical protein	6206.90	4270.21	-0.5396	2.0E-03
U6115_04375		hypothetical protein	1351.42	874.35	-0.6281	4.9E-03
U6115_04380		hypothetical protein	6854.40	4733.37	-0.5341	1.3E-02
U6115_04385		TniQ family protein	4184.51	4073.65	-0.0388	7.7E-01
U6115_04390		TniB family NTP-binding protein	1024.06	1165.80	0.1867	2.0E-01
U6115_04395		DDE-type integrase/transposase/recombinase	3755.18	3255.87	-0.2061	5.8E-02
U6115_04400		hypothetical protein	3722.68	2326.70	-0.6781	9.5E-11
U6115_04405		transposase	490.70	217.10	-1.177	1.0E-07
U6115_04410		IS3 family transposase	165.69	98.61	-0.7413	4.6E-03
U6115_04415		MAC/perforin domain-containing protein	4959.29	4914.18	-0.0132	9.5E-01
U6115_04420		hypothetical protein	3671.00	5427.02	0.5641	1.3E-04
U6115_04425		IS5 family transposase	2116.58	2119.60	0.0023	9.9E-01
U6115_04430	groL	chaperonin GroEL	3949.08	36691.71	3.2159	3.9E-44
U6115_04435		co-chaperone GroES	1019.74	10487.28	3.3627	2.3E-45
U6115_04440		diguanylate cyclase	631.98	668.44	0.0817	6.6E-01
U6115_04445		TIGR02450 family Trp-rich protein	94.24	66.31	-0.5154	8.8E-02
U6115_04450		lipocalin family protein	220.74	134.49	-0.7148	5.4E-04
U6115_04455		cryptochrome/photolyase family protein	815.11	594.15	-0.4562	1.7E-03
U6115_04460		DUF2256 domain-containing protein	19.96	14.49	-0.4955	4.2E-01
U6115_04465		nuclear transport factor 2 family protein	258.10	172.17	-0.5865	1.7E-03
U6115_04470		SDR family NAD(P)-dependent oxidoreductase	363.65	326.30	-0.157	3.9E-01
U6115_04475		DUF3833 domain-containing protein	216.77	148.02	-0.5542	6.0E-03
U6115_04480		cyclopropane-fatty-acyl-phospholipid synthase family protein	1389.86	1140.99	-0.285	4.7E-02
U6115_04485		DUF1365 domain-containing protein	620.08	376.11	-0.7225	4.4E-05
U6115_04490		FAD-dependent oxidoreductase	2215.03	1001.69	-1.1453	8.8E-08
U6115_04495		helix-turn-helix domain-containing protein	1287.18	814.63	-0.6619	2.3E-07
U6115_04500		hypothetical protein	331.70	168.26	-0.9772	1.6E-07
U6115_04505		ATP-binding cassette domain-containing protein	3177.86	1426.07	-1.1558	7.1E-17
U6115_04510		amidase	6940.25	4968.00	-0.4824	1.1E-04
U6115_04515		LysR family transcriptional regulator	5583.83	2882.44	-0.9538	1.3E-13
U6115_04520	aguA	agmatine deiminase	14038.09	6976.21	-1.0087	5.9E-14

U6115_04525		amidohydrolase family protein	1041.60	946.01	-0.141	3.6E-01
U6115_04530		LysR substrate-binding domain-containing protein	384.95	252.40	-0.6093	2.8E-03
U6115_04535		hypothetical protein	1390.02	913.44	-0.6056	1.5E-07
U6115_04540		c-type cytochrome	464.72	617.02	0.4049	4.5E-03
U6115_04545		NADH:flavin oxidoreductase/NADH oxidase family protein	935.17	1278.06	0.4506	7.7E-05
U6115_04550		MerR family transcriptional regulator	979.01	1048.43	0.099	5.1E-01
U6115_04555		penicillin acylase family protein	835.69	2374.82	1.5061	1.2E-15
U6115_04560		chitosanase	355.93	1082.17	1.6043	2.7E-20
U6115_04565		CHAD domain-containing protein	209.99	257.49	0.2893	1.8E-01
U6115_04570		SDR family oxidoreductase	352.38	375.07	0.0877	6.2E-01
U6115_04575		voltage-gated chloride channel family protein	3272.26	1999.92	-0.7101	1.2E-08
U6115_04580		hypothetical protein	1460.98	2167.67	0.5698	5.6E-06
U6115_04585		ferritin-like protein	690.86	712.16	0.0456	7.5E-01
U6115_04590	nadB	L-aspartate oxidase	7347.24	5363.55	-0.4539	2.6E-04
U6115_04595		16S ribosomal RNA	0.00	0.00		
U6115_04600		tRNA-Ile	0.00	0.00		
U6115_04605		tRNA-Ala	0.00	0.00		
U6115_04610		23S ribosomal RNA	0.00	0.00		
U6115_04615	rrf	5S ribosomal RNA	0.00	0.00		
U6115_04620		MarR family transcriptional regulator	3421.33	11785.73	1.7846	3.4E-27
U6115_04625		hypothetical protein	364.63	352.39	-0.0525	8.1E-01
U6115_04630		hypothetical protein	463.24	90275.91	7.6056	0.0E+00
U6115_04635		AfsA-related hotdog domain-containing protein	227.75	111013.08	8.9269	1.5E-284
U6115_04640		HAD-IB family hydrolase	80.45	46715.15	9.1777	8.6E-270
U6115_04645		DUF2165 family protein	81.60	29104.70	8.4833	5.0E-223
U6115_04650		MarR family transcriptional regulator	82.29	27891.04	8.3998	4.0E-236
U6115_04655		efflux transporter outer membrane subunit	169.53	51325.66	8.2401	3.1E-219
U6115_04660		HlyD family efflux transporter periplasmic adaptor subunit	241.02	78278.93	8.344	1.1E-229
U6115_04665		DHA2 family efflux MFS transporter permease subunit	505.29	75629.69	7.2257	1.2E-166
U6115_04670	gabD	NADP-dependent succinate-semialdehyde dehydrogenase	6078.57	39457.66	2.6985	1.2E-132
U6115_04675	gabT	4-aminobutyrate-2-oxoglutarate transaminase	7314.17	51040.84	2.8028	8.8E-128

U6115_04680		TolC family outer membrane protein	2817.53	3450.55	0.2924	2.0E-01
U6115_04685		efflux RND transporter permease subunit	3563.66	4627.27	0.3766	9.6E-03
U6115_04690		efflux RND transporter periplasmic adaptor subunit	1347.98	1586.09	0.2345	8.9E-02
U6115_04695		TetR/AcrR family transcriptional regulator	709.00	647.20	-0.1321	5.3E-01
U6115_04700	argB	acetylglutamate kinase	3664.47	2741.76	-0.4184	6.7E-05
U6115_04705		CBS domain-containing protein	1689.43	1565.83	-0.1094	3.8E-01
U6115_04710		IclR family transcriptional regulator	1011.53	1274.18	0.3324	9.9E-03
U6115_04715		acyl-CoA dehydrogenase	2262.38	10158.56	2.1671	7.2E-24
U6115_04720		CaiB/BaiF CoA-transferase family protein	1222.98	3752.76	1.6177	4.1E-28
U6115_04725		electron transfer flavoprotein-ubiquinone oxidoreductase	15995.22	13731.91	-0.2202	9.8E-02
U6115_04730		hypothetical protein	3498.90	3158.74	-0.1472	3.6E-01
U6115_04735		hypothetical protein	7693.86	7723.40	0.0056	9.8E-01
U6115_04740		DUF3820 family protein	1391.38	1294.76	-0.1046	5.3E-01
U6115_04745	ylqF	ribosome biogenesis GTPase YlqF	7859.18	6469.37	-0.2808	2.5E-02
U6115_04750		hypothetical protein	2543.06	2625.52	0.0461	8.0E-01
U6115_04755		DUF3592 domain-containing protein	2487.54	2331.18	-0.0933	5.8E-01
U6115_04760	ruvX	Holliday junction resolvase RuvX	2415.84	2063.83	-0.2267	1.3E-01
U6115_04765		YqgE/AlgH family protein	10501.81	7036.41	-0.5776	3.3E-05
U6115_04770	smc	chromosome segregation protein SMC	6672.67	6099.58	-0.1295	3.0E-01
U6115_04775		MFS transporter	2417.14	2562.23	0.0841	4.5E-01
U6115_04780		energy transducer TonB	7225.58	14197.24	0.9747	3.2E-14
U6115_04785		MarR family transcriptional regulator	20925.33	49705.23	1.2482	2.2E-06
U6115_04790		cell division protein ZipA C-terminal FtsZ-binding domain-containing protein	16222.60	14636.76	-0.1484	3.1E-01
U6115_04795	ligA	NAD-dependent DNA ligase LigA	5828.54	6584.02	0.1758	3.5E-01
U6115_04800		tetratricopeptide repeat-containing glycosyltransferase family protein	6106.93	9997.60	0.711	3.5E-06
U6115_04805	galU	UTP-glucose-1-phosphate uridylyltransferase GalU	9209.13	7380.89	-0.3193	1.2E-02
U6115_04810		hypoxanthine-guanine phosphoribosyltransferase	8587.55	8229.97	-0.0612	6.3E-01
U6115_04815		ABC transporter ATP-binding protein	739.58	17230.47	4.5431	1.4E-145
U6115_04820		iron ABC transporter permease	848.47	11671.63	3.7822	4.5E-140
U6115_04825		ABC transporter substrate-binding protein	732.78	12679.79	4.1139	2.5E-123
U6115_04830		hemin-degrading factor	2186.39	40476.44	4.211	3.4E-120

U6115_04835		TonB-dependent hemoglobin/transferrin/lactoferrin family receptor	4640.67	75458.91	4.0235	8.8E-80
U6115_04840		hemin uptake protein HemP	321.27	2261.46	2.819	7.6E-34
U6115_04845		S-methyl-5'-thioinosine phosphorylase	6427.92	7106.63	0.1448	3.5E-01
U6115_04850	rfbF	glucose-1-phosphate cytidylyltransferase	4377.32	3256.98	-0.4264	3.3E-03
U6115_04855	rfbG	CDP-glucose 4,6-dehydratase	3215.30	2198.44	-0.548	2.4E-04
U6115_04860	rfbH	lipopolysaccharide biosynthesis protein RfbH	5836.52	4120.05	-0.5025	5.2E-06
U6115_04865		D-lyxose/D-mannose family sugar isomerase	6649.72	6108.79	-0.1226	3.7E-01
U6115_04870		thiamine pyrophosphate-binding protein	6423.28	5907.61	-0.1208	3.7E-01
U6115_04875		class I SAM-dependent methyltransferase	2536.84	2096.20	-0.2751	1.7E-01
U6115_04880		NUDIX domain-containing protein	2054.52	1349.79	-0.6058	1.1E-04
U6115_04885		radical SAM protein	13977.53	10958.51	-0.3512	2.5E-02
U6115_04890		hypothetical protein	6626.57	6136.65	-0.1109	3.4E-01
U6115_04895		NAD(P)-dependent oxidoreductase	3462.65	2830.57	-0.2906	1.1E-02
U6115_04900		hypothetical protein	9627.94	10160.77	0.0777	5.3E-01
U6115_04905		radical SAM protein	8669.15	12002.75	0.4695	4.6E-05
U6115_04910		glycosyltransferase	18412.53	26948.05	0.5495	3.2E-08
U6115_04915		flagellin	133891.04	101914.51	-0.3937	2.6E-02
U6115_04920		flagellin	40338.42	53796.82	0.4154	4.0E-02
U6115_04925		flagellar protein FlaG	12696.85	14829.62	0.224	3.7E-01
U6115_04930	fliD	flagellar filament capping protein FliD	36816.42	40527.12	0.1385	4.5E-01
U6115_04935	fliS	flagellar export chaperone FliS	8040.73	9139.25	0.1848	1.4E-01
U6115_04940	fliT	flagellar protein FliT	6532.37	7344.79	0.1692	3.4E-01
U6115_04945		DUF2802 domain-containing protein	5927.09	5461.34	-0.1178	2.9E-01
U6115_04950		flagellar hook-length control protein FliK	4689.26	4615.01	-0.0231	8.5E-01
U6115_04955		EscU/YscU/HrcU family type III secretion system export apparatus switch protein	1357.13	1244.78	-0.1243	3.1E-01
U6115_04960		NAD(P)-dependent oxidoreductase	3564.86	3813.83	0.0975	4.2E-01
U6115_04965		HU family DNA-binding protein	20179.62	11205.17	-0.8487	5.9E-08
U6115_04970		radical SAM protein	9642.30	8494.37	-0.1828	9.6E-02
U6115_04975		hypothetical protein	654.66	560.40	-0.2274	2.0E-01
U6115_04980		IS3 family transposase	224.15	96.35	-1.2203	5.5E-04
U6115_04985		transposase	325.23	188.36	-0.7888	2.4E-02

U6115_04990		hypothetical protein	4663.91	3127.23	-0.5765	8.0E-04
U6115_04995		chromosome partition protein MukE	1388.53	1063.06	-0.386	2.2E-03
U6115_05000	mukB	chromosome partition protein MukB	10294.09	8787.58	-0.2283	1.1E-01
U6115_05005		Wadjet anti-phage system protein JetD domain-containing protein	4940.57	3947.97	-0.3232	2.5E-02
U6115_05010		YicC/YloC family endoribonuclease	26506.92	17647.91	-0.5869	4.8E-05
U6115_05015		serine/threonine-protein kinase	2078.21	1607.14	-0.3708	5.1E-03
U6115_05020		protein phosphatase 2C domain-containing protein	1537.70	1880.55	0.2905	3.5E-02
U6115_05025	rph	ribonuclease PH	2833.25	2562.84	-0.1444	2.6E-01
U6115_05030		trypsin-like serine protease	3452.27	1648.61	-1.067	8.1E-19
U6115_05035		hypothetical protein	56505.52	39686.76	-0.5097	6.4E-04
U6115_05040	rstB	two-component system sensor histidine kinase RstB	5291.58	3589.68	-0.5595	1.2E-06
U6115_05045	rstA	two-component system response regulator RstA	4560.25	3673.89	-0.3119	3.8E-03
U6115_05050	fdhE	formate dehydrogenase accessory protein FdhE	4741.28	4550.83	-0.0592	6.9E-01
U6115_05055		formate dehydrogenase subunit gamma	3478.80	3605.90	0.0517	7.2E-01
U6115_05060	fdxH	formate dehydrogenase subunit beta	8321.63	8059.99	-0.0463	8.0E-01
U6115_05065	fdnG	formate dehydrogenase-N subunit alpha	40379.76	40162.56	-0.0078	9.6E-01
U6115_05070		efflux transporter outer membrane subunit	271.17	513.98	0.925	9.0E-09
U6115_05075		efflux RND transporter periplasmic adaptor subunit	225.78	594.36	1.3964	5.8E-23
U6115_05080		efflux RND transporter permease subunit	1279.21	1553.98	0.2806	1.7E-02
U6115_05085		hypothetical protein	8735.94	25456.91	1.543	2.6E-23
U6115_05090	purB	adenylosuccinate lyase	39676.34	24130.83	-0.7174	6.1E-07
U6115_05095	ffh	signal recognition particle protein	18997.78	12086.18	-0.6525	5.0E-09
U6115_05100	ccsA	cytochrome c biogenesis protein CcsA	4093.91	3272.40	-0.3226	2.6E-02
U6115_05105		helix-hairpin-helix domain-containing protein	4158.96	20428.80	2.2966	2.5E-57
U6115_05110		porin	5085.22	86187.67	4.0832	3.5E-136
U6115_05115	pilB	type IV-A pilus assembly ATPase PilB	11946.46	10452.23	-0.1927	1.3E-01
U6115_05120		type II secretion system F family protein	5990.92	7811.88	0.383	7.6E-04
U6115_05125		A24 family peptidase	2282.35	3376.45	0.5651	2.4E-04
U6115_05130	coaE	dephospho-CoA kinase	769.40	1084.38	0.4955	2.4E-03
U6115_05135	zapD	cell division protein ZapD	16782.88	12452.07	-0.4305	6.8E-04
U6115_05140	yacG	DNA gyrase inhibitor YacG	1238.07	664.78	-0.8947	8.6E-10
U6115_05145		N-acetylmuramoyl-L-alanine amidase	14247.17	12566.24	-0.1811	1.5E-01

U6115_05150	tsaE	tRNA (adenosine(37)-N6)-threonylcarbamoyltransferase complex ATPase subunit type 1 TsaE	5098.59	3416.25	-0.5773	9.3E-06
U6115_05155	queG	tRNA epoxyqueuosine(34) reductase QueG	2606.85	3071.18	0.2368	1.3E-01
U6115_05160		MaoC family dehydratase	4426.22	7370.97	0.7359	1.8E-04
U6115_05165		electron transfer flavoprotein subunit beta/FixA family protein	23571.26	19625.71	-0.2643	1.5E-02
U6115_05170		electron transfer flavoprotein subunit alpha/FixB family protein	20987.42	19079.05	-0.1375	2.0E-01
U6115_05175		acyl-CoA dehydrogenase	4171.10	7402.21	0.8273	4.6E-14
U6115_05180		type II secretion system protein N	4549.40	2787.33	-0.7062	3.6E-06
U6115_05185	gspD	type II secretion system secretin GspD	7441.90	6174.14	-0.2694	1.1E-01
U6115_05190	gspE	type II secretion system ATPase GspE	3947.79	3377.75	-0.2247	1.3E-01
U6115_05195	gspF	type II secretion system inner membrane protein GspF	2535.78	1931.89	-0.3913	6.6E-03
U6115_05200		hypothetical protein	422.73	263.37	-0.678	5.6E-04
U6115_05205	gspG	type II secretion system major pseudopilin GspG	3421.74	3567.07	0.0601	6.9E-01
U6115_05210		prepilin-type N-terminal cleavage/methylation domain-containing protein	1741.75	1538.93	-0.1782	2.1E-01
U6115_05215	gspI	type II secretion system minor pseudopilin GspI	811.74	713.24	-0.187	2.5E-01
U6115_05220		type II secretion system protein GspJ	612.31	527.77	-0.2145	3.0E-01
U6115_05225	gspK	type II secretion system minor pseudopilin GspK	1107.64	955.36	-0.2122	1.5E-01
U6115_05230	gspL	type II secretion system protein GspL	1032.51	1441.01	0.4817	1.9E-02
U6115_05235		type II secretion system protein M	462.03	554.64	0.2645	1.2E-01
U6115_05240		type II secretion system protein N	1098.41	1267.96	0.2084	1.6E-01
U6115_05245	carA	glutamine-hydrolyzing carbamoyl-phosphate synthase small subunit	18984.14	15980.74	-0.2485	8.0E-02
U6115_05250	leuE	leucine efflux protein LeuE	3026.64	2429.43	-0.3176	1.6E-02
U6115_05255	carB	carbamoyl-phosphate synthase large subunit	33030.13	31111.32	-0.0864	4.8E-01
U6115_05260	greA	transcription elongation factor GreA	9197.04	5396.12	-0.7692	3.0E-07
U6115_05265		DUF4149 domain-containing protein	4832.39	3195.37	-0.5965	1.0E-04
U6115_05270	yhbY	ribosome assembly RNA-binding protein YhbY	10032.32	4800.78	-1.063	8.4E-13
U6115_05275	rlmE	23S rRNA (uridine(2552)-2'-O)-methyltransferase RlmE	12307.20	8408.14	-0.5497	7.1E-03
U6115_05280	ftsH	ATP-dependent zinc metalloprotease FtsH	113722.08	103602.65	-0.1345	2.5E-01
U6115_05285	folP	dihydropteroate synthase	3001.14	2646.91	-0.1818	3.6E-01
U6115_05290	glmM	phosphoglucosamine mutase	14984.77	10060.51	-0.5749	4.8E-08

U6115_05295		ExeM/NucH family extracellular endonuclease	809.07	1007.81	0.3156	6.0E-02
U6115_05300		rhomboid family intramembrane serine protease	569.00	581.91	0.0287	8.6E-01
U6115_05305	alr	alanine racemase	7857.98	10038.95	0.3535	1.2E-03
U6115_05310		DedA family protein	21777.08	16094.42	-0.4362	1.4E-04
U6115_05315		FKBP-type peptidyl-prolyl cis-trans isomerase	6839.75	7307.05	0.0957	5.3E-01
U6115_05320		DUF2189 domain-containing protein	1642.12	1739.38	0.0826	6.4E-01
U6115_05325		D-2-hydroxyacid dehydrogenase	2821.26	1732.48	-0.7027	5.2E-09
U6115_05330		DUF1841 family protein	6694.42	3243.69	-1.0453	9.5E-20
U6115_05335		glutathione peroxidase	1209.55	747.21	-0.693	2.2E-08
U6115_05340	trmB	tRNA (guanosine(46)-N7)-methyltransferase TrmB	9669.98	3610.33	-1.4212	2.3E-23
U6115_05345		SDR family oxidoreductase	3388.47	2543.16	-0.4135	1.2E-03
U6115_05350		CDP-6-deoxy-delta-3,4-glucoseen reductase	9778.15	6275.44	-0.6399	8.6E-07
U6115_05355		DUF3149 domain-containing protein	588.76	925.73	0.6542	2.9E-03
U6115_05360	arcD	arginine-ornithine antiporter	176874.44	149969.03	-0.2381	1.3E-01
U6115_05365		arginine deiminase	85426.69	114821.32	0.4266	2.0E-03
U6115_05370		ornithine carbamoyltransferase	68680.39	101425.64	0.5624	1.1E-03
U6115_05375	arcC	carbamate kinase	22027.60	29851.99	0.4384	1.6E-03
U6115_05380		RebB family R body protein	280.56	748.29	1.4158	3.7E-16
U6115_05385		patatin-like phospholipase family protein	463.95	1265.47	1.4455	5.4E-23
U6115_05390		PadR family transcriptional regulator	450.28	422.81	-0.0928	6.5E-01
U6115_05395		DUF1700 domain-containing protein	1725.83	1891.91	0.1316	3.2E-01
U6115_05400		hypothetical protein	1034.74	1389.13	0.4248	4.8E-03
U6115_05405		hypothetical protein	2734.45	3351.93	0.2937	4.7E-03
U6115_05410		adenine phosphoribosyltransferase	10418.06	4899.85	-1.0882	1.1E-15
U6115_05415		NCS2 family permease	7716.44	6212.37	-0.3125	5.4E-02
U6115_05420	gmk	guanylate kinase	15428.91	6554.79	-1.235	3.2E-20
U6115_05425	rpoZ	DNA-directed RNA polymerase subunit omega	7812.90	5779.09	-0.4351	3.0E-04
U6115_05430		bifunctional (p)ppGpp synthetase/guanosine-3',5'-bis(diphosphate) 3'-pyrophosphohydrolase	29012.11	16619.95	-0.8038	9.7E-12
U6115_05435	thiS	sulfur carrier protein ThiS	1057.43	808.27	-0.3874	2.0E-02
U6115_05440		thiazole synthase	35080.02	20898.33	-0.7472	3.1E-10
U6115_05445	rpsU	30S ribosomal protein S21	42575.29	16888.55	-1.334	2.5E-13
U6115_05450		GatB/YqeY domain-containing protein	11217.20	8792.51	-0.3516	2.3E-02

U6115_05455	dnaG	DNA primase	27964.11	26030.20	-0.1034	5.7E-01
U6115_05460	rpoD	RNA polymerase sigma factor RpoD	85753.29	60694.12	-0.4987	3.5E-04
U6115_05465		tRNA-Ile	189.96	99.33	-0.942	9.7E-06
U6115_05470		tyrosine-type recombinase/integrase	4602.99	2425.41	-0.9244	4.3E-07
U6115_05475		DUF6166 domain-containing protein	4141.74	2198.90	-0.9136	1.2E-04
U6115_05480		hypothetical protein	458.94	305.24	-0.5907	2.5E-02
U6115_05485		replication endonuclease	1540.60	1012.70	-0.6066	1.0E-04
U6115_05490		hypothetical protein	291.60	303.67	0.0574	8.7E-01
U6115_05495		hypothetical protein	480.77	594.81	0.3088	2.3E-01
U6115_05500		hypothetical protein	1334.03	1242.94	-0.1021	5.4E-01
U6115_05505		DUF2523 family protein	282.78	205.00	-0.4622	3.2E-02
U6115_05510		zonular occludens toxin domain-containing protein	1104.98	1012.68	-0.1264	5.1E-01
U6115_05515		hypothetical protein	1960.67	1609.00	-0.2854	3.5E-02
U6115_05520		hypothetical protein	164.56	199.19	0.2728	1.9E-01
U6115_05525		hypothetical protein	1259.64	1079.61	-0.2226	2.0E-01
U6115_05530		retron St85 family RNA-directed DNA polymerase	10999.13	7039.91	-0.6438	7.6E-03
U6115_05535		AAA family ATPase	13846.07	9465.75	-0.5487	2.5E-02
U6115_05540	ptuB	retron Ec78 anti-phage system effector HNH endonuclease PtuB	5188.54	3846.73	-0.4318	4.6E-02
U6115_05545		tRNA-Ile	794.49	769.97	-0.0447	9.0E-01
U6115_05550		IS481 family transposase	229.74	170.51	-0.4301	3.0E-02
U6115_05555		DUF1269 domain-containing protein	847.07	694.10	-0.2873	1.0E-01
U6115_05560		BON domain-containing protein	584.92	538.46	-0.1182	5.3E-01
U6115_05565		SPFH domain-containing protein	299.88	358.25	0.2591	2.2E-01
U6115_05570		hypothetical protein	194.60	199.44	0.0348	8.8E-01
U6115_05575		Crp/Fnr family transcriptional regulator	1873.36	1746.25	-0.1016	4.6E-01
U6115_05580		TraR/DksA family transcriptional regulator	856.44	695.86	-0.299	4.8E-02
U6115_05585		hypothetical protein	638.43	939.22	0.5571	1.6E-04
U6115_05590		YSC84-related protein	579.80	393.36	-0.5599	2.3E-03
U6115_05595		IS3 family transposase	8.88	4.75	-0.8789	3.7E-01
U6115_05600		transposase	791.58	382.99	-1.0481	4.2E-05
U6115_05605		hypothetical protein	566.62	250.70	-1.1765	1.4E-10
U6115_05610		DUF1223 domain-containing protein	365.44	372.40	0.0241	9.2E-01

U6115_05615		hypothetical protein	739.59	861.83	0.2222	1.1E-01
U6115_05620		MltA domain-containing protein	5181.81	7180.21	0.4705	6.1E-05
U6115_05625		BON domain-containing protein	7232.69	12996.28	0.8457	1.1E-03
U6115_05630	lysA	diaminopimelate decarboxylase	6822.39	6309.28	-0.1129	3.7E-01
U6115_05635		LysR family transcriptional regulator	1040.67	1111.98	0.0966	5.2E-01
U6115_05640		hypothetical protein	1250.30	1936.93	0.6323	6.6E-06
U6115_05645	dtpA	dipeptide/tripeptide permease DtpA	13701.82	8369.91	-0.7111	6.2E-11
U6115_05650		alanyl-tRNA editing protein	2472.58	4028.40	0.7043	4.3E-05
U6115_05655		pseudouridine synthase	3344.98	2299.35	-0.5403	6.4E-06
U6115_05660	queF	NADPH-dependent 7-cyano-7-deazaguanine reductase QueF	3080.41	1659.98	-0.8924	1.5E-10
U6115_05665		CPBP family intramembrane glutamic endopeptidase	1574.89	993.61	-0.6643	4.1E-07
U6115_05670	murJ	murein biosynthesis integral membrane protein MurJ	4872.27	2773.70	-0.813	2.9E-10
U6115_05675	rpsT	30S ribosomal protein S20	29486.34	11636.95	-1.3413	9.3E-12
U6115_05680		acetate/propionate family kinase	2715.80	20447.48	2.9123	1.3E-108
U6115_05685		phosphate acetyltransferase	3195.29	33775.69	3.402	5.3E-118
U6115_05690	fabI	enoyl-ACP reductase FabI	3305.11	28432.64	3.1046	1.0E-86
U6115_05695		zinc ribbon domain-containing protein	4860.08	4606.13	-0.0776	5.7E-01
U6115_05700		DUF502 domain-containing protein	8153.23	6447.57	-0.3385	2.4E-02
U6115_05705	aspS	aspartate--tRNA ligase	48693.00	37509.71	-0.3765	5.2E-03
U6115_05710		peroxiredoxin C	177733.47	238965.14	0.4271	2.1E-02
U6115_05715		MFS transporter	1748.28	1650.68	-0.0844	5.8E-01
U6115_05720		arylesterase	1718.90	3121.82	0.8611	5.6E-16
U6115_05725		ABC transporter ATP-binding protein	1400.67	3256.59	1.217	6.2E-22
U6115_05730		FtsX-like permease family protein	2394.66	5165.03	1.1088	7.6E-11
U6115_05735		DUF2946 family protein	810.07	1849.80	1.191	3.6E-10
U6115_05740		MFS transporter	6384.92	5545.05	-0.2035	2.7E-01
U6115_05745		FCD domain-containing protein	9526.76	5578.44	-0.7722	1.5E-05
U6115_05750	fliD	flagellar filament capping protein FliD	975.53	899.34	-0.1185	3.8E-01
U6115_05755		hypothetical protein	9878.19	15485.38	0.6488	5.2E-07
U6115_05760	modB	molybdate ABC transporter permease subunit	3036.63	3499.89	0.2054	1.3E-01
U6115_05765	modA	molybdate ABC transporter substrate-binding protein	5099.23	6316.07	0.3088	1.6E-02
U6115_05770		DUF3313 family protein	6278.41	8238.40	0.3921	7.0E-03

U6115_05775	mltG	endolytic transglycosylase MltG	3647.35	2239.48	-0.7032	5.4E-11
U6115_05780	tmk	dTMP kinase	2968.33	2882.77	-0.0421	7.9E-01
U6115_05785		DNA polymerase III subunit delta'	2372.18	2402.26	0.0181	9.0E-01
U6115_05790		PilZ domain-containing protein	1442.96	1361.22	-0.0846	6.3E-01
U6115_05795		TatD family hydrolase	4942.03	3970.89	-0.3156	2.5E-03
U6115_05800		hypothetical protein	748.81	875.16	0.2251	1.2E-01
U6115_05805		MBL fold metallo-hydrolase	2905.56	3514.09	0.2747	2.9E-02
U6115_05810		CreA family protein	2164.80	2811.81	0.3777	1.0E-03
U6115_05815		chorismate mutase	692.48	796.29	0.2015	1.2E-01
U6115_05820	trpS	tryptophan--tRNA ligase	4376.15	3999.12	-0.1301	3.1E-01
U6115_05825		hypothetical protein	2729.68	5305.55	0.9584	8.5E-10
U6115_05830		translation initiation factor Sui1	198.19	197.06	-0.0067	9.7E-01
U6115_05835		hypothetical protein	1805.04	1533.35	-0.2354	1.1E-01
U6115_05840		pyruvate. water dikinase regulatory protein	8493.59	4674.65	-0.8615	3.1E-07
U6115_05845	ppsA	phosphoenolpyruvate synthase	86651.88	62187.49	-0.4786	2.6E-06
U6115_05850		thiol peroxidase	432.61	491.98	0.1832	2.7E-01
U6115_05855		dicarboxylate/amino acid:cation symporter	3155.53	2333.84	-0.4349	3.2E-04
U6115_05860		MarC family protein	5014.56	2122.81	-1.2397	8.9E-23
U6115_05865		hypothetical protein	3307.13	1461.26	-1.1783	4.9E-18
U6115_05870		hypothetical protein	2247.21	1434.15	-0.6482	2.3E-06
U6115_05875		THUMP domain-containing protein	12483.59	12456.98	-0.0032	9.8E-01
U6115_05880	mutY	A/G-specific adenine glycosylase	5209.56	5816.75	0.1592	2.6E-01
U6115_05885		bifunctional (p)ppGpp synthetase/guanosine-3',5'-bis(diphosphate) 3'-pyrophosphohydrolase	7848.19	8811.97	0.167	1.8E-01
U6115_05890	deoC	deoxyribose-phosphate aldolase	25807.82	14154.90	-0.8666	5.4E-12
U6115_05895	deoA	thymidine phosphorylase	48475.20	35217.32	-0.461	4.3E-03
U6115_05900		phosphopentomutase	43603.21	33521.85	-0.3794	4.7E-03
U6115_05905	deoD	purine-nucleoside phosphorylase	44163.30	26574.92	-0.7328	5.5E-06
U6115_05910	rplM	50S ribosomal protein L13	228274.35	91374.73	-1.3209	4.8E-18
U6115_05915	rpsI	30S ribosomal protein S9	244217.09	102198.45	-1.2568	1.1E-13
U6115_05920	argC	N-acetyl-gamma-glutamyl-phosphate reductase	5936.80	10872.44	0.8729	3.5E-10
U6115_05925	erpA	iron-sulfur cluster insertion protein ErpA	45791.21	53737.67	0.2309	2.1E-01
U6115_05930		CheR family methyltransferase	22233.23	13677.27	-0.701	1.9E-07

U6115_05935		D-amino acid dehydrogenase	4653.72	4860.20	0.0628	6.7E-01
U6115_05940		group II truncated hemoglobin	1928.63	1623.10	-0.2479	1.0E-01
U6115_05945		hypothetical protein	2929.52	2550.33	-0.1992	1.5E-01
U6115_05950	folB	dihydroneopterin aldolase	3371.50	2079.77	-0.6966	4.3E-06
U6115_05955	plsY	glycerol-3-phosphate 1-O-acyltransferase PlsY	2097.97	1719.95	-0.2864	2.6E-02
U6115_05960	lepB	signal peptidase I	3102.95	4774.15	0.6218	5.3E-10
U6115_05965		histone deacetylase family protein	6355.29	6516.97	0.0363	8.1E-01
U6115_05970		AAA family ATPase	786.91	1085.47	0.4662	3.0E-04
U6115_05975		DUF58 domain-containing protein	221.49	432.98	0.9655	6.1E-05
U6115_05980		DUF3488 and transglutaminase-like domain-containing protein	674.16	1065.19	0.6599	1.7E-04
U6115_05985	rpoS	RNA polymerase sigma factor RpoS	11096.13	23057.44	1.0551	3.7E-30
U6115_05990		peptidoglycan DD-metalloendopeptidase family protein	8433.76	14365.70	0.7683	1.0E-11
U6115_05995		protein-L-isoaspartate(D-aspartate) O-methyltransferase	7356.99	6385.06	-0.2045	6.7E-02
U6115_06000	surE	5'/3'-nucleotidase SurE	9221.51	6596.49	-0.4834	5.5E-04
U6115_06005	nadA	quinolinate synthase NadA	16494.91	11900.56	-0.4709	3.0E-03
U6115_06010		bifunctional acetate-CoA ligase family protein/GNAT family N-acetyltransferase	6287.79	9957.42	0.6633	1.5E-10
U6115_06015	rpsP	30S ribosomal protein S16	33263.22	15272.21	-1.1231	1.1E-07
U6115_06020	rimM	ribosome maturation factor RimM	35138.29	15030.17	-1.2251	1.3E-18
U6115_06025	trmD	tRNA (guanosine(37)-N1)-methyltransferase TrmD	67631.48	23808.21	-1.5062	6.7E-40
U6115_06030	rplS	50S ribosomal protein L19	55361.46	18132.42	-1.6104	1.5E-37
U6115_06035		methylated-DNA-[protein]-cysteine S-methyltransferase	454.32	398.95	-0.1896	3.4E-01
U6115_06040	xerD	site-specific tyrosine recombinase XerD	1644.13	1468.55	-0.1634	4.3E-01
U6115_06045	clpA	ATP-dependent Clp protease ATP-binding subunit ClpA	56910.46	95641.14	0.7489	3.9E-08
U6115_06050	clpS	ATP-dependent Clp protease adapter ClpS	10050.62	11133.49	0.1476	4.7E-01
U6115_06055		cold-shock protein	8569.43	14038.27	0.7122	5.7E-03
U6115_06060		NADP-dependent isocitrate dehydrogenase	72189.92	95674.77	0.4063	7.9E-03
U6115_06065		pseudouridine synthase	3648.48	3665.62	0.0069	9.7E-01
U6115_06070		hypothetical protein	32045.06	128209.86	2.0004	8.6E-38
U6115_06075	mutS	DNA mismatch repair protein MutS	9473.01	6540.85	-0.5344	5.0E-08
U6115_06080		glycerate kinase	3320.20	4384.03	0.4013	5.5E-04
U6115_06085		GbsR/MarR family transcriptional regulator	6936.39	17458.55	1.3315	3.3E-19

U6115_06090		cytochrome ubiquinol oxidase subunit I	12129.33	44907.29	1.8883	2.3E-33
U6115_06095		cytochrome d ubiquinol oxidase subunit II	6021.98	27430.51	2.1874	1.7E-23
U6115_06100	ugpB	sn-glycerol-3-phosphate ABC transporter substrate-binding protein UgpB	13798.92	11469.80	-0.2666	2.0E-01
U6115_06105	ugpA	sn-glycerol-3-phosphate ABC transporter permease UgpA	2609.95	1464.09	-0.8334	5.3E-05
U6115_06110	ugpE	sn-glycerol-3-phosphate ABC transporter permease UgpE	1952.83	1089.12	-0.8417	8.7E-04
U6115_06115		sn-glycerol-3-phosphate import ATP-binding protein UgpC	1906.75	1415.64	-0.429	5.0E-02
U6115_06120	ugpQ	glycerophosphodiester phosphodiesterase	1659.10	1775.83	0.0986	6.2E-01
U6115_06125		transporter substrate-binding domain-containing protein	1960.18	1588.62	-0.3027	7.8E-03
U6115_06130		sodium:alanine symporter family protein	6611.32	6370.33	-0.0536	7.2E-01
U6115_06135		methyl-accepting chemotaxis protein	1664.61	1932.55	0.2152	1.9E-01
U6115_06140	hemN	oxygen-independent coproporphyrinogen III oxidase	16348.80	15070.21	-0.1174	3.2E-01
U6115_06145	fnr	fumarate/nitrate reduction transcriptional regulator Fnr	45936.46	36577.54	-0.3287	8.6E-03
U6115_06150		DUF1853 family protein	1384.56	1156.13	-0.2599	8.8E-02
U6115_06155		RNA methyltransferase	2906.28	1716.71	-0.76	4.5E-09
U6115_06160		inositol monophosphatase family protein	24734.47	12598.49	-0.9733	4.2E-14
U6115_06165		multicopper oxidase family protein	2775.47	4913.38	0.8238	9.8E-07
U6115_06170		metal-sensitive transcriptional regulator	1238.88	1309.14	0.0795	6.4E-01
U6115_06175		hypothetical protein	2111.92	1826.27	-0.2102	1.1E-01
U6115_06180		hypothetical protein	3447.66	3996.34	0.2128	2.3E-01
U6115_06185	rpsF	30S ribosomal protein S6	65222.23	21797.41	-1.5812	2.2E-12
U6115_06190	priB	primosomal replication protein N	31740.67	9180.65	-1.7897	7.2E-43
U6115_06195	rpsR	30S ribosomal protein S18	57205.39	15600.88	-1.8746	2.2E-12
U6115_06200	rplI	50S ribosomal protein L9	123291.81	34853.81	-1.8227	8.9E-27
U6115_06205		MarR family transcriptional regulator	639.56	628.33	-0.026	9.1E-01
U6115_06210		anaerobic C4-dicarboxylate transporter	9930.74	8048.45	-0.3031	2.7E-02
U6115_06215		DUF2322 family protein	2489.68	2625.45	0.0773	5.5E-01
U6115_06220		hydrolase	1545.84	3246.79	1.0712	1.7E-13
U6115_06225		hypothetical protein	250.96	466.00	0.8901	2.9E-07
U6115_06230		AraC family transcriptional regulator	563.07	802.81	0.5116	1.0E-02

U6115_06235		GNAT family N-acetyltransferase	917.83	1978.21	1.1085	4.2E-12
U6115_06240		GNAT family N-acetyltransferase	373.68	1156.49	1.6278	5.9E-32
U6115_06245		GNAT family N-acetyltransferase	338.46	1464.60	2.1132	5.7E-53
U6115_06250		hypothetical protein	4504.26	84702.20	4.2331	7.9E-21
U6115_06255		LysR family transcriptional regulator	4654.16	5372.91	0.207	7.7E-02
U6115_06260		hypothetical protein	2599.99	4345.92	0.7407	3.2E-08
U6115_06265		uracil-xanthine permease family protein	6477.76	9753.12	0.5902	1.7E-08
U6115_06270		hypothetical protein	3493.76	4477.98	0.3581	3.6E-03
U6115_06275	upp	uracil phosphoribosyltransferase	14434.83	12074.12	-0.2577	4.9E-02
U6115_06280	grxD	Grx4 family monothiol glutaredoxin	23541.91	14466.68	-0.7024	7.8E-10
U6115_06285		chloride channel protein	9580.04	5988.02	-0.6778	7.2E-08
U6115_06290		RsmB/NOP family class I SAM-dependent RNA methyltransferase	8801.74	4951.09	-0.8301	1.1E-06
U6115_06295		DUF3108 domain-containing protein	4740.83	2585.91	-0.8746	7.9E-13
U6115_06300	purN	phosphoribosylglycinamide formyltransferase	3252.85	2685.41	-0.277	4.4E-02
U6115_06305	purM	phosphoribosylformylglycinamidine cyclo-ligase	19323.33	17750.29	-0.1224	3.4E-01
U6115_06310		hypothetical protein	109.56	99.13	-0.1447	5.4E-01
U6115_06315	hda	DnaA regulatory inactivator Hda	11882.40	8173.44	-0.5398	2.4E-06
U6115_06320		HAD family hydrolase	11767.88	9520.77	-0.3057	3.1E-03
U6115_06325		ATP-binding protein	2635.13	4715.31	0.8391	1.1E-12
U6115_06330		Nudix family hydrolase	2189.32	3742.91	0.7733	9.2E-12
U6115_06335		hypothetical protein	1162.30	3034.33	1.3858	2.4E-28
U6115_06340		hypothetical protein	86277.86	606234.43	2.8128	4.5E-78
U6115_06345	ubiA	4-hydroxybenzoate octaprenyltransferase	3332.74	2380.67	-0.4848	3.2E-05
U6115_06350		chorismate lyase	1331.94	1067.33	-0.3185	5.3E-02
U6115_06355		CidA/LrgA family protein	531.82	809.63	0.6082	1.0E-04
U6115_06360		LrgB family protein	305.89	584.63	0.9331	5.7E-09
U6115_06365		histidine phosphatase family protein	2329.73	5003.08	1.1038	3.5E-09
U6115_06370	metE	5-methyltetrahydropteroyltriglutamate--homocysteine S-methyltransferase	591.18	634.69	0.1012	5.2E-01
U6115_06375		LysR family transcriptional regulator	743.40	972.87	0.3881	1.4E-02
U6115_06380		VOC family protein	720.28	1448.04	1.0071	1.9E-09
U6115_06385		transporter substrate-binding domain-containing protein	1396.29	2869.89	1.0393	6.1E-19

U6115_06390	ntrC	nitrogen regulation protein NR(I)	3921.63	9391.76	1.2597	3.1E-25
U6115_06395	glnL	nitrogen regulation protein NR(II)	3021.86	4393.94	0.5398	2.1E-07
U6115_06400		DUF4124 domain-containing protein	5328.15	5254.25	-0.0202	9.3E-01
U6115_06405	glnA	glutamate-ammonia ligase	158233.43	423260.23	1.4195	2.0E-30
U6115_06410		rhodanese-like domain-containing protein	12388.14	12373.10	-0.0018	9.9E-01
U6115_06415	aroE	shikimate dehydrogenase	19039.87	17879.46	-0.0907	5.3E-01
U6115_06420	mtgA	monofunctional biosynthetic peptidoglycan transglycosylase	3115.74	2333.51	-0.4168	2.3E-03
U6115_06425	mgtE	magnesium transporter	8448.39	4497.90	-0.9094	2.2E-09
U6115_06430	prmC	peptide chain release factor N(5)-glutamine methyltransferase	2047.94	1591.93	-0.3632	4.4E-02
U6115_06435		phosphatase PAP2 family protein	1980.73	1552.41	-0.3512	7.2E-02
U6115_06440		peptidylprolyl isomerase	20245.96	11505.76	-0.8152	3.1E-12
U6115_06445		cupin domain-containing protein	7000.95	6178.10	-0.1803	1.3E-01
U6115_06450	bamC	outer membrane protein assembly factor BamC	40111.65	30614.13	-0.3898	7.5E-04
U6115_06455	dapA	4-hydroxy-tetrahydrodipicolinate synthase	17891.93	15401.39	-0.2163	2.0E-01
U6115_06460		ABC-F family ATPase	32895.68	18229.74	-0.8516	3.0E-09
U6115_06465	fabG	3-oxoacyl-ACP reductase FabG	16628.72	45526.21	1.4531	7.0E-50
U6115_06470	cysB	HTH-type transcriptional regulator CysB	12894.26	8658.11	-0.5748	4.4E-05
U6115_06475		phosphoadenylyl-sulfate reductase	9504.98	11386.05	0.2605	7.5E-02
U6115_06480		nitrite/sulfite reductase	15765.28	22639.87	0.522	7.5E-04
U6115_06485		DUF934 domain-containing protein	5881.04	5923.84	0.0105	9.5E-01
U6115_06490		OmpA family protein	880140.37	518619.95	-0.7631	7.0E-08
U6115_06495		bifunctional riboflavin kinase/FAD synthetase	18118.23	12319.62	-0.5564	3.4E-05
U6115_06500	ileS	isoleucine-tRNA ligase	37134.73	35133.33	-0.08	5.0E-01
U6115_06505	lspA	signal peptidase II	3667.17	2916.14	-0.3305	7.5E-03
U6115_06510	ispH	4-hydroxy-3-methylbut-2-enyl diphosphate reductase	7129.07	6333.23	-0.1706	1.4E-01
U6115_06515		hypothetical protein	1637.52	2226.58	0.4436	2.1E-05
U6115_06520	xseA	exodeoxyribonuclease VII large subunit	5552.55	5591.37	0.01	9.4E-01
U6115_06525		O-acetyl-ADP-ribose deacetylase	431.26	513.88	0.2542	1.6E-01
U6115_06530		ion transporter	1899.31	1997.08	0.0721	6.0E-01
U6115_06535	cysK	cysteine synthase A	12768.09	42189.04	1.7244	1.9E-32
U6115_06540		hypothetical protein	2577.06	2436.98	-0.0799	5.1E-01

U6115_06545		YchJ family metal-binding protein	1269.74	1373.56	0.1143	5.8E-01
U6115_06550		histone deacetylase family protein	18508.89	14767.73	-0.3258	5.5E-02
U6115_06555		hypothetical protein	3221.17	3333.54	0.0495	7.7E-01
U6115_06560		glutathione peroxidase	1766.43	1412.61	-0.3217	1.8E-02
U6115_06565	dcd	dCTP deaminase	8327.28	5333.83	-0.6426	2.6E-04
U6115_06570		FeoA family protein	923.41	1438.52	0.6423	3.3E-04
U6115_06575	feoB	ferrous iron transport protein B	6737.56	10275.92	0.6093	2.7E-05
U6115_06580	bfr	bacterioferritin	9724.66	7622.84	-0.3511	9.4E-03
U6115_06585		dihydroorotate oxidase	5203.86	3137.21	-0.7301	1.4E-10
U6115_06590		class III extradiol ring-cleavage dioxygenase	2597.44	2636.34	0.0215	9.0E-01
U6115_06595		hypothetical protein	580.03	1312.54	1.1782	3.7E-07
U6115_06600		catalase	10789.59	75905.56	2.8146	1.2E-48
U6115_06605		recombination-associated protein RdgC	10003.11	5472.47	-0.8701	3.1E-09
U6115_06610		tRNA-Pro	1108.48	611.70	-0.8554	2.3E-06
U6115_06615		tRNA-Pro	1331.66	743.68	-0.8395	2.2E-04
U6115_06620		tRNA-Thr	2367.50	1505.71	-0.6527	2.0E-01
U6115_06625		SPOR domain-containing protein	7598.24	6132.02	-0.3092	4.3E-02
U6115_06630	ndk	nucleoside-diphosphate kinase	39989.35	24732.40	-0.6932	6.4E-08
U6115_06635	rlmN	23S rRNA (adenine(2503)-C(2))-methyltransferase RlmN	14441.27	8180.45	-0.8201	6.0E-15
U6115_06640	pilW	type IV pilus biogenesis/stability protein PilW	10542.25	6093.23	-0.7907	1.9E-09
U6115_06645		helix-turn-helix domain-containing protein	5614.33	6047.03	0.1071	4.0E-01
U6115_06650	ispG	flavodoxin-dependent (E)-4-hydroxy-3-methylbut-2-enyl-diphosphate synthase	12271.25	10717.33	-0.1952	7.7E-02
U6115_06655	hisS	histidine-tRNA ligase	17173.30	20817.13	0.2776	5.8E-03
U6115_06660		tetratricopeptide repeat protein	10211.67	10292.75	0.0113	9.5E-01
U6115_06665	bamB	outer membrane protein assembly factor BamB	9429.60	9492.31	0.0095	9.5E-01
U6115_06670	der	ribosome biogenesis GTPase Der	28024.88	18317.84	-0.6135	1.3E-10
U6115_06675	hfq	RNA chaperone Hfq	28740.16	26584.02	-0.1125	6.7E-01
U6115_06680	hflX	ribosome rescue GTPase HflX	8349.76	8184.07	-0.029	8.8E-01
U6115_06685	hflK	FtsH protease activity modulator HflK	14937.28	20384.57	0.4485	1.2E-03
U6115_06690	hflC	protease modulator HflC	23951.32	23400.16	-0.0336	7.9E-01
U6115_06695		ATP phosphoribosyltransferase regulatory subunit	6114.27	5947.48	-0.0399	8.2E-01
U6115_06700		adenylosuccinate synthase	26518.46	36745.33	0.4705	1.7E-03

U6115_06705		VOC family protein	363.20	430.96	0.2514	1.7E-01
U6115_06710		cytochrome c peroxidase	1585.80	1252.88	-0.3391	7.2E-02
U6115_06715		acid phosphatase	1314.17	1484.15	0.1751	2.2E-01
U6115_06720		DHA2 family efflux MFS transporter permease subunit	3376.03	2390.20	-0.4983	1.1E-05
U6115_06725		tRNA-Leu	1125.03	399.06	-1.4933	1.7E-10
U6115_06730		tRNA-Leu	3078.09	1344.69	-1.1942	3.4E-09
U6115_06735		tRNA-Leu	4807.45	2884.45	-0.7364	1.7E-06
U6115_06740		tRNA-Leu	1171.81	598.72	-0.9675	4.2E-06
U6115_06745	rnr	ribonuclease R	41436.61	26963.28	-0.62	1.9E-08
U6115_06750		16S ribosomal RNA	0.00	0.00		
U6115_06755		tRNA-Ile	0.00	0.00		
U6115_06760		tRNA-Ala	0.00	0.00		
U6115_06765		23S ribosomal RNA	0.00	0.00		
U6115_06770	rrf	5S ribosomal RNA	2.84	3.45	0.2725	8.3E-01
U6115_06775		oxidoreductase	2732.62	2711.60	-0.011	9.5E-01
U6115_06780		hypothetical protein	3966.31	5943.27	0.5836	5.3E-05
U6115_06785		acetoacetate decarboxylase	7808.84	31485.64	2.0115	1.6E-33
U6115_06790		glycine zipper domain-containing protein	2645.94	7407.87	1.4852	9.5E-24
U6115_06795		S53 family peptidase	1848.57	2041.11	0.1425	5.0E-01
U6115_06800	moaA	GTP 3',8-cyclase MoaA	5436.31	5284.99	-0.0407	8.1E-01
U6115_06805		hypothetical protein	3407.96	4115.48	0.272	2.3E-02
U6115_06810	serB	phosphoserine phosphatase SerB	5991.85	4780.18	-0.3259	2.8E-02
U6115_06815		DoxX family protein	449.32	414.32	-0.1202	4.6E-01
U6115_06820		DNA-binding domain-containing protein	479.85	475.93	-0.0128	9.6E-01
U6115_06825		DUF692 domain-containing protein	859.45	1266.34	0.5574	8.7E-05
U6115_06830		DUF2282 domain-containing protein	5542.11	16179.19	1.5458	1.0E-18
U6115_06835		DUF2282 domain-containing protein	845.55	2138.65	1.3381	2.1E-10
U6115_06840	queE	7-carboxy-7-deazaguanine synthase	1177.36	587.53	-1.0021	6.3E-14
U6115_06845		6-carboxytetrahydropterin synthase	471.60	284.90	-0.7292	3.0E-06
U6115_06850		6-carboxytetrahydropterin synthase	628.99	405.33	-0.6359	6.1E-05
U6115_06855		6-carboxytetrahydropterin synthase	357.34	214.89	-0.7372	6.4E-05
U6115_06860	queC	7-cyano-7-deazaguanine synthase QueC	2222.63	1366.36	-0.7018	8.0E-08
U6115_06865		M35 family metallo-endopeptidase	10088.83	12537.04	0.3134	1.5E-01

U6115_06870		SpoIIE family protein phosphatase	3468.76	3567.61	0.0405	7.5E-01
U6115_06875		diguanylate cyclase	818.28	935.37	0.1931	1.6E-01
U6115_06880		response regulator	1535.30	1790.59	0.2221	6.0E-02
U6115_06885		prolyl oligopeptidase family serine peptidase	2646.01	4193.38	0.6645	1.7E-05
U6115_06890		amidase family protein	839.14	1243.02	0.5652	2.5E-04
U6115_06895		TetR/AcrR family transcriptional regulator	1495.13	1965.59	0.3932	3.7E-03
U6115_06900		alkene reductase	20455.29	44570.12	1.1235	4.7E-12
U6115_06905		NADPH-dependent oxidoreductase	11783.77	20275.72	0.7828	4.2E-07
U6115_06910		TetR/AcrR family transcriptional regulator	13380.94	16689.95	0.3189	3.9E-02
U6115_06915		D-serine ammonia-lyase	1626.59	1532.99	-0.0851	5.3E-01
U6115_06920		LysR family transcriptional regulator	657.11	486.44	-0.4323	3.3E-03
U6115_06925		patatin-like phospholipase family protein	1838.07	1376.46	-0.4173	3.3E-04
U6115_06930		GMC family oxidoreductase	958.98	907.20	-0.0795	5.5E-01
U6115_06935		hypothetical protein	613.08	701.54	0.1935	1.7E-01
U6115_06940		hypothetical protein	152.28	137.26	-0.1568	5.1E-01
U6115_06945		nitric-oxide reductase large subunit	1429.92	1018.39	-0.4899	6.1E-03
U6115_06950		GNAT family N-acetyltransferase	128.88	840.42	2.7042	1.7E-63
U6115_06955		hypothetical protein	177.26	426.08	1.2739	1.9E-11
U6115_06960		glycoside hydrolase family 15 protein	1088.32	2789.65	1.3583	6.3E-11
U6115_06965		lytic polysaccharide monooxygenase auxiliary activity family 9 protein	238.48	540.80	1.1838	1.2E-06
U6115_06970	hmpA	NO-inducible flavohemoprotein	3048.34	4079.99	0.4202	2.2E-02
U6115_06975		Rrf2 family transcriptional regulator	1680.29	2077.94	0.3065	2.4E-02
U6115_06980		VOC family protein	761.83	593.83	-0.3578	1.0E-02
U6115_06985		YnfA family protein	225.95	244.35	0.1182	5.2E-01
U6115_06990		DMT family transporter	306.48	308.51	0.0108	9.5E-01
U6115_06995		DMT family transporter	160.45	223.85	0.4753	1.9E-02
U6115_07000		LysR family transcriptional regulator	1085.23	814.10	-0.4159	1.3E-03
U6115_07005		deoxyribodipyrimidine photo-lyase	2006.18	2363.06	0.2356	6.3E-02
U6115_07010		helix-turn-helix transcriptional regulator	979.27	782.99	-0.3222	2.9E-02
U6115_07015		DMT family transporter	1989.51	1462.69	-0.4437	3.7E-05
U6115_07020		hypothetical protein	2124.73	9390.06	2.1445	1.7E-16
U6115_07025		Nramp family divalent metal transporter	2632.12	10323.38	1.9721	1.2E-17

U6115_07030		MgtC/SapB family protein	1291.98	1937.38	0.5848	3.5E-05
U6115_07035		fumarate hydratase	22903.40	20576.10	-0.1546	1.8E-01
U6115_07040		hypothetical protein	16688.78	11229.70	-0.5716	1.2E-02
U6115_07045		class I SAM-dependent methyltransferase	4116.72	4965.49	0.2706	6.1E-02
U6115_07050		DUF2721 domain-containing protein	3059.87	2309.56	-0.4057	1.4E-03
U6115_07055		L.D-transpeptidase	1104.95	1057.84	-0.0632	6.8E-01
U6115_07060	tadA	tRNA adenosine(34) deaminase TadA	2305.59	1953.54	-0.2391	5.8E-02
U6115_07065		hypothetical protein	22.58	29.77	0.3651	4.9E-01
U6115_07070		hypothetical protein	3138.25	4547.76	0.535	1.2E-04
U6115_07075		hypothetical protein	709.96	1345.68	0.9234	1.2E-09
U6115_07080		phage holin family protein	2614.37	6381.33	1.2873	2.7E-19
U6115_07085		DUF883 family protein	12242.98	35240.73	1.5253	1.8E-42
U6115_07090		MFS transporter	2003.19	1323.65	-0.5978	1.1E-07
U6115_07095		hypothetical protein	26.82	19.50	-0.4643	3.0E-01
U6115_07100		diguanylate cyclase	2505.48	1729.14	-0.5343	1.5E-04
U6115_07105		hypothetical protein	2531.08	2201.07	-0.2009	2.7E-01
U6115_07110		3-hydroxybutyrate oligomer hydrolase family protein	5452.88	6329.66	0.2153	9.3E-02
U6115_07115	guaA	glutamine-hydrolyzing GMP synthase	43646.98	32842.28	-0.4104	3.7E-03
U6115_07120		hypothetical protein	4528.17	2333.40	-0.9563	3.0E-10
U6115_07125	smpB	SsrA-binding protein SmpB	17903.92	9404.16	-0.9289	6.8E-10
U6115_07130		type II toxin-antitoxin system RatA family toxin	4093.32	2557.52	-0.6786	5.0E-07
U6115_07135		RnfH family protein	1114.28	1016.39	-0.1328	3.5E-01
U6115_07140		diguanylate cyclase	15785.75	13544.62	-0.2209	3.1E-02
U6115_07145	ftsB	cell division protein FtsB	5112.10	4356.86	-0.2306	2.8E-02
U6115_07150	eno	phosphopyruvate hydratase	64483.46	66229.63	0.0385	8.1E-01
U6115_07155	kdsA	3-deoxy-8-phosphooctulonate synthase	21376.20	12658.48	-0.756	1.4E-07
U6115_07160		CTP synthase	71626.14	41294.19	-0.7946	7.9E-13
U6115_07165	rpmB	50S ribosomal protein L28	72157.33	26396.57	-1.4508	4.1E-16
U6115_07170	rpmG	50S ribosomal protein L33	33779.26	11121.58	-1.6028	4.1E-11
U6115_07175		uracil-DNA glycosylase	3790.71	3452.70	-0.1348	3.7E-01
U6115_07180	rimI	ribosomal protein S18-alanine N-acetyltransferase	1805.62	1279.62	-0.4968	1.1E-03
U6115_07185	tsaB	tRNA (adenosine(37)-N6)-threonylcarbamoyltransferase complex dimerization subunit type 1 TsaB	6495.18	4714.96	-0.4621	3.4E-05

U6115_07190		EAL domain-containing protein	6372.44	6593.83	0.0492	7.4E-01
U6115_07195		chemotaxis protein CheA	62965.77	57891.58	-0.1212	4.5E-01
U6115_07200	cheZ	protein phosphatase CheZ	32331.70	21411.64	-0.5946	1.9E-04
U6115_07205	cheY	chemotaxis response regulator CheY	19270.94	11855.23	-0.7009	3.0E-03
U6115_07210		chemotaxis protein	39116.32	24713.66	-0.6625	4.0E-04
U6115_07215		chemotaxis protein	73464.33	39341.72	-0.901	1.5E-20
U6115_07220		HAMP domain-containing sensor histidine kinase	2493.87	1953.86	-0.3519	6.3E-04
U6115_07225		STAS domain-containing protein	1250.02	1910.89	0.6125	2.5E-03
U6115_07230		STAS domain-containing protein	1204.37	2614.09	1.1183	1.1E-08
U6115_07235		response regulator	607.30	771.29	0.3432	5.0E-02
U6115_07240		chemotaxis protein CheW	815.10	988.99	0.2784	1.4E-01
U6115_07245		chemotaxis protein CheW	159.93	204.09	0.3506	1.8E-01
U6115_07250		methyl-accepting chemotaxis protein	1397.72	1849.88	0.4042	1.5E-03
U6115_07255		CheR family methyltransferase	403.19	477.99	0.244	1.1E-01
U6115_07260		chemotaxis response regulator protein-glutamate methylesterase	1106.42	1110.76	0.0054	9.7E-01
U6115_07265	cheD	chemoreceptor glutamine deamidase CheD	6237.32	4180.16	-0.5773	3.0E-08
U6115_07270	fdxA	ferredoxin FdxA	6712.24	6057.96	-0.1481	3.8E-01
U6115_07275	gluQRS	tRNA glutamyl-Q(34) synthetase GluQRS	3941.73	2063.30	-0.9333	3.4E-13
U6115_07280		amino acid permease	3909.20	1751.21	-1.1584	9.7E-16
U6115_07285	gcvT	glycine cleavage system aminomethyltransferase GcvT	22110.00	7563.01	-1.5477	1.5E-36
U6115_07290	gcvH	glycine cleavage system protein GcvH	18855.85	5576.51	-1.7575	2.0E-26
U6115_07295	gcvP	aminomethyl-transferring glycine dehydrogenase	53863.24	20352.86	-1.4041	2.3E-26
U6115_07300		class I SAM-dependent methyltransferase	362.80	372.54	0.039	8.2E-01
U6115_07305		EAL domain-containing protein	4617.94	5763.58	0.3198	3.0E-02
U6115_07310		ABC transporter substrate-binding protein	6979.49	15608.92	1.1612	1.0E-11
U6115_07315		TetR/AcrR family transcriptional regulator	7486.04	7437.71	-0.0092	9.5E-01
U6115_07320		porin	2357456.43	1742609.26	-0.436	4.4E-02
U6115_07325		DUF2520 domain-containing protein	1114.02	1428.72	0.3589	3.5E-02
U6115_07330	pncB	nicotinate phosphoribosyltransferase	3120.46	2930.28	-0.0904	4.1E-01
U6115_07335		SAM-dependent methyltransferase	1710.07	1461.33	-0.2267	8.3E-02
U6115_07340		Maf family nucleotide pyrophosphatase	1613.38	1280.87	-0.3334	8.0E-03
U6115_07345		YceD family protein	65778.58	22945.81	-1.5194	6.1E-22

U6115_07350	rpmF	50S ribosomal protein L32	34102.83	13927.11	-1.292	6.6E-20
U6115_07355	plsX	phosphate acyltransferase PlsX	57890.80	21391.68	-1.4363	6.3E-23
U6115_07360		beta-ketoacyl-ACP synthase III	25088.54	9798.83	-1.3565	1.6E-23
U6115_07365	fabD	ACP S-malonyltransferase	23753.75	12912.39	-0.8795	9.1E-13
U6115_07370	fabG	3-oxoacyl-ACP reductase FabG	16742.70	8715.16	-0.9421	2.7E-11
U6115_07375	acpP	acyl carrier protein	38996.80	18522.60	-1.0741	3.0E-16
U6115_07380	fabF	beta-ketoacyl-ACP synthase II	47565.81	23929.07	-0.9912	8.7E-16
U6115_07385		aminodeoxychorismate synthase component I	5828.22	4046.31	-0.5265	5.2E-04
U6115_07390	pabC	aminodeoxychorismate lyase	1745.06	1327.65	-0.3943	1.1E-02
U6115_07395		dicarboxylate/amino acid:cation symporter	5670.66	2549.74	-1.1533	4.0E-14
U6115_07400		DUF4936 family protein	581.66	624.34	0.1034	6.1E-01
U6115_07405		prephenate dehydrogenase/arogenate dehydrogenase family protein	1985.59	3440.17	0.793	4.2E-07
U6115_07410		SDR family oxidoreductase	663.16	1156.71	0.8028	2.2E-08
U6115_07415		LysR substrate-binding domain-containing protein	1342.60	1943.88	0.5334	2.7E-05
U6115_07420		MFS transporter	16700.52	25025.96	0.5835	7.4E-04
U6115_07425	mnmA	tRNA 2-thiouridine(34) synthase MnmA	12460.84	10564.80	-0.2381	4.8E-02
U6115_07430		hypothetical protein	6041.17	3683.52	-0.7137	3.9E-06
U6115_07435		NUDIX hydrolase	597.62	726.03	0.2839	4.9E-02
U6115_07440		(2Fe-2S)-binding protein	3114.38	8353.32	1.4241	5.7E-08
U6115_07445	bfr	bacterioferritin	9786.70	10829.75	0.1463	3.0E-01
U6115_07450		YdgA family protein	56516.53	25852.86	-1.1284	6.1E-14
U6115_07455		fatty acid desaturase	20816.90	21090.73	0.0189	9.1E-01
U6115_07460		methyltransferase domain-containing protein	2523.66	15926.20	2.6579	2.0E-50
U6115_07465		cystathionine beta-synthase	3856.44	30529.08	2.9848	3.5E-40
U6115_07470		PLP-dependent aspartate aminotransferase family protein	3530.34	25829.89	2.871	5.6E-47
U6115_07475		RidA family protein	7458.46	13658.39	0.8728	3.4E-09
U6115_07480		hypothetical protein	267.36	460.76	0.7851	3.5E-08
U6115_07485		C39 family peptidase	655.03	985.27	0.5872	1.2E-04
U6115_07490	miaA	tRNA (adenosine(37)-N6)-dimethylallyltransferase MiaA	3794.90	2878.05	-0.3988	3.3E-04
U6115_07495		GNAT family N-acetyltransferase	3778.74	3740.71	-0.0144	9.3E-01
U6115_07500	scpB	SMC-Scp complex subunit ScpB	20334.68	11350.28	-0.8411	4.3E-12

U6115_07505		pseudouridine synthase	20812.88	15898.01	-0.3888	1.3E-03
U6115_07510		NUDIX domain-containing protein	1312.29	1304.21	-0.0103	9.5E-01
U6115_07515	greB	transcription elongation factor GreB	4014.40	3402.87	-0.2382	1.5E-01
U6115_07520		MarR family winged helix-turn-helix transcriptional regulator	2167.97	2539.99	0.2289	1.4E-01
U6115_07525		hypothetical protein	1543.60	2394.15	0.6334	7.8E-03
U6115_07530		M3 family metallopeptidase	4244.66	4737.18	0.158	1.4E-01
U6115_07535		pyridoxamine 5'-phosphate oxidase family protein	2341.90	1489.19	-0.6537	2.4E-09
U6115_07540		hypothetical protein	4282.82	2814.10	-0.606	7.7E-05
U6115_07545		M3 family metallopeptidase	1871.39	6298.23	1.751	4.5E-19
U6115_07550		hypothetical protein	271.96	352.45	0.3726	1.8E-02
U6115_07555		LysR substrate-binding domain-containing protein	10900.92	11020.80	0.0158	9.1E-01
U6115_07560	minE	cell division topological specificity factor MinE	1818.45	2119.47	0.2205	2.1E-01
U6115_07565	minD	septum site-determining protein MinD	14594.58	17588.81	0.2692	2.4E-02
U6115_07570	minC	septum site-determining protein MinC	4885.97	4409.34	-0.1477	2.1E-01
U6115_07575		phasin family protein	105737.22	551480.48	2.3828	4.3E-77
U6115_07580		hypothetical protein	2981.65	4459.72	0.5803	1.9E-05
U6115_07585	ppa	inorganic diphosphatase	31901.75	19226.49	-0.7305	2.8E-07
U6115_07590	coq7	2-polyprenyl-3-methyl-6-methoxy-1,4-benzoquinone monooxygenase	2089.48	1733.49	-0.2694	2.3E-02
U6115_07595	frdA	fumarate reductase (quinol) flavoprotein subunit	10628.50	5708.75	-0.8968	9.3E-13
U6115_07600		succinate dehydrogenase/fumarate reductase iron-sulfur subunit	4476.79	2424.72	-0.8852	1.1E-08
U6115_07605		fumarate reductase subunit C	1600.75	1033.02	-0.6327	3.0E-04
U6115_07610	frdD	fumarate reductase subunit FrdD	2027.31	1365.48	-0.5696	3.6E-05
U6115_07615		hypothetical protein	695.40	471.47	-0.5629	9.7E-04
U6115_07620		hypothetical protein	1539.08	2071.83	0.4282	1.2E-02
U6115_07625		DUF475 domain-containing protein	4317.20	1496.72	-1.5288	7.4E-36
U6115_07630		DUF1615 domain-containing protein	2334.56	2302.55	-0.0202	9.0E-01
U6115_07635		peptidoglycan DD-metalloendopeptidase family protein	2931.08	3509.04	0.2594	2.2E-02
U6115_07640		transporter substrate-binding domain-containing protein	433.89	388.58	-0.1618	3.4E-01
U6115_07645	ettA	energy-dependent translational throttle protein EttA	22887.79	22784.07	-0.0065	9.6E-01
U6115_07650		BLUF domain-containing protein	2533.38	3218.64	0.3458	3.9E-03

U6115_07655		DUF456 family protein	1285.73	1604.48	0.3196	7.7E-03
U6115_07660	ppk1	polyphosphate kinase 1	17794.64	23419.12	0.3962	1.7E-03
U6115_07665		oxidative damage protection protein	17859.97	12555.49	-0.5084	7.0E-04
U6115_07670	argA	amino-acid N-acetyltransferase	3784.09	4103.16	0.1165	3.4E-01
U6115_07675		S41 family peptidase	29099.43	29455.87	0.0175	8.9E-01
U6115_07680		peptidoglycan DD-metalloendopeptidase family protein	14147.51	12567.72	-0.1709	1.9E-01
U6115_07685	gpmI	2.3-bisphosphoglycerate-independent phosphoglycerate mutase	41702.23	38789.42	-0.1045	4.6E-01
U6115_07690		metalloregulator ArsR/SmtB family transcription factor	4657.57	3443.07	-0.4354	1.1E-03
U6115_07695		DUF4442 domain-containing protein	10637.74	5396.62	-0.979	9.9E-14
U6115_07700		MotA/TolQ/ExbB proton channel family protein	9823.63	9406.57	-0.0625	6.3E-01
U6115_07705		biopolymer transporter ExbD	4688.00	5080.54	0.1162	3.8E-01
U6115_07710	lpxK	tetraacyldisaccharide 4'-kinase	1448.98	1584.47	0.1295	4.3E-01
U6115_07715		Trm112 family protein	522.27	332.71	-0.6533	7.7E-05
U6115_07720	kdsB	3-deoxy-manno-octulosonate cytidylyltransferase	4866.15	3690.77	-0.3987	1.8E-02
U6115_07725	adk	adenylate kinase	15825.61	19661.71	0.3132	2.9E-03
U6115_07730		hemolysin III family protein	11441.18	13591.90	0.2485	5.6E-02
U6115_07735		DUF3369 domain-containing protein	6056.06	6800.10	0.1674	1.0E-01
U6115_07740		HAMP domain-containing sensor histidine kinase	2418.43	2412.16	-0.0042	9.7E-01
U6115_07745		DMT family transporter	2111.25	2173.11	0.0421	7.4E-01
U6115_07750		NAD(P)H-hydrate dehydratase	2346.68	4621.47	0.9777	1.0E-08
U6115_07755		flavin prenyltransferase UbiX	1706.84	2569.38	0.5902	5.1E-07
U6115_07760	rapZ	RNase adapter RapZ	5662.89	6493.38	0.1976	1.2E-01
U6115_07765	hprK	HPr(Ser) kinase/phosphatase	5311.22	5350.19	0.0105	9.3E-01
U6115_07770	ptsN	PTS IIA-like nitrogen regulatory protein PtsN	7574.99	7421.49	-0.0297	8.5E-01
U6115_07775	raiA	ribosome-associated translation inhibitor RaiA	92905.18	91108.96	-0.0282	9.1E-01
U6115_07780		RNA polymerase factor sigma-54	35034.93	25826.02	-0.44	5.6E-04
U6115_07785	lptB	LPS export ABC transporter ATP-binding protein	5904.69	4595.44	-0.3619	9.7E-04
U6115_07790	lptA	lipopolysaccharide transport periplasmic protein LptA	6865.17	5133.88	-0.4194	1.0E-03
U6115_07795	lptC	LPS export ABC transporter periplasmic protein LptC	4666.76	3619.79	-0.3666	2.3E-02
U6115_07800		HAD-IIIA family hydrolase	4867.41	4828.14	-0.0118	9.5E-01
U6115_07805		KpsF/GutQ family sugar-phosphate isomerase	10986.52	12131.37	0.1431	1.8E-01
U6115_07810		cation:proton antiporter	7242.48	6129.29	-0.2407	9.6E-02

U6115_07815		AsmA family protein	14283.17	6809.72	-1.0686	8.8E-17
U6115_07820		tRNA-Gly	1356.47	485.53	-1.4792	1.4E-09
U6115_07825		hypothetical protein	726.11	886.20	0.288	1.1E-01
U6115_07830	sgrR	HTH-type transcriptional regulator SgrR	3712.85	3614.80	-0.0386	7.5E-01
U6115_07835		hypothetical protein	114.01	222.11	0.9589	1.0E-05
U6115_07840		glycosyl hydrolase family 18 protein	1958.08	7187.68	1.8761	2.7E-22
U6115_07845		YjbE family putative metal transport protein	493.44	852.27	0.7886	5.7E-05
U6115_07850		divalent metal cation transporter	596.20	851.71	0.5169	4.0E-03
U6115_07855		protease modulator HflC	641.41	831.56	0.3756	9.8E-03
U6115_07860	hflK	FtsH protease activity modulator HflK	1259.91	1459.91	0.2121	2.1E-01
U6115_07865		SPFH domain-containing protein	502.70	772.00	0.6208	2.1E-05
U6115_07870		DUF465 domain-containing protein	2149.11	2889.74	0.4274	1.4E-03
U6115_07875		hypothetical protein	655.99	600.80	-0.1271	5.7E-01
U6115_07880		glycosyltransferase family 4 protein	2362.84	2676.60	0.18	1.2E-01
U6115_07885		transporter substrate-binding domain-containing protein	2448.70	3065.00	0.3241	3.2E-03
U6115_07890		2OG-Fe dioxygenase family protein	598.72	1071.19	0.8385	8.3E-10
U6115_07895		DUF1345 domain-containing protein	3308.97	3638.90	0.1376	2.8E-01
U6115_07900		glutathione S-transferase	17194.38	17150.43	-0.0036	9.7E-01
U6115_07905		SIMPL domain-containing protein	41700.72	52880.87	0.3427	3.8E-02
U6115_07910	aceB	malate synthase A	8707.29	10992.58	0.3364	5.6E-02
U6115_07915		LysR family transcriptional regulator	1230.24	882.77	-0.4783	8.0E-04
U6115_07920	treR	trehalose operon repressor	3459.38	1722.52	-1.0056	6.8E-11
U6115_07925		hypothetical protein	3584.63	2186.10	-0.7134	2.2E-02
U6115_07930	treP	PTS system trehalose-specific EIIBC component	27287.48	6324.16	-2.1094	3.6E-37
U6115_07935	treC	alpha.alpha-phosphotrehalase	35213.57	7948.63	-2.1475	5.8E-51
U6115_07940		carbohydrate porin	9010.04	2327.39	-1.9531	5.1E-38
U6115_07945		hypothetical protein	1171.75	2907.08	1.3112	2.9E-13
U6115_07950		amino acid permease	5289.82	4209.80	-0.3295	8.3E-03
U6115_07955		hypothetical protein	15439.26	31364.03	1.0226	6.6E-23
U6115_07960		hypothetical protein	346.87	566.38	0.7057	1.0E-06
U6115_07965		DegQ family serine endoprotease	20876.89	23939.38	0.1974	1.2E-01
U6115_07970	nth	endonuclease III	2958.65	2475.63	-0.257	3.7E-02

U6115_07975	rsxB	electron transport complex subunit RsxB	2471.08	2498.04	0.016	9.1E-01
U6115_07980		LysR substrate-binding domain-containing protein	3372.15	4481.39	0.4104	1.7E-02
U6115_07985		carboxymuconolactone decarboxylase family protein	373.47	734.61	0.9788	6.1E-10
U6115_07990		tautomerase family protein	339.44	702.65	1.0481	9.5E-12
U6115_07995		DUF4865 family protein	373.18	677.66	0.8596	2.2E-09
U6115_08000		transporter	2482.45	3184.17	0.3595	4.6E-03
U6115_08005	acs	acetate-CoA ligase	8640.89	26257.39	1.6036	1.1E-19
U6115_08010		cation acetate symporter	3440.14	11577.64	1.7513	5.1E-21
U6115_08015		DUF485 domain-containing protein	470.78	1686.15	1.843	3.3E-11
U6115_08020		class I SAM-dependent methyltransferase	552.85	711.91	0.3646	8.9E-03
U6115_08025		hypothetical protein	195.41	248.16	0.3478	9.6E-02
U6115_08030		cytochrome b	2609.24	3821.62	0.5508	8.1E-05
U6115_08035		YceI family protein	4264.07	6901.27	0.6946	1.7E-03
U6115_08040		YceI family protein	14134.13	37151.16	1.3943	1.9E-12
U6115_08045	sph	sphingomyelin phosphodiesterase	300.21	641.07	1.092	8.0E-09
U6115_08050		FAD-dependent oxidoreductase	463.89	7559.13	4.0287	1.9E-82
U6115_08055	vioB	iminophenyl-pyruvate dimer synthase VioB	522.94	8812.35	4.0771	6.0E-95
U6115_08060		NAD(P)/FAD-dependent oxidoreductase	505.23	19786.24	5.293	6.1E-154
U6115_08065		tryptophan hydroxylase	739.89	29065.83	5.2973	2.3E-169
U6115_08070	vioE	violacein biosynthesis enzyme VioE	424.25	15023.60	5.1455	4.6E-171
U6115_08075		heme-binding protein	199.57	1176.74	2.5606	8.0E-41
U6115_08080		iron-containing redox enzyme family protein	275.44	2441.31	3.1521	8.9E-56
U6115_08085		iron-containing redox enzyme family protein	142.96	1580.13	3.4674	8.1E-81
U6115_08090		iron-containing redox enzyme family protein	167.60	1485.12	3.143	3.8E-79
U6115_08095		aminotransferase class III-fold pyridoxal phosphate-dependent enzyme	113.63	828.41	2.8803	1.5E-49
U6115_08100		EamA family transporter	346.77	990.10	1.5129	6.3E-21
U6115_08105		GNAT family N-acetyltransferase	1218.82	1001.65	-0.2821	2.6E-01
U6115_08110		cold-shock protein	8670.06	5454.29	-0.6686	1.9E-01
U6115_08115		EAL domain-containing protein	607.58	1019.77	0.7484	1.9E-10
U6115_08120		PAS domain S-box protein	317.98	380.61	0.2595	1.4E-01
U6115_08125		AraC family transcriptional regulator	352.10	355.47	0.0145	9.3E-01
U6115_08130		collagenase	562.07	838.45	0.5752	4.7E-05

U6115_08135		hypothetical protein	667.36	699.28	0.0663	6.8E-01
U6115_08140		diguanylate cyclase	2818.39	2593.97	-0.1197	4.2E-01
U6115_08145		citrate synthase family protein	1221.96	1475.89	0.2715	7.2E-02
U6115_08150		CoA transferase	382.61	1299.69	1.7642	1.2E-36
U6115_08155		hypothetical protein	589.53	2370.52	2.0089	1.4E-13
U6115_08160		sugar MFS transporter	622.84	1046.78	0.7472	8.4E-06
U6115_08165		transaldolase family protein	509.57	1033.01	1.0202	1.3E-06
U6115_08170		LuxR C-terminal-related transcriptional regulator	777.61	1104.20	0.5059	4.1E-01
U6115_08175		Spy/CpxP family protein refolding chaperone	239.14	319.46	0.4185	1.4E-02
U6115_08180		response regulator transcription factor	1145.67	1495.95	0.3863	4.2E-03
U6115_08185		HAMP domain-containing sensor histidine kinase	1360.80	2055.17	0.5953	8.3E-04
U6115_08190		hypothetical protein	195.48	377.79	0.9496	5.2E-08
U6115_08195		HAD-IIA family hydrolase	2267.76	2436.30	0.1029	4.0E-01
U6115_08200		YbaK/EbsC family protein	556.70	967.88	0.7995	2.2E-07
U6115_08205		LysE family translocator	704.02	1022.28	0.5375	3.4E-03
U6115_08210		LysR family transcriptional regulator	2288.23	3215.48	0.4905	1.5E-03
U6115_08215		LysE family translocator	9929.52	12936.90	0.3818	1.3E-02
U6115_08220		Lrp/AsnC family transcriptional regulator	507.94	427.09	-0.2501	7.4E-02
U6115_08225		BON domain-containing protein	663.58	532.70	-0.316	2.2E-02
U6115_08230		cytochrome b562	86.43	100.74	0.2055	4.6E-01
U6115_08235		cytochrome b/b6 domain-containing protein	435.29	356.63	-0.2825	1.5E-01
U6115_08240		undecaprenyl-diphosphatase	398.06	538.87	0.4394	5.1E-03
U6115_08245		hypothetical protein	558.52	962.52	0.7865	1.7E-08
U6115_08250		hypothetical protein	261.73	458.02	0.805	8.1E-08
U6115_08255		response regulator transcription factor	236.13	394.83	0.7472	1.1E-04
U6115_08260		ATP-binding protein	737.81	823.97	0.1591	2.3E-01
U6115_08265		tyrosinase family protein	6568.58	9509.83	0.5338	1.6E-04
U6115_08270		DUF2182 domain-containing protein	2175.32	3122.00	0.5215	8.2E-07
U6115_08275		hypothetical protein	16433.50	32113.70	0.9666	2.6E-08
U6115_08280		TnsA endonuclease N-terminal domain-containing protein	756.89	681.14	-0.152	5.2E-01
U6115_08285		hypothetical protein	1267.26	1364.67	0.1064	6.9E-01
U6115_08290		hypothetical protein	807.83	643.28	-0.3308	1.2E-01

U6115_08295	msrB	peptide-methionine (R)-S-oxide reductase MsrB	3119.39	4826.17	0.6295	1.1E-02
U6115_08300		histidine phosphatase family protein	5474.33	10416.70	0.9279	2.0E-12
U6115_08305		deoxyguanosinetriphosphate triphosphohydrolase	16052.72	23842.75	0.5707	4.7E-06
U6115_08310		glycine zipper 2TM domain-containing protein	23860.52	41367.66	0.7939	2.0E-05
U6115_08315		lysophospholipid acyltransferase family protein	3678.95	1990.49	-0.8861	3.0E-12
U6115_08320		DUF1415 domain-containing protein	8900.91	3849.96	-1.2093	2.2E-20
U6115_08325		cold-shock protein	408972.33	75567.98	-2.4362	1.2E-34
U6115_08330		HD domain-containing phosphohydrolase	847.38	1164.47	0.4588	8.2E-05
U6115_08335		dienelactone hydrolase family protein	2069.02	4243.42	1.0368	1.0E-19
U6115_08340		HD domain-containing phosphohydrolase	1552.04	1968.27	0.343	7.9E-03
U6115_08345		hypothetical protein	589.19	806.00	0.4516	1.4E-03
U6115_08350		PaaI family thioesterase	780.16	1182.17	0.5989	5.1E-05
U6115_08355		alpha/beta fold hydrolase	3414.81	2874.01	-0.2488	1.2E-01
U6115_08360	mog	molybdopterin adenylyltransferase	4696.39	3493.65	-0.4273	3.3E-04
U6115_08365	yjgA	ribosome biogenesis factor YjgA	10374.25	6260.51	-0.7287	1.6E-08
U6115_08370	pmbA	metalloprotease PmbA	10070.33	9495.03	-0.0848	4.9E-01
U6115_08375	alkB	DNA oxidative demethylase AlkB	1849.00	2336.25	0.3382	5.5E-02
U6115_08380		AlkA N-terminal domain-containing protein	733.57	984.22	0.4246	5.5E-02
U6115_08385		patatin-like phospholipase family protein	525.25	571.66	0.1234	3.7E-01
U6115_08390		disulfide bond formation protein B	3228.14	8680.73	1.4272	4.5E-19
U6115_08395		CHASE2 domain-containing protein	6312.53	6780.44	0.1032	4.9E-01
U6115_08400		FecR domain-containing protein	4093.39	5145.75	0.3303	2.3E-03
U6115_08405		tRNA-Asp	67.03	16.22	-2.0336	4.6E-07
U6115_08410		tRNA-Val	0.00	0.00		
U6115_08415		tRNA-Asp	0.00	0.00		
U6115_08420		tRNA-Val	0.00	0.00		
U6115_08425		tRNA-Asp	0.00	0.00		
U6115_08430		tRNA-Val	0.00	0.00		
U6115_08435		tRNA-Asp	0.00	0.00		
U6115_08440		tRNA-Val	0.00	0.00		
U6115_08445		tRNA-Asp	0.00	0.00		
U6115_08450		tRNA-Val	0.00	0.00		
U6115_08455		tRNA-Asp	0.00	0.00		

U6115_08460		tRNA-Val	19.16	25.51	0.3855	4.3E-01
U6115_08465		acetyl-CoA carboxylase carboxyltransferase subunit alpha	28975.72	31512.96	0.1212	4.0E-01
U6115_08470	tilS	tRNA lysidine(34) synthetase TilS	3320.67	4666.50	0.491	1.5E-02
U6115_08475		toxic anion resistance protein	3358.75	2429.70	-0.4675	1.1E-03
U6115_08480		hypothetical protein	1802.12	1121.21	-0.6848	1.0E-05
U6115_08485		TerD family protein	3908.28	1927.16	-1.0206	1.6E-15
U6115_08490		VWA domain-containing protein	7224.70	3396.52	-1.0891	2.2E-19
U6115_08495		S41 family peptidase	1225.58	2899.26	1.2416	1.0E-10
U6115_08500		TonB-dependent receptor	1526.41	2424.29	0.6677	7.2E-05
U6115_08505		UDP-2,3-diacylglucosamine diphosphatase	5523.42	3551.55	-0.6372	7.4E-09
U6115_08510		peptidylprolyl isomerase	38017.37	25334.34	-0.5856	5.0E-03
U6115_08515		peptidylprolyl isomerase	12741.51	7433.49	-0.7775	1.7E-05
U6115_08520		outer membrane protein transport protein	30795.00	10646.56	-1.5324	1.2E-18
U6115_08525		cysteine dioxygenase family protein	986.66	701.64	-0.4913	3.6E-04
U6115_08530		Lrp/AsnC family transcriptional regulator	1539.39	933.30	-0.7206	1.1E-09
U6115_08535	phhA	phenylalanine 4-monooxygenase	57114.20	46682.43	-0.2909	1.6E-01
U6115_08540		mannose-1-phosphate guanylyltransferase/mannose-6-phosphate isomerase	30273.43	36778.46	0.2808	7.1E-02
U6115_08545	pcp	pyroglutamyl-peptidase I	4336.32	4938.20	0.1876	2.9E-01
U6115_08550		DUF979 domain-containing protein	6344.76	5471.63	-0.2135	2.0E-01
U6115_08555		DUF969 domain-containing protein	2001.20	2532.62	0.3398	7.5E-02
U6115_08560	pxpA	5-oxoprolinase subunit PxpA	736.05	1416.40	0.9442	8.4E-08
U6115_08565		biotin-dependent carboxyltransferase family protein	1175.40	1752.75	0.5764	1.7E-03
U6115_08570	pxpB	5-oxoprolinase subunit PxpB	1277.35	1748.64	0.4535	1.1E-02
U6115_08575		winged helix DNA-binding protein	1877.03	2317.32	0.3047	4.0E-02
U6115_08580		hypothetical protein	3295.21	2921.81	-0.1733	2.3E-01
U6115_08585		transporter substrate-binding domain-containing protein	1174.91	1568.20	0.4169	2.7E-03
U6115_08590		DUF1501 domain-containing protein	557.40	923.68	0.7297	3.3E-07
U6115_08595		DUF1800 domain-containing protein	1104.24	1134.46	0.0386	8.1E-01
U6115_08600		hypothetical protein	1496.89	1168.18	-0.3568	1.3E-02
U6115_08605		thiamine ABC transporter substrate-binding protein	3119.98	2337.48	-0.4171	9.9E-04
U6115_08610		GNAT family protein	757.42	716.59	-0.0794	5.8E-01

U6115_08615		iron ABC transporter permease	627.63	592.20	-0.0839	6.8E-01
U6115_08620		ABC transporter ATP-binding protein	941.32	826.45	-0.1872	3.5E-01
U6115_08625		MarR family transcriptional regulator	1291.64	1102.43	-0.2281	9.7E-02
U6115_08630		VOC family protein	340.31	534.15	0.6495	1.5E-04
U6115_08635		helix-turn-helix transcriptional regulator	650.41	1220.18	0.9074	6.9E-09
U6115_08640		kinase inhibitor	849.63	1619.03	0.9311	8.3E-10
U6115_08645		GNAT family N-acetyltransferase	1226.05	1262.09	0.041	7.9E-01
U6115_08650		serine hydrolase	6047.13	3456.95	-0.8069	1.6E-08
U6115_08655		hypothetical protein	129.23	97.31	-0.41	6.5E-02
U6115_08660	uvrB	excinuclease ABC subunit UvrB	7934.77	6544.81	-0.278	3.7E-02
U6115_08665		bile acid:sodium symporter family protein	1873.39	1150.88	-0.7028	2.6E-07
U6115_08670		LysR family transcriptional regulator	328.66	298.25	-0.1377	4.4E-01
U6115_08675		lipase family protein	256.48	491.75	0.9369	4.3E-07
U6115_08680		alkaline phosphatase family protein	679.50	790.22	0.2199	1.2E-01
U6115_08685		transporter	2633.24	2663.26	0.0172	9.1E-01
U6115_08690		hypothetical protein	259.96	273.38	0.0788	6.9E-01
U6115_08695		low molecular weight protein-tyrosine-phosphatase	2031.41	2483.35	0.2903	9.4E-02
U6115_08700		chemotaxis protein CheB	87120.78	65406.01	-0.4136	1.1E-03
U6115_08705		sigma-54 dependent transcriptional regulator	70702.33	51185.13	-0.466	8.0E-05
U6115_08710	fliE	flagellar hook-basal body complex protein FliE	15357.47	13419.99	-0.1945	2.8E-01
U6115_08715	fliF	flagellar basal-body MS-ring/collar protein FliF	34576.11	25164.14	-0.4583	2.7E-04
U6115_08720	fliG	flagellar motor switch protein FliG	22536.42	17692.48	-0.3491	5.3E-04
U6115_08725		FliH/SctL family protein	9467.65	9258.04	-0.0322	8.4E-01
U6115_08730	fliI	flagellar protein export ATPase FliI	8115.86	8737.75	0.1066	5.5E-01
U6115_08735	fliJ	flagellar export protein FliJ	1778.65	1399.83	-0.3459	5.9E-03
U6115_08740		flagellar hook-length control protein FliK	10871.34	52050.68	2.2594	2.2E-67
U6115_08745		flagellar basal body-associated FliL family protein	10234.60	16847.40	0.7191	7.2E-07
U6115_08750	fliM	flagellar motor switch protein FliM	11223.84	12317.68	0.1342	3.6E-01
U6115_08755	fliN	flagellar motor switch protein FliN	3612.51	4795.45	0.4089	5.0E-03
U6115_08760	fliO	flagellar biosynthetic protein FliO	999.99	1408.59	0.4942	1.0E-02
U6115_08765	fliP	flagellar type III secretion system pore protein FliP	4474.65	5656.36	0.338	8.8E-03
U6115_08770		GNAT family N-acetyltransferase	2035.37	3092.52	0.6034	1.8E-02
U6115_08775	fliQ	flagellar biosynthesis protein FliQ	1052.22	1348.40	0.3565	7.1E-03

U6115_08780	fliR	flagellar biosynthetic protein FliR	3009.43	3219.48	0.0974	5.7E-01
U6115_08785		YebC/PmpR family DNA-binding transcriptional regulator	21172.66	14269.61	-0.5693	1.7E-06
U6115_08790		aminopeptidase P N-terminal domain-containing protein	5066.68	4907.14	-0.0462	7.7E-01
U6115_08795		FAD-dependent oxidoreductase	2272.98	3030.13	0.4145	5.9E-03
U6115_08800		UbiH/UbiF family hydroxylase	6223.39	9497.02	0.6098	2.0E-08
U6115_08805		DsbC family protein	16335.01	23508.16	0.5252	5.5E-04
U6115_08810		hypothetical protein	3170.90	7611.97	1.2632	6.5E-18
U6115_08815		acyl-CoA-binding protein	8880.78	8649.09	-0.0381	8.7E-01
U6115_08820		type IV pilin protein	275.95	287.76	0.0604	8.0E-01
U6115_08825		PilC/PilY family type IV pilus protein	1343.04	1783.08	0.4087	9.2E-02
U6115_08830		PilX N-terminal domain-containing pilus assembly protein	141.26	173.76	0.2961	1.7E-01
U6115_08835		PilW family protein	384.66	455.83	0.244	1.1E-01
U6115_08840	pilV	type IV pilus modification protein PilV	398.92	487.39	0.2897	4.0E-02
U6115_08845		GspH/FimT family protein	1174.35	1066.57	-0.139	4.0E-01
U6115_08850	nhaR	transcriptional activator NhaR	582.00	554.18	-0.0703	7.5E-01
U6115_08855	htpX	protease HtpX	474.09	850.20	0.8403	8.1E-09
U6115_08860		TerC family protein	587.21	759.60	0.3733	1.5E-02
U6115_08865		response regulator	22840.44	33314.36	0.5445	1.7E-04
U6115_08870		ATP-binding protein	18925.12	20954.04	0.1469	3.5E-01
U6115_08875		porin	1176163.99	731141.83	-0.6859	2.0E-05
U6115_08880		FAD-dependent oxidoreductase	4415.15	5245.66	0.2482	1.5E-01
U6115_08885		thiamine pyrophosphate-binding protein	2387.51	3076.10	0.365	3.5E-02
U6115_08890	mgtA	magnesium-translocating P-type ATPase	47488.65	201969.98	2.0885	8.7E-18
U6115_08895		hypothetical protein	12556.77	63034.88	2.3278	1.3E-15
U6115_08900		hypothetical protein	32690.20	126997.04	1.9579	1.9E-15
U6115_08905		hypothetical protein	11120.73	33487.86	1.5905	5.1E-09
U6115_08910		hypothetical protein	604.76	409.98	-0.5613	6.8E-05
U6115_08915	lipA	lipoyl synthase	26833.47	20314.72	-0.4016	1.3E-03
U6115_08920	lipB	lipoyl(octanoyl) transferase LipB	10161.11	6133.91	-0.7284	1.2E-12
U6115_08925		DUF493 domain-containing protein	7173.64	6193.12	-0.2121	3.2E-01
U6115_08930		D-alanyl-D-alanine carboxypeptidase family protein	48274.49	41779.93	-0.2085	9.3E-02

U6115_08935	ilvA	threonine ammonia-lyase. biosynthetic	5172.90	5957.37	0.2036	7.4E-02
U6115_08940		HAD-IA family hydrolase	1432.10	1495.60	0.0635	6.7E-01
U6115_08945		16S ribosomal RNA	0.00	0.00		
U6115_08950		tRNA-Ile	0.00	0.00		
U6115_08955		tRNA-Ala	0.00	0.00		
U6115_08960		23S ribosomal RNA	0.00	0.00		
U6115_08965	rrf	5S ribosomal RNA	34.60	13.74	-1.3492	3.8E-03
U6115_08970		ATP-binding protein	6914.10	5092.38	-0.4413	8.8E-03
U6115_08975		GlxA family transcriptional regulator	8560.98	5329.60	-0.6836	3.4E-09
U6115_08980		ABC transporter permease	6160.94	6109.28	-0.0118	9.3E-01
U6115_08985		ABC transporter permease	7827.47	7187.17	-0.1229	2.4E-01
U6115_08990		ABC transporter substrate-binding protein	28796.12	27444.87	-0.0693	6.7E-01
U6115_08995		hypothetical protein	2450.22	2559.65	0.063	8.0E-01
U6115_09000		NAD-glutamate dehydrogenase	90877.05	117698.53	0.3731	5.3E-03
U6115_09005		bifunctional metallophosphatase/5'-nucleotidase	1998.69	3153.96	0.6579	5.0E-06
U6115_09010		GGDEF domain-containing protein	2611.48	2225.78	-0.2309	8.4E-02
U6115_09015	dut	dUTP diphosphatase	4307.59	3563.30	-0.2737	2.7E-02
U6115_09020	coaBC	bifunctional phosphopantothenoylcysteine decarboxylase/phosphopantothenate--cysteine ligase CoaBC	5820.78	4542.56	-0.3579	3.4E-03
U6115_09025	radC	DNA repair protein RadC	3032.65	4274.70	0.4954	1.9E-06
U6115_09030		hypothetical protein	3295.50	12102.10	1.8762	5.6E-51
U6115_09035		oxidoreductase	4321.17	5051.15	0.2253	3.8E-01
U6115_09040		DUF2844 domain-containing protein	627.73	1065.94	0.7647	4.7E-08
U6115_09045		DUF3443 domain-containing protein	2124.28	3400.55	0.679	2.5E-06
U6115_09050		hypothetical protein	261.11	370.04	0.5074	4.2E-03
U6115_09055		flavin reductase family protein	1070.68	1250.76	0.2252	7.4E-02
U6115_09060	tyrS	tyrosine--tRNA ligase	13401.61	10914.94	-0.296	3.5E-02
U6115_09065		cytochrome b/b6 domain-containing protein	1994.69	3176.13	0.6713	3.4E-09
U6115_09070		cytochrome c	8182.22	9064.21	0.1478	3.0E-01
U6115_09075		cytochrome c	19121.65	18080.75	-0.0807	5.9E-01
U6115_09080		Fur family transcriptional regulator	1353.18	861.68	-0.6509	4.6E-07
U6115_09085		GTP-binding protein	1641.75	1879.16	0.1945	2.6E-01

U6115_09090		hypothetical protein	24.58	29.96	0.2748	5.1E-01
U6115_09095		ABC transporter ATP-binding protein	764.16	905.53	0.245	1.8E-01
U6115_09100		metal ABC transporter permease	1310.16	1276.06	-0.0381	8.5E-01
U6115_09105		metal ABC transporter substrate-binding protein	3030.41	4040.97	0.4147	1.3E-03
U6115_09110		sulfite exporter TauE/SafE family protein	752.24	1074.72	0.5166	2.9E-03
U6115_09115		enoyl-CoA hydratase	1432.69	1259.49	-0.186	3.0E-01
U6115_09120		LysR family transcriptional regulator	877.11	1136.55	0.3745	1.7E-02
U6115_09125		hypothetical protein	537.03	1034.07	0.9475	2.2E-09
U6115_09130		hypothetical protein	224.71	365.49	0.7069	7.6E-05
U6115_09135		GNAT family protein	184.45	262.54	0.5123	1.1E-02
U6115_09140		cytochrome c	7260.43	6182.37	-0.2321	2.9E-02
U6115_09145		FAD/NAD(P)-binding protein	20426.54	15380.31	-0.4094	1.2E-03
U6115_09150		fructose-specific PTS transporter subunit EIIC	8261.68	12304.71	0.5749	6.1E-08
U6115_09155	pfkB	1-phosphofructokinase	3491.98	4820.27	0.4654	3.9E-04
U6115_09160	ptsP	phosphoenolpyruvate-protein phosphotransferase	15635.18	25335.28	0.6964	2.4E-11
U6115_09165		LacI family DNA-binding transcriptional regulator	5563.00	4755.00	-0.2262	1.2E-01
U6115_09170		cytochrome b	12960.42	10251.18	-0.3382	1.8E-02
U6115_09175		nucleoside triphosphate pyrophosphohydrolase family protein	2128.02	1875.70	-0.1818	1.1E-01
U6115_09180	aroA	3-phosphoshikimate 1-carboxyvinyltransferase	5712.43	5541.15	-0.0437	7.3E-01
U6115_09185	cmk	(d)CMP kinase	16173.78	6004.61	-1.4292	1.2E-25
U6115_09190	rpsA	30S ribosomal protein S1	381109.76	131817.34	-1.5317	2.3E-33
U6115_09195		integration host factor subunit beta	41802.80	11467.46	-1.8661	2.8E-28
U6115_09200		LapA family protein	7978.54	6982.34	-0.1922	1.6E-01
U6115_09205	lapB	lipopolysaccharide assembly protein LapB	8614.75	6460.44	-0.4153	6.2E-05
U6115_09210	pyrF	orotidine-5'-phosphate decarboxylase	5732.58	4932.17	-0.2171	4.9E-02
U6115_09215		UDP-glucose/GDP-mannose dehydrogenase family protein	6754.01	6607.53	-0.0318	8.3E-01
U6115_09220	rfaE1	D-glycero-beta-D-manno-heptose-7-phosphate kinase	4413.61	8047.80	0.8664	1.8E-08
U6115_09225		DUF924 family protein	2347.57	4346.94	0.8886	5.5E-15
U6115_09230	rfaD	ADP-glyceromanno-heptose 6-epimerase	18192.04	23432.29	0.3652	3.5E-02
U6115_09235		FCD domain-containing protein	3144.69	2068.02	-0.6048	1.2E-06
U6115_09240	arsB	ACR3 family arsenite efflux transporter	825.66	1034.32	0.3228	1.5E-02

U6115_09245		arsenate reductase ArsC	251.01	317.04	0.3375	3.6E-02
U6115_09250		metalloregulator ArsR/SmtB family transcription factor	432.52	398.77	-0.1177	4.3E-01
U6115_09255		(Fe-S)-binding protein	462.78	962.06	1.0545	2.5E-08
U6115_09260		lactate utilization protein	431.47	1011.15	1.2285	3.7E-14
U6115_09265		LutB/LldF family L-lactate oxidation iron-sulfur protein	533.59	1390.98	1.3821	5.7E-14
U6115_09270		FAD-binding and (Fe-S)-binding domain-containing protein	722.80	2051.89	1.5052	1.3E-17
U6115_09275		lactate permease LctP family transporter	564.03	824.37	0.548	2.1E-04
U6115_09280	cysM	cysteine synthase CysM	4874.01	4618.54	-0.0778	5.8E-01
U6115_09285		transporter substrate-binding domain-containing protein	2091.87	2039.02	-0.0368	8.6E-01
U6115_09290	zapE	cell division protein ZapE	5257.75	4059.27	-0.3731	1.2E-03
U6115_09295		MgtC/SapB family protein	1568.96	1442.47	-0.1209	4.2E-01
U6115_09300	rbsK	ribokinase	919.09	1040.63	0.1769	3.5E-01
U6115_09305	rbsD	D-ribose pyranase	260.19	269.70	0.0505	8.3E-01
U6115_09310		sugar ABC transporter ATP-binding protein	1123.82	1118.69	-0.0079	9.7E-01
U6115_09315	rbsC	ribose ABC transporter permease	947.86	922.70	-0.039	8.2E-01
U6115_09320	rbsB	ribose ABC transporter substrate-binding protein RbsB	3320.47	4331.97	0.3831	2.7E-03
U6115_09325		substrate-binding domain-containing protein	1435.44	1484.26	0.0472	7.4E-01
U6115_09330		hypothetical protein	421.87	489.43	0.213	1.5E-01
U6115_09335		flagellin	17421.97	4911.60	-1.8268	7.5E-21
U6115_09340		hypothetical protein	1275.01	1067.76	-0.2556	4.6E-02
U6115_09345		hybrid sensor histidine kinase/response regulator	3534.27	2875.46	-0.2972	9.9E-02
U6115_09350		3-keto-5-aminohexanoate cleavage protein	735.52	782.95	0.0885	5.5E-01
U6115_09355		helix-turn-helix domain-containing protein	13000.96	9934.71	-0.388	1.0E-03
U6115_09360		flagellar biosynthesis protein FlhA	3933.32	989.77	-1.9912	2.6E-65
U6115_09365		flagellar type III secretion system protein FlhB	1294.02	400.04	-1.6907	1.1E-35
U6115_09370	fliR	flagellar biosynthetic protein FliR	1117.21	383.00	-1.5423	1.3E-28
U6115_09375	fliQ	flagellar biosynthesis protein FliQ	745.49	208.02	-1.8398	1.5E-31
U6115_09380	fliP	flagellar type III secretion system pore protein FliP	1704.60	384.13	-2.1494	5.7E-67
U6115_09385	fliN	flagellar motor switch protein FliN	790.05	238.08	-1.7274	1.7E-29
U6115_09390		FliM/FliN family flagellar motor switch protein	3317.94	572.39	-2.5348	1.4E-60
U6115_09395		flagellar hook-basal body complex protein FliE	1953.50	380.73	-2.3569	7.6E-59

U6115_09400	fliF	flagellar basal-body MS-ring/collar protein FliF	3661.30	809.14	-2.1771	3.0E-59
U6115_09405		flagellar motor switch protein FliG	3218.69	872.91	-1.8826	6.7E-36
U6115_09410	fliH	flagellar assembly protein FliH	1430.66	448.30	-1.6731	4.6E-14
U6115_09415	fliI	flagellar protein export ATPase FliI	2124.24	552.48	-1.9416	5.6E-32
U6115_09420	fliJ	flagellar export protein FliJ	754.72	185.99	-2.0176	2.1E-43
U6115_09425	fliD	flagellar filament capping protein FliD	4541.05	1418.91	-1.6787	3.5E-53
U6115_09430	fliS	flagellar export chaperone FliS	874.78	240.32	-1.8669	7.3E-44
U6115_09435		hypothetical protein	648.03	168.48	-1.9413	4.4E-25
U6115_09440		flagellar hook-length control protein FliK	1442.19	513.78	-1.4896	3.7E-21
U6115_09445		flagellar basal body-associated FliL family protein	751.87	269.57	-1.4809	4.3E-29
U6115_09450		FliA/WhiG family RNA polymerase sigma factor	789.87	212.85	-1.8884	4.4E-24
U6115_09455	motA	flagellar motor stator protein MotA	2246.23	491.43	-2.1908	1.7E-63
U6115_09460		flagellar motor protein MotB	2131.54	1055.57	-1.0128	1.8E-15
U6115_09465		Crp/Fnr family transcriptional regulator	8822.86	13410.65	0.6042	7.9E-05
U6115_09470	flgM	flagellar biosynthesis anti-sigma factor FlgM	1995.29	2125.38	0.0922	5.1E-01
U6115_09475	flgN	flagellar export chaperone FlgN	975.93	1604.07	0.7173	1.0E-04
U6115_09480	flgJ	flagellar assembly peptidoglycan hydrolase FlgJ	2160.05	3372.94	0.6432	4.3E-04
U6115_09485		tRNA-Ala	335.53	56.01	-2.5711	5.0E-19
U6115_09490		tRNA-Ala	807.02	141.48	-2.5068	7.5E-18
U6115_09495		tRNA-Glu	3.54	7.75	1.1563	2.4E-01
U6115_09500		tRNA-Ala	116.64	37.39	-1.6284	1.3E-06
U6115_09505		amino acid ABC transporter substrate-binding protein	3663.76	13080.62	1.836	7.1E-15
U6115_09510		amino acid ABC transporter permease	1290.37	3775.23	1.5486	1.3E-22
U6115_09515		amino acid ABC transporter ATP-binding protein	905.09	2710.43	1.5815	2.1E-29
U6115_09520		diguanylate cyclase	782.94	1930.70	1.3012	1.4E-18
U6115_09525		PAS domain S-box protein	1491.75	2827.63	0.9221	3.0E-09
U6115_09530		CHASE domain-containing protein	940.12	666.31	-0.4979	1.9E-03
U6115_09535		tRNA-Ala	1.27	1.21	-0.1356	9.5E-01
U6115_09540	hpnC	squalene synthase HpnC	2413.48	2549.73	0.0799	5.9E-01
U6115_09545	hpnD	presqualene diphosphate synthase HpnD	2184.14	3136.07	0.5215	1.2E-04
U6115_09550	hpnE	hydroxysqualene dehydroxylase HpnE	2592.88	3372.26	0.3794	4.6E-02
U6115_09555		SDR family oxidoreductase	2988.88	3743.93	0.3252	5.5E-03
U6115_09560		hypothetical protein	1893.28	1968.62	0.0565	6.4E-01

U6115_09565	radA	DNA repair protein RadA	3931.49	3141.03	-0.3239	5.9E-03
U6115_09570		sigma-70 family RNA polymerase sigma factor	1083.73	1021.29	-0.0867	5.1E-01
U6115_09575		zf-HC2 domain-containing protein	96.06	135.13	0.4947	3.0E-02
U6115_09580		hypothetical protein	353.47	955.93	1.438	2.5E-25
U6115_09585	rlmF	23S rRNA (adenine(1618)-N(6))-methyltransferase RlmF	1116.51	2173.38	0.9627	9.4E-12
U6115_09590		DUF2325 domain-containing protein	1648.40	21492.11	3.7058	4.5E-56
U6115_09595		transporter substrate-binding domain-containing protein	217.53	282.61	0.3793	5.4E-02
U6115_09600		hypothetical protein	599.56	1007.85	0.7512	2.2E-08
U6115_09605		AarF/ABC1/UbiB kinase family protein	2103.22	3205.89	0.6089	1.5E-08
U6115_09610		C40 family peptidase	12178.88	8922.55	-0.4488	1.7E-02
U6115_09615		hypothetical protein	2766.69	3247.23	0.2317	6.4E-02
U6115_09620		lipoprotein-releasing ABC transporter permease subunit	8034.86	6975.77	-0.2039	7.6E-02
U6115_09625	lolD	lipoprotein-releasing ABC transporter ATP-binding protein LolD	4202.84	5178.08	0.3011	3.2E-02
U6115_09630		mechanosensitive ion channel domain-containing protein	8199.23	8789.59	0.1003	4.6E-01
U6115_09635		response regulator	2917.17	3822.92	0.3902	9.9E-03
U6115_09640	nudB	dihydroneopterin triphosphate diphosphatase	1935.13	2665.90	0.4626	1.6E-03
U6115_09645		cupredoxin family copper-binding protein	119.39	179.97	0.5879	3.2E-03
U6115_09650		metallophosphoesterase	548.78	742.58	0.4352	3.3E-03
U6115_09655		MFS transporter	1672.55	1837.62	0.1344	3.0E-01
U6115_09660		gluconokinase	801.59	681.72	-0.2321	9.3E-02
U6115_09665		LacI family DNA-binding transcriptional regulator	1231.52	1366.10	0.15	3.8E-01
U6115_09670		FMN-binding negative transcriptional regulator	307.75	601.01	0.967	3.0E-11
U6115_09675		hypothetical protein	448.34	771.61	0.7838	4.1E-08
U6115_09680		PAS domain S-box protein	1214.49	1872.48	0.6247	2.1E-05
U6115_09685		GNAT family N-acetyltransferase	193.69	310.53	0.6818	4.6E-05
U6115_09690		GNAT family N-acetyltransferase	1160.71	2332.91	1.0061	2.7E-19
U6115_09695		DUF3313 domain-containing protein	7834.40	17774.88	1.1819	3.4E-24
U6115_09700		hypothetical protein	8288.37	8430.83	0.0246	9.3E-01
U6115_09705		hypothetical protein	155.42	457.02	1.5544	7.3E-17
U6115_09710		hypothetical protein	1044.89	17971.71	4.1047	2.5E-157
U6115_09715		3'-5' exonuclease	243.65	331.33	0.4469	5.3E-03

U6115_09720		putative nucleotidyltransferase substrate binding domain-containing protein	1990.76	2038.06	0.0344	7.8E-01
U6115_09725		class II glutamine amidotransferase	7309.86	5061.58	-0.5302	6.3E-03
U6115_09730		LysR substrate-binding domain-containing protein	790.75	664.98	-0.2492	7.5E-02
U6115_09735		MBL fold metallo-hydrolase	431.91	1257.78	1.5425	4.7E-26
U6115_09740		DsbA family protein	259.24	782.68	1.5938	7.2E-18
U6115_09745		S41 family peptidase	408.95	747.16	0.8712	3.4E-06
U6115_09750		glycosyl hydrolase family 18 protein	1134.29	1424.77	0.3283	1.2E-02
U6115_09755		peptidylprolyl isomerase	27642.54	14924.94	-0.8892	1.8E-08
U6115_09760		peptidylprolyl isomerase	66609.98	55894.45	-0.253	9.9E-02
U6115_09765		peptidyl-prolyl cis-trans isomerase	24056.16	22720.32	-0.0825	6.4E-01
U6115_09770		BolA family protein	865.44	621.09	-0.4794	1.2E-01
U6115_09775		YciI family protein	4589.68	5651.42	0.3002	5.4E-02
U6115_09780		septation protein A	8010.59	8273.81	0.0468	7.1E-01
U6115_09785		stomatin-like protein	10802.50	19409.80	0.8454	1.4E-04
U6115_09790		hypothetical protein	6105.72	9457.55	0.6314	1.1E-08
U6115_09795		hypothetical protein	2262.12	5870.51	1.376	4.2E-33
U6115_09800		YigZ family protein	608.37	621.05	0.0309	8.6E-01
U6115_09805		exopolyphosphatase	2841.80	3277.39	0.2061	8.6E-02
U6115_09810		HAD family phosphatase	3745.65	4314.89	0.2042	1.3E-01
U6115_09815		ParA family protein	2838.45	2985.83	0.0734	5.9E-01
U6115_09820		GMP reductase	5973.65	4284.34	-0.4796	1.2E-04
U6115_09825		MaoC family dehydratase	4226.78	6401.40	0.5993	1.3E-07
U6115_09830		alpha/beta fold hydrolase	790.27	2727.73	1.7865	8.3E-30
U6115_09835		GNAT family protein	1099.23	3078.75	1.486	8.9E-27
U6115_09840		[protein-PII] uridylyltransferase	11125.06	10839.64	-0.0374	7.5E-01
U6115_09845	lptG	LPS export ABC transporter permease LptG	7818.33	5581.38	-0.4863	4.9E-04
U6115_09850	lptF	LPS export ABC transporter permease LptF	17747.97	9813.05	-0.8549	5.5E-07
U6115_09855		leucyl aminopeptidase	22517.51	18966.57	-0.2476	2.3E-02
U6115_09860		DNA polymerase III subunit chi	3508.55	3320.51	-0.0792	6.8E-01
U6115_09865		hypothetical protein	7387.74	6938.14	-0.0906	6.7E-01
U6115_09870		cob(I)yrinic acid a,c-diamide adenosyltransferase	3296.85	2792.04	-0.2395	1.3E-01
U6115_09875		Na/Pi cotransporter family protein	2862.79	2327.43	-0.2988	4.0E-03

U6115_09880		transposase	101.70	66.54	-0.6132	1.7E-02
U6115_09885		IS5/IS1182 family transposase	925.30	513.44	-0.8495	4.0E-10
U6115_09890		tRNA-Thr	491.14	175.97	-1.4818	1.2E-07
U6115_09895		hypothetical protein	7424.54	3311.89	-1.1645	9.4E-09
U6115_09900		hypothetical protein	90.30	172.89	0.9434	4.7E-06
U6115_09905		hypothetical protein	1740.54	2446.48	0.4904	2.8E-03
U6115_09910	nudC	NAD(+) diphosphatase	6463.89	8015.93	0.3103	9.0E-03
U6115_09915		YbdK family carboxylate-amine ligase	6445.45	26806.71	2.0563	8.9E-39
U6115_09920		cation:proton antiporter	7387.66	25016.13	1.7595	4.5E-50
U6115_09925		M90 family metallopeptidase	1679.38	2691.22	0.6804	4.5E-04
U6115_09930		MFS transporter	2912.61	5097.49	0.8076	4.0E-11
U6115_09935		carbohydrate ABC transporter permease	746.25	933.50	0.3226	1.7E-01
U6115_09940		sugar ABC transporter permease	616.70	707.94	0.1976	4.3E-01
U6115_09945		extracellular solute-binding protein	840.93	2165.85	1.3642	7.5E-09
U6115_09950		BadF/BadG/BcrA/BcrD ATPase family protein	506.50	1019.35	1.0083	3.4E-06
U6115_09955		ROK family transcriptional regulator	702.88	951.28	0.4362	2.6E-02
U6115_09960		glycoside hydrolase family 9 protein	1546.32	3187.52	1.0433	5.3E-09
U6115_09965		hypothetical protein	3313.18	3788.31	0.193	3.2E-01
U6115_09970		DEAD/DEAH box helicase	66844.35	27162.64	-1.2992	1.1E-17
U6115_09975		EAL domain-containing protein	253.98	486.67	0.9358	9.0E-06
U6115_09980		hypothetical protein	2483.02	1716.09	-0.5325	3.6E-04
U6115_09985		response regulator	11179.48	8240.61	-0.4401	2.5E-02
U6115_09990	flgB	flagellar basal body rod protein FlgB	16911.21	38789.44	1.1977	5.0E-10
U6115_09995	flgC	flagellar basal body rod protein FlgC	7554.31	18819.35	1.3169	1.4E-24
U6115_10000		flagellar hook capping FlgD N-terminal domain-containing protein	26622.43	95493.37	1.8428	3.1E-26
U6115_10005	flgE	flagellar hook protein FlgE	42982.40	185940.33	2.113	2.0E-29
U6115_10010		flagellar basal body rod protein FlgF	8877.23	35178.98	1.9864	4.1E-67
U6115_10015	flgG	flagellar basal-body rod protein FlgG	12906.86	43960.18	1.768	1.1E-26
U6115_10020		flagellar basal body L-ring protein FlgH	12975.49	46564.82	1.8435	5.2E-51
U6115_10025		flagellar basal body P-ring protein FlgI	11148.98	33935.48	1.6059	8.8E-30
U6115_10030	flgJ	flagellar assembly peptidoglycan hydrolase FlgJ	25575.61	69076.51	1.4334	7.7E-36
U6115_10035	flgK	flagellar hook-associated protein FlgK	33149.55	112220.78	1.7593	5.9E-28

U6115_10040	flgL	flagellar hook-associated protein FlgL	21622.59	78244.89	1.8555	4.7E-17
U6115_10045	astE	succinylglutamate desuccinylase	4400.45	7502.04	0.7699	1.3E-10
U6115_10050	speA	arginine decarboxylase	25710.47	19933.76	-0.3671	1.2E-02
U6115_10055		GNAT family N-acetyltransferase	2578.05	1806.91	-0.5123	4.5E-05
U6115_10060		L-serine ammonia-lyase	17506.07	13251.40	-0.4017	2.0E-03
U6115_10065		YqaA family protein	1537.98	4091.12	1.4115	1.0E-31
U6115_10070	aqpZ	aquaporin Z	431.61	537.63	0.3127	5.5E-02
U6115_10075		LLM class flavin-dependent oxidoreductase	2075.62	2506.80	0.2718	5.4E-02
U6115_10080		nuclear transport factor 2 family protein	1878.98	5649.08	1.5889	5.8E-16
U6115_10085		7-cyano-7-deazaguanine/7-aminomethyl-7-deazaguanine transporter	4246.08	2333.88	-0.8634	2.4E-13
U6115_10090		methyl-accepting chemotaxis protein	663.37	1314.37	0.9866	1.9E-11
U6115_10095		hypothetical protein	171.07	130.46	-0.3976	7.3E-02
U6115_10100		hypothetical protein	213.78	180.32	-0.2517	3.2E-01
U6115_10105		WbuC family cupin fold metalloprotein	946.98	716.12	-0.4027	1.8E-01
U6115_10110		PAS domain-containing protein	2983.90	3251.65	0.1241	6.7E-01
U6115_10115	tauA	taurine ABC transporter substrate-binding protein	2501.74	9847.72	1.9768	7.2E-33
U6115_10120	tauB	taurine ABC transporter ATP-binding subunit	559.97	2720.86	2.2797	8.0E-34
U6115_10125	tauC	taurine ABC transporter permease TauC	448.49	1828.62	2.0281	2.2E-30
U6115_10130	tauD	taurine dioxygenase	1075.78	4562.80	2.0838	6.8E-28
U6115_10135	dbpA	ATP-dependent RNA helicase DbpA	5297.41	3745.36	-0.5002	1.3E-03
U6115_10140		methyl-accepting chemotaxis protein	1154.45	2265.97	0.973	3.2E-14
U6115_10145		TIGR03862 family flavoprotein	986.18	808.51	-0.2865	1.6E-01
U6115_10150		YkgJ family cysteine cluster protein	369.72	421.28	0.1906	3.6E-01
U6115_10155		alpha/beta hydrolase	444.26	724.01	0.7029	4.0E-06
U6115_10160		efflux transporter outer membrane subunit	633.16	984.34	0.6365	2.0E-04
U6115_10165		efflux RND transporter periplasmic adaptor subunit	367.85	489.53	0.411	1.7E-02
U6115_10170		DUF1656 domain-containing protein	48.55	55.17	0.2035	5.4E-01
U6115_10175		FUSC family protein	1916.73	1680.26	-0.1905	4.1E-01
U6115_10180		MarR family winged helix-turn-helix transcriptional regulator	724.85	388.78	-0.9008	9.6E-07
U6115_10185		calcium-binding protein	7765.80	3832.67	-1.0192	1.0E-08
U6115_10190		sigma factor-like helix-turn-helix DNA-binding protein	193.18	421.10	1.1275	7.2E-09

U6115_10195		aspartate aminotransferase family protein	922.26	1393.49	0.5956	6.9E-06
U6115_10200		formyltransferase family protein	1504.43	1300.36	-0.2105	1.3E-01
U6115_10205		type I secretion system permease/ATPase	2669.36	1317.20	-1.0189	4.2E-14
U6115_10210		HlyD family type I secretion periplasmic adaptor subunit	757.63	422.55	-0.8439	7.0E-08
U6115_10215		glycosyltransferase family 4 protein	1208.01	857.82	-0.495	8.8E-05
U6115_10220		calcium-binding protein	657.98	446.14	-0.5637	3.3E-04
U6115_10225		PhoH family protein	13338.39	20105.73	0.5919	6.1E-04
U6115_10230		peroxiredoxin	10021.74	7032.51	-0.5111	3.9E-03
U6115_10235		Smr/MutS family protein	5123.89	5046.48	-0.022	9.1E-01
U6115_10240	trxB	thioredoxin-disulfide reductase	23791.25	29892.96	0.3294	1.1E-02
U6115_10245		putative motility protein	3184.00	4102.65	0.3659	6.1E-02
U6115_10250		ester cyclase	428.13	604.35	0.4965	3.6E-03
U6115_10255		SDR family oxidoreductase	308.59	511.13	0.7255	3.1E-06
U6115_10260		polysaccharide deacetylase family protein	1265.27	1562.91	0.3044	1.5E-02
U6115_10265	ampH	D-alanyl-D-alanine-carboxypeptidase/endopeptidase AmpH	6579.10	4971.35	-0.4042	1.1E-03
U6115_10270		DUF2938 domain-containing protein	684.90	858.95	0.3262	4.0E-02
U6115_10275		metalloregulator ArsR/SmtB family transcription factor	347.85	310.15	-0.1667	3.8E-01
U6115_10280		hypothetical protein	350.81	244.96	-0.519	2.0E-03
U6115_10285		DUF4440 domain-containing protein	509.76	491.92	-0.05	8.1E-01
U6115_10290		LysR family transcriptional regulator	1135.47	1332.79	0.2312	1.4E-01
U6115_10295		aminopeptidase P family protein	6396.44	11497.40	0.846	1.9E-08
U6115_10300		hypothetical protein	30681.60	40749.43	0.4094	5.9E-03
U6115_10305		VOC family protein	2134.68	2423.54	0.1824	2.2E-01
U6115_10310		flavodoxin family protein	1862.44	1983.42	0.0902	6.6E-01
U6115_10315	soxR	redox-sensitive transcriptional activator SoxR	635.11	798.63	0.3304	8.1E-03
U6115_10320	rfbB	dTDP-glucose 4,6-dehydratase	6926.11	6490.12	-0.0939	4.9E-01
U6115_10325	rfbD	dTDP-4-dehydrorhamnose reductase	3549.86	3495.88	-0.0217	8.7E-01
U6115_10330	rfbA	glucose-1-phosphate thymidylyltransferase RfbA	4692.74	5847.24	0.3174	3.5E-02
U6115_10335	rfbC	dTDP-4-dehydrorhamnose 3,5-epimerase	2277.50	3302.02	0.5354	4.8E-06
U6115_10340		acetyl-CoA C-acetyltransferase	15316.79	86222.73	2.493	6.7E-68
U6115_10345	phaC	class I poly(R)-hydroxyalkanoic acid synthase	23220.11	105459.83	2.1833	1.0E-32
U6115_10350		fumarylacetoacetate hydrolase family protein	3990.52	3754.53	-0.0879	6.2E-01

U6115_10355		S4 domain-containing protein	1046.31	1164.29	0.1547	3.9E-01
U6115_10360		MFS transporter	2172.06	2408.07	0.1486	1.9E-01
U6115_10365		nuclear transport factor 2 family protein	522.56	494.08	-0.0807	5.9E-01
U6115_10370		helix-turn-helix transcriptional regulator	2341.42			
