# Supplementary material for: Quorum Sensing in Chromobacterium subtsugae ATCC 31532 (Formerly Chromobacterium violaceum ATCC 31532): Transcriptomic and Genomic Analyses
Source: Microorganisms. 2025 Apr 29;13(5):1021. doi: 10.3390/microorganisms13051021 (PMC12114271; doi:10.3390/microorganisms13051021)
Supplement: Supplementary file 1 [file microorganisms-13-01021-s001.zip › Supplement_S2.pdf]

Quorum Sensing in *Chromobacterium subtsugae* ATCC 31532 (formerly - *Chromobacterium violaceum* ATCC 31532): Transcriptomic and Genomic Analyses

Supplement S2. DNA sequences of promoter/intergenic regions located upstream QS-controlled genes in *C. subtsugae* ATCC 31532 strain

>NZ_GP142381.1:39409-39509 **U6115_RS00160**
GTCGTAAAGTGAGTCGCTACGCAGAACTTCAACTAGAACACAGTAGTGTAAGGTAGAAAGAACTAGATTCCA
TTCGTTTGATCCGCTACGGCGGGCGCCG

>NZ_GP142381.1:40664-40864 **U6115_RS00165**
GCACAGTCATGGGGTGGGTGGTAGGTCAGCTAACGGGGACGGGCGCAGATCGGTCGCGAACACTTTAGGTG
AAGTAAGTACGGTGGGGTAAGTTCACTTTTGCTTGGCTAGAGCGGCCTTCTACCGACACGCGGGAGAGGGCA
GAGGGTGGAGGCGCGTCGCGGGACGAGCGGTCTACGCGCTGTTTTTGTAGCGCCGTA

>NZ_CP142381.1:40806-41006 **U6115_RS00170**
TCTCCCACCTCCGCGCAGCGCCCTGCTCGCCAGATGCGCGACAAAAACATCGCGGCATGCGGACGAAAGCCG
GCCTTTGCTAGACAAGCATGGCGCTAGCTGGTAATTTTTGTAGCAGCTGATACATAAATCGAAATATGCCGCC
CAAATATCTAAATGTCAACTATTTATGTAGTGGAAAGTAAAGAAAGGAAAAATAT

>NZ_CP142381.1:799824-800024 **U6115_RS03980**
ATCGTGCTGTGCTCCACCCCCAAGGCCCGCGCCCCGGCCGACAGGCTGCCTTCGCGCGCCAGCGCCAGAAAAT
AACGCACATCGTCCCATTGCATGGCAGTCCGATCATAAATGCACAGATGTGGCCAATTTATTCGACTTATGCCG
AATAAATCAAGGCGCTACACTGACGGCATCTTCATTTCACGCGAGCGGCCGAT

>NZ_GP142381.1:834076-834276 **U6115_RS04155**
CTACACCTGAGGACACTTTAGCCTACTGCCAACTACCGCGGCCATTCGCGCGTAGGGTAAACGAACGGCACCG
CGGTGCCATCGCCGCAGTGGGCGAGAAATATATTGCTTACCAAAATAAAAAGCGGCGGTAAATCCGGTCAAC
CCGTAAATCACGCCCACCCCTACCAGCCGCCGGCCCGCCATAGGTGCCGTTGGTA

>NZ_CP142381.1:943021-943221 **U6115_RS04630**
GTTTTGGGTAGTGTGATGCGAATTGGTAACAGCTTGAGATGCAAAGTTCTGTCTATGCATTATTTAGCTTGCTA
AATGATAGCCGAGAAAATATCCTATGATCATGAGCTGAGACACAAGGTGGGCTCAGCGTTTGGCAGTGCTCG
GATCGTGAGTCAGCATTATCTGCGTAGACTGATCAAGCAGGCGTTTGCTGCCGC

>NZ_GP142381.1:991139-991339 **U6115_RS04840**
AACAATTACTATTGATAATAGTAAACTTAGCCGTTCGTCCTTCAGTACGGCCTTCTTACGGCGCGCGCTTTAGC
CGGCGCGCCAGGGTCCACCGTTACGCCTCGCCCACTCCCGTCGCGTACGCGTTGAAGTTCCTTTAACAGGTTC
GTCGCGCAGGCGCGCGGCTAAACGGCGGGCGTCGGCCCGCTAAAAGTCTGTAT

>NZ_CP142381.1:1061107-1061307 **U6115_RS05110**
TAACAATGGTTTAGCGGGATGCTGTTGTTTTTTTGGTAATTTTCACTGGTCTTTCCTGGGTGAGCTTTGTTTTTT
TGCAACGCCGGTCTTATATTGCTTTCAGCGATCGGCGGAATATACCGTCGGCACATAAATGTCCGGACAGACG
GCGCTGCCGCGAAGTCAACAGGACAAAATCAACTGACTTGAAAGGATCTGCA

>NZ_GP142381.1:1275732-1275932 **U6115_RS06250**
CCGCCAAAGGAAATAAACACACACCACGGTCGTTCACCAATACCTAGTTTTTATAACTACGGAACAAGCCAAA
AACGACCACAGAACAATGCCTTAAGTAAGATAACGCCGAAACTTGCGCTATTTTAGCTTTTCTTGGCTTGTTTC
ACAAGTTTTTTTTACTTACTCACCGCGTCTACTTTGACTGAAACCTTCGAGAC

>NZ_CP142381.1:1631718-1631918 **U6115_RS08050**

CATTTTTAATTAGAATTAAAAATTCAAAATGACTAATGGCGGGCGGCTTGCCGCCCGCCGGGAAACGGCAGCC
ACAGCGGCGCGCGGTTCCCGATTCTGCAAGACAAAGAGCAAGAGCTGAGCCATTCCCCTTCAGATTTTGACCC
GTCGGCGGCCGCGCCGCCGGCGGGTGTCGATTGAATGAACCAAGGAATTTCGTG

>NZ_GP142381.1:1958439-1958639 **U6115_RS09710**
CAACGGAGAGGAACTCTTCTACAAACGGCCCGTCTCGAAGTGCCTAGGCGAGCACAGGAACTGCCTCCGCTC
CGGCGGCCGAACGGCGACCTGACCGTTAAGAACGGCGGCGCGGCGGGCAGGAACGGCGGCAAAGGCCAAG
TTCGCCGACCTAAAGGCGAAAAAGAACGTCCTTGTTACTGAACCGCTACACCCAAACT

>NZ_CP142381.1:2141163-2141363 **U6115_RS10635**
CGGGCGTTTGGTTTGCCTGGGCTGCGGGCGAGGCAGATTGATGTATTTGTCATTCCATTTCGTCAGAAAGCGC
GGGCTTTGATAGCATTACTAATACTATCAATTCATGAACTTTCTCAATAAATATGCATCTGTAATCTTGTCGGGG
CGTTTGCCTTGGCGTGACCGTGTTTTCTTGATAACTACACAGGAGATGCAAA

>NZ_CP142381.1:2761196-2761396 **U6115_RS13245**
GCCGCGTCCCCAGCCCAAGCTGACCCCGCCTCGCGCCCCGCCTTTTCCCAAGCCTCGCCAGCCATCTACCTGTA
CTTAAGGGCAGAGCAGGAACCGGGCAAGCTGCTAATCAGAAAGCACTGCCCGGCGCCCGCCCGTCACTTCAC
CGCGGGTTGCCGCGCACGTCCCCTGCCTTTTTCAATTTCCGAGAGAAAGCGAAC

>NZ_CP142381.1:3014368-3014568 **U6115_RS14430**
GTATCGCGCGGGTGGTGATGTCGAACTCCTGCGCCAGTTCGGTGATGTTGAAAACCTGCTCCGCCATGAATGC
CGCTCCCTGCTGTCGCTCTGCGGCCATGGGCGCCACAGTTGGCTGGTTTATGCTCATTGTCTGTTGACGTTGAC
GTAAACGTCAAGTTATCGTGACCACAATAGAGCAAATAATCCAGGAGAGCCAG

>NZ_GP142381.1:3081893-3082093 **U6115_RS14770**
ACCTTACAGGAGGTCCTGTCCGCGAGGTTTGTTTTACGGCAAAACGTCGACCACGTTGCCCAAACGCGGCGTA
TCGTACTTGTCGCCTATTAGGTGCTTTCCTTCGTTTGGTACTGCTACCTGGACCTTTGGGCCGACGTTCGCGTTT
AGTGGCGCGTCTTGGACTACTAACTCCGCGACGACCTTCGGCGGTAGGTCCG

>NZ_CP142381.1:3093693-3093893 **U6115_RS14865**
AGCAAAAAATAGCAATACTTATCATTTGCTGCAATGTGTTGTTGATGGAGCTGCTTTTTTATTTCACTCATCTCT
TACATCTCCTGAGTCATCTCGATATCCCTAGCTATTAAGCAGCTAGGCTCCCTACGGCACAAGCTCAGCATAAC
TTTAAAGCTGATTAAAAGTCTCGCCTGCTGACACCGTTCACCATGAACGGC

>NZ_GP142381.1:3160464-3160658 **U6115_RS15190**
CCGCCGTGGTGCTGCGGACTCCGCAGGCTTGTAACGCAGGGGTAGCCGCTTCGCCGAAGCGGCTGTGGTAGT
CTGGCGGGCCGGGCCCGCCTGTTACAGGGTGCCGACAGGCCGGCAGGGCCGCCGCGGCCCGCCGCCTGATA
TCAACACGCTATGTCTGTTCTGGCCTGCCGCAGTAGGCCGGCCTTCGGCCG

>NZ_GP142381.1:3161759-3161959 **U6115_RS15195**
CTGTCCGAAGGATCCATTCGTGCTGGTATCTGCGAAGGGGGCTTAGGTCCCCTTCGTTTCTTGGCGTAACAAA
GGATGGCGGGCGCGGCGCGGCCGCGATGACGGTTGTATGTGGGACCCGTCCGCGCGCGGTAGCGGCCGCG
CAATGCCGTACATTTGGCCTAGCGTAGGCCGTACGCGCGTTAGCCCGTCCTTAAGTC

>NZ_GP142381.1:3357685-3357885 **U6115_RS16120**
CCACTAAGGAGGTCCACTACGGTTGGGGCGACCGGCAGGCCGGCGCGCGCCCGGCCTTGCGTGTTACGCCGC
CCCTTAGCGCTAACAAGTTCGTAGCTTTACGAAAGCGGGGGTAGTGGGATTCGGTAGCGAGGCCGGCCGTGT
GGCCATGGTTCCCGATCATGGGGACCCGTCCGTTTGGAGCCTACTAACGGGTGGGC
